# Supplementary material for: Amyloid-β disrupts APP-regulated protein aggregation and dissociation from recycling endosomal membranes
Source: EMBO J. 2025 Jul 17;44(16):4443–72. doi: 10.1038/s44318-025-00497-y (PMC12361456; doi:10.1038/s44318-025-00497-y)
Supplement: Supplementary file 1 — Appendix [file 44318_2025_497_MOESM1_ESM.pdf]

# Appendix for 'Aβ disrupts APP-regulated protein aggregation and dissociation from recycling endosomal membranes'

## Table of Contents

**Appendix Tables**

Appendix Table S1 2

Appendix Table S2 3

**Appendix Figures**

Appendix Figure S1 7

Appendix Figure S2 9

Appendix Figure S3 10

Appendix Figure S4 12

Appendix Figure S5 14

Appendix Figure S6 16

Appendix Figure S7 17

Appendix Figure S8 19

Appendix Figure S9 21

## Appendix Tables

### Appendix Table S1

| Gene | BDSC stock | Expression pattern |
|---|---|---|
| *Ance* | 59828 - *w*$^{1118}$; *GFP-Ance*$^{MI05748\text{-}GFSTF}$*/SM6a* | Secondary cells (weak) |
| *cert* | 60544 - *w*$^{1118}$;; *GFP-cert*$^{MI04831\text{-}GFSTF.0}$*/TM6C, Sb Tb* | Not detected |
| *CG30438* | 61793 - *w*$^{1118}$; *GFP-CG30438*$^{MI07302\text{-}GFSTF.1}$ | Main cells and AG lumen |
| *comm3* | 61791 - *w*$^{1118}$;; *GFP-comm3*$^{MI06957\text{-}GFSTF.1}$*/TM6C, Sb Tb* | Not detected |
| *Cyp4p3* | 59829 - *w*$^{1118}$; *GFP-Cyp4p3*$^{MI05774\text{-}GFSTF.1}$ */CyO* | Not detected |
| *dpr3* | 60243 - *w*$^{1118}$; *GFP-dpr3*$^{MI05963\text{-}GFSTF.1}$*/SM6a* | Not detected |
| *mfas* | 63204 - *w*$^{1118}$;; *GFP-mfas*$^{MI11275\text{-}GFSTF.2}$ | Secondary cells (strong) and AG lumen |
| *Pkc53E* | 59413 - *w*$^{1118}$; *GFP-Pkc53E*$^{MI05296\text{-}GFSTF.0}$ | Not detected |
| *Tsp42Ee* | 63197 - *w*$^{1118}$; *GFP-Tsp42Ee*$^{MI09912\text{-}GFSTF.0}$ | Ejaculatory duct |
| *unc-104* | 59302 - *w*$^{1118}$; *GFP-unc-104*$^{MI07215\text{-}GFSTF.1}$*/SM6a* | Not detected |

### Appendix Table S1 Gene trap lines tested for accessory gland expression

*Drosophila* lines (Nagarkar-Jaiswal et al, 2015) were selected on the basis that the labelled genes were reported to be transcribed at high levels in the male accessory gland (Leader et al, 2018), or in the case of ANCE, it was already known to be a DCG component (Rylett et al, 2007).

## Appendix Table S2

## Increased

| 1 | Accession | Description | # Unique Peptides | mean fold change | stdev | ttest | log 2 fold change (median) |
|---|---|---|---|---|---|---|---|
| 2 | P26022 | Pentraxin-related protein PTX3 OS=Homo sapiens OX=9606 GN=PTX3 PE=1 SV=3 | 2 | 3.103026283 | 1.3115 | 0.0490622 | 1.633675917 |
| 3 | Q14103 | Heterogeneous nuclear ribonucleoprotein D0 OS=Homo sapiens OX=9606 GN=HNRNPD PE=1 SV=1 | 3 | 1.663045524 | 0.3041 | 0.0222886 | 0.733827662 |
| 4 | P24534 | Elongation factor 1-beta OS=Homo sapiens OX=9606 GN=EEF1B2 PE=1 SV=3 | 4 | 1.49994302 | 0.1124 | 0.0029924 | 0.584907697 |
| 5 | P53618 | Coatomer subunit beta OS=Homo sapiens OX=9606 GN=COPB1 PE=1 SV=3 | 1 | 1.495738135 | 0.2661 | 0.0336738 | 0.580857619 |
| 6 | P26639 | Isoform 2 of Threonine--tRNA ligase, cytoplasmic OS=Homo sapiens OX=9606 GN=TARS | 11 | 1.455463268 | 0.1124 | 0.0039242 | 0.541478431 |
| 7 | O75643 | U5 small nuclear ribonucleoprotein 200 kDa helicase OS=Homo sapiens OX=9606 GN=SNRNP200 PE=1 SV=2 | 4 | 1.439711872 | 0.1937 | 0.0199982 | 0.525780116 |
| 8 | A0A0U1RRN | Protein enabled homolog OS=Homo sapiens OX=9606 GN=ENAH PE=1 SV=1 | 1 | 1.434448199 | 0.2641 | 0.0460793 | 0.52049587 |
| 9 | P09651 | Heterogeneous nuclear ribonucleoprotein A1 OS=Homo sapiens OX=9606 GN=HNRNPA1 PE=1 SV=5 | 3 | 1.42824799 | 0.253 | 0.0429211 | 0.514246499 |
| 10 | P59998 | Isoform 3 of Actin-related protein 2/3 complex subunit 4 OS=Homo sapiens OX=9606 GN=ARPC4 | 1 | 1.421560857 | 0.1598 | 0.0132799 | 0.507475862 |
| 11 | Q16658 | Fascin OS=Homo sapiens OX=9606 GN=FSCN1 PE=1 SV=3 | 8 | 1.416567441 | 0.1335 | 0.0083033 | 0.502399289 |
| 12 | H0YMV8 | 40S ribosomal protein S27 OS=Homo sapiens OX=9606 GN=RPS27L PE=1 SV=1 | 1 | 1.404460801 | 0.1232 | 0.0071859 | 0.490016359 |
| 13 | P55060 | Exportin-2 OS=Homo sapiens OX=9606 GN=CSE1L PE=1 SV=3 | 6 | 1.379513008 | 0.2146 | 0.0384422 | 0.464159061 |
| 14 | B5ME19 | Eukaryotic translation initiation factor 3 subunit C-like protein OS=Homo sapiens OX=9606 GN=EIF3CL PE=3 SV=1 | 3 | 1.368279987 | 0.1884 | 0.0297218 | 0.452363475 |
| 15 | P49773 | Histidine triad nucleotide-binding protein 1 OS=Homo sapiens OX=9606 GN=HINT1 PE=1 SV=2 | 1 | 1.353572134 | 0.1027 | 0.0062799 | 0.436771773 |
| 16 | P45974 | Ubiquitin carboxyl-terminal hydrolase 5 OS=Homo sapiens OX=9606 GN=USP5 PE=1 SV=2 | 2 | 1.351847432 | 0.1686 | 0.0250291 | 0.43493234 |
| 17 | P61081 | NEDD8-conjugating enzyme Ubc12 OS=Homo sapiens OX=9606 GN=UBE2M PE=1 SV=1 | 1 | 1.348671223 | 0.1446 | 0.0169825 | 0.431538693 |
| 18 | P29401 | Isoform 2 of Transketolase OS=Homo sapiens OX=9606 GN=TKT | 12 | 1.34791125 | 0.0797 | 0.0031615 | 0.430725509 |
| 19 | F6WQW2 | Ran-specific GTPase-activating protein OS=Homo sapiens OX=9606 GN=RANBP1 PE=1 SV=1 | 2 | 1.327522787 | 0.1073 | 0.0088206 | 0.408736625 |
| 20 | Q02790 | Peptidyl-prolyl cis-trans isomerase FKBP4 OS=Homo sapiens OX=9606 GN=FKBP4 PE=1 SV=3 | 4 | 1.325741745 | 0.1407 | 0.0189549 | 0.406799765 |
| 21 | P22626 | Heterogeneous nuclear ribonucleoproteins A2/B1 OS=Homo sapiens OX=9606 GN=HNRNPA2B1 PE=1 SV=2 | 9 | 1.323716605 | 0.0737 | 0.0031044 | 0.404594288 |
| 22 | P08238 | Heat shock protein HSP 90-beta OS=Homo sapiens OX=9606 GN=HSP90AB1 PE=1 SV=4 | 10 | 1.32080685 | 0.0536 | 0.001252 | 0.401419508 |
| 23 | P30041 | Peroxiredoxin-6 OS=Homo sapiens OX=9606 GN=PRDX6 PE=1 SV=3 | 7 | 1.310524283 | 0.0868 | 0.005616 | 0.390144086 |
| 24 | Q9UQ80 | Proliferation-associated protein 2G4 OS=Homo sapiens OX=9606 GN=PA2G4 PE=1 SV=3 | 2 | 1.305159235 | 0.0459 | 0.0009192 | 0.384225832 |
| 25 | O43390 | Isoform 2 of Heterogeneous nuclear ribonucleoprotein R OS=Homo sapiens OX=9606 GN=HNRNPR | 2 | 1.292639143 | 0.1077 | 0.0122254 | 0.370319585 |
| 26 | P26368 | Splicing factor U2AF 65 kDa subunit OS=Homo sapiens OX=9606 GN=U2AF2 PE=1 SV=4 | 1 | 1.284586327 | 0.1429 | 0.0283137 | 0.361303846 |
| 27 | P14625 | Endoplasmin OS=Homo sapiens OX=9606 GN=HSP90B1 PE=1 SV=1 | 9 | 1.28311914 | 0.1739 | 0.0472718 | 0.359655134 |
| 28 | P00558 | Phosphoglycerate kinase 1 OS=Homo sapiens OX=9606 GN=PGK1 PE=1 SV=3 | 19 | 1.280577008 | 0.1706 | 0.0460777 | 0.356794013 |
| 29 | J3KQE5 | GTP-binding nuclear protein Ran (Fragment) OS=Homo sapiens OX=9606 GN=RAN PE=1 SV=1 | 3 | 1.263417237 | 0.0465 | 0.001474 | 0.337331161 |
| 30 | A0A2R8YEI | Peripheral plasma membrane protein CASK OS=Homo sapiens OX=9606 GN=CASK PE=1 SV=1 | 1 | 1.263171553 | 0.1637 | 0.0487656 | 0.337050586 |
| 31 | Q13155 | Aminoacyl tRNA synthase complex-interacting multifunctional protein 2 OS=Homo sapiens OX=9606 GN=AIMP2 PE=1 S | 1 | 1.254188096 | 0.1389 | 0.0352146 | 0.326753732 |
| 32 | Q15046 | Isoform Mitochondrial of Lysine--tRNA ligase OS=Homo sapiens OX=9606 GN=KARS | 2 | 1.253934466 | 0.0757 | 0.0067461 | 0.326461951 |
| 33 | O75116 | Rho-associated protein kinase 2 OS=Homo sapiens OX=9606 GN=ROCK2 PE=1 SV=4 | 2 | 1.252176666 | 0.1063 | 0.0177663 | 0.324438122 |
| 34 | P17931 | Galectin-3 OS=Homo sapiens OX=9606 GN=LGALS3 PE=1 SV=5 | 2 | 1.222396554 | 0.1005 | 0.0214133 | 0.289712381 |
| 35 | Q06830 | Peroxiredoxin-1 OS=Homo sapiens OX=9606 GN=PRDX1 PE=1 SV=1 | 9 | 1.221926774 | 0.0996 | 0.0210149 | 0.289157832 |
| 36 | P14618 | Pyruvate kinase PKM OS=Homo sapiens OX=9606 GN=PKM PE=1 SV=4 | 3 | 1.220007075 | 0.0338 | 0.0009777 | 0.286889514 |
| 37 | P52907 | F-actin-capping protein subunit alpha-1 OS=Homo sapiens OX=9606 GN=CAPZA1 PE=1 SV=3 | 3 | 1.218824953 | 0.1013 | 0.0228326 | 0.285490942 |
| 38 | P52209 | 6-phosphogluconate dehydrogenase, decarboxylating OS=Homo sapiens OX=9606 GN=PGD PE=1 SV=3 | 11 | 1.214153918 | 0.115 | 0.0336931 | 0.279951323 |
| 39 | Q16531 | DNA damage-binding protein 1 OS=Homo sapiens OX=9606 GN=DDB1 PE=1 SV=1 | 4 | 1.210899345 | 0.1119 | 0.032683 | 0.276078947 |
| 40 | P13639 | Elongation factor 2 OS=Homo sapiens OX=9606 GN=EEF2 PE=1 SV=4 | 22 | 1.204031433 | 0.0997 | 0.0263592 | 0.267873056 |
| 41 | P07900 | Isoform 2 of Heat shock protein HSP 90-alpha OS=Homo sapiens OX=9606 GN=HSP90AA1 | 12 | 1.203044578 | 0.0916 | 0.0213479 | 0.266690102 |
| 42 | P0C0S8 | Histone H2A type 1 OS=Homo sapiens OX=9606 GN=HIST1H2AG PE=1 SV=2 | 3 | 1.200785297 | 0.096 | 0.0248945 | 0.263978217 |
| 43 | C9JIZ6 | Prosaposin OS=Homo sapiens OX=9606 GN=PSAP PE=1 SV=2 | 2 | 1.199107637 | 0.0914 | 0.0223471 | 0.261961167 |
| 44 | Q06323 | Isoform 2 of Proteasome activator complex subunit 1 OS=Homo sapiens OX=9606 GN=PSME1 | 2 | 1.175788725 | 0.0706 | 0.0155882 | 0.233628849 |
| 45 | Q15149 | Isoform 4 of Plectin OS=Homo sapiens OX=9606 GN=PLEC | 63 | 1.148327865 | 0.0711 | 0.0250619 | 0.199534613 |
| 46 | O75083 | WD repeat-containing protein 1 OS=Homo sapiens OX=9606 GN=WDR1 PE=1 SV=4 | 8 | 1.14194164 | 0.0376 | 0.0048048 | 0.191488922 |
| 47 | E9PK25 | Cofilin-1 OS=Homo sapiens OX=9606 GN=CFL1 PE=1 SV=1 | 6 | 1.141786467 | 0.0523 | 0.0123074 | 0.191292868 |
| 48 | P17987 | T-complex protein 1 subunit alpha OS=Homo sapiens OX=9606 GN=TCP1 PE=1 SV=1 | 11 | 1.136392873 | 0.0817 | 0.0444423 | 0.184461689 |
| 49 | P02794 | Ferritin heavy chain OS=Homo sapiens OX=9606 GN=FTH1 PE=1 SV=2 | 2 | 1.1360315 | 0.0734 | 0.0340847 | 0.184002839 |
| 50 | P22314 | Ubiquitin-like modifier-activating enzyme 1 OS=Homo sapiens OX=9606 GN=UBA1 PE=1 SV=3 | 20 | 1.13569203 | 0.0441 | 0.008612 | 0.183571667 |
| 51 | P30044 | Peroxiredoxin-5, mitochondrial OS=Homo sapiens OX=9606 GN=PRDX5 PE=1 SV=4 | 5 | 1.131247938 | 0.0736 | 0.0376284 | 0.177915162 |
| 52 | P53396 | ATP-citrate synthase OS=Homo sapiens OX=9606 GN=ACLY PE=1 SV=3 | 22 | 1.118980325 | 0.0556 | 0.0234826 | 0.16218467 |
| 53 | P07737 | Profilin-1 OS=Homo sapiens OX=9606 GN=PFN1 PE=1 SV=2 | 9 | 1.118484976 | 0.0726 | 0.0469787 | 0.161545877 |
| 54 | O75369 | Isoform 8 of Filamin-B OS=Homo sapiens OX=9606 GN=FLNB | 36 | 1.116898151 | 0.0447 | 0.01356 | 0.159497633 |
| 55 | P07814 | Bifunctional glutamate/proline--tRNA ligase OS=Homo sapiens OX=9606 GN=EPRS PE=1 SV=5 | 5 | 1.116750264 | 0.0598 | 0.029806 | 0.159306595 |
| 56 | Q86UP2 | Kinectin OS=Homo sapiens OX=9606 GN=KTN1 PE=1 SV=1 | 4 | 1.113553286 | 0.0333 | 0.0064424 | 0.155170596 |
| 57 | P11142 | Heat shock cognate 71 kDa protein OS=Homo sapiens OX=9606 GN=HSPA8 PE=1 SV=1 | 7 | 1.109865611 | 0.0594 | 0.0342839 | 0.150384997 |
| 58 | P48643 | T-complex protein 1 subunit epsilon OS=Homo sapiens OX=9606 GN=CCT5 PE=1 SV=1 | 17 | 1.102728895 | 0.0425 | 0.0169264 | 0.141078149 |
| 59 | Q96TA1 | Niban-like protein 1 OS=Homo sapiens OX=9606 GN=FAM129B PE=1 SV=3 | 6 | 1.08853694 | 0.023 | 0.0045581 | 0.122390367 |
| 60 | P18669 | Phosphoglycerate mutase 1 OS=Homo sapiens OX=9606 GN=PGAM1 PE=1 SV=2 | 3 | 1.084261619 | 0.0373 | 0.0203107 | 0.116712903 |
| 61 | P04075 | Isoform 2 of Fructose-bisphosphate aldolase A OS=Homo sapiens OX=9606 GN=ALDOA | 9 | 1.080223566 | 0.0175 | 0.0027579 | 0.111329927 |
| 62 | O43707 | Alpha-actinin-4 OS=Homo sapiens OX=9606 GN=ACTN4 PE=1 SV=2 | 16 | 1.074464085 | 0.022 | 0.0065808 | 0.10361726 |
| 63 | | | | | | | |
| 64 | P04406 | Glyceraldehyde-3-phosphate dehydrogenase OS=Homo sapiens OX=9606 GN=GAPDH PE=1 SV=3 | 13 | 1.154854946 | 0.0982 | 0.0510507 | 0.207711656 |

# Decreased

| 1 | Accession | Description | # Unique Peptides | mean fold change | stdev | ttest | log 2 fold change (median) |
|---|---|---|---|---|---|---|---|
| 2 | Q93008 | Probable ubiquitin carboxyl-terminal hydrolase FAF-X OS=Homo sapiens OX=9606 GN=USP9X PE=1 SV=3 | 1 | 0.212889624 | 0.38698 | 0.0268 | -2.231822458 |
| 3 | P07585 | Decorin OS=Homo sapiens OX=9606 GN=DCN PE=1 SV=1 | 14 | 0.455884771 | 0.06169 | 0.0004 | -1.133258879 |
| 4 | P05067 | Amyloid-beta A4 protein OS=Homo sapiens OX=9606 GN=APP PE=1 SV=3 | 24 | 0.470540539 | 0.13076 | 0.00394 | -1.087609073 |
| 5 | P61073-2 | Isoform 2 of C-X-C chemokine receptor type 4 OS=Homo sapiens OX=9606 GN=CXCR4 | 1 | 0.482755086 | 0.32108 | 0.04851 | -1.050636635 |
| 6 | P21810 | Biglycan OS=Homo sapiens OX=9606 GN=BGN PE=1 SV=2 | 8 | 0.482944849 | 0.10464 | 0.0022 | -1.050069648 |
| 7 | I3L4N8 | Actin, cytoplasmic 2 (Fragment) OS=Homo sapiens OX=9606 GN=ACTG1 PE=1 SV=8 | 9 | 0.512217566 | 0.14364 | 0.00653 | -0.965171365 |
| 8 | P10124 | Serglycin OS=Homo sapiens OX=9606 GN=SRGN PE=1 SV=3 | 5 | 0.517161401 | 0.05829 | 0.00048 | -0.951313492 |
| 9 | P10909-2 | Isoform 2 of Clusterin OS=Homo sapiens OX=9606 GN=CLU | 16 | 0.52103899 | 0.06784 | 0.00077 | -0.940536759 |
| 10 | Q08431 | Lactadherin OS=Homo sapiens OX=9606 GN=MFGE8 PE=1 SV=3 | 1 | 0.525917369 | 0.16566 | 0.01059 | -0.92709195 |
| 11 | Q9UHD8 | Septin-9 OS=Homo sapiens OX=9606 GN=SEPT9 PE=1 SV=2 | 1 | 0.533218514 | 0.22574 | 0.02566 | -0.907201222 |
| 12 | A1L4H1 | Soluble scavenger receptor cysteine-rich domain-containing protein SSC5D OS=Homo sapiens OX=9606 GN=SSC5D PE=1 SV=3 | 16 | 0.533720259 | 0.08099 | 0.00141 | -0.905844321 |
| 13 | Q08629 | Testican-1 OS=Homo sapiens OX=9606 GN=SPOCK1 PE=1 SV=1 | 10 | 0.534327844 | 0.06524 | 0.00074 | -0.904202895 |
| 14 | P09603 | Macrophage colony-stimulating factor 1 OS=Homo sapiens OX=9606 GN=CSF1 PE=1 SV=2 | 5 | 0.544437892 | 0.16509 | 0.01172 | -0.877160615 |
| 15 | Q5VWC4 | 26S proteasome non-ATPase regulatory subunit 4 OS=Homo sapiens OX=9606 GN=PSMD4 PE=1 SV=1 | 1 | 0.546421067 | 0.24537 | 0.03435 | -0.871914989 |
| 16 | Q08380 | Galectin-3-binding protein OS=Homo sapiens OX=9606 GN=LGALS3BP PE=1 SV=1 | 14 | 0.557341543 | 0.06938 | 0.00104 | -0.843366402 |
| 17 | P20908 | Collagen alpha-1(V) chain OS=Homo sapiens OX=9606 GN=COL5A1 PE=1 SV=3 | 36 | 0.566077926 | 0.19393 | 0.0208 | -0.820927428 |
| 18 | Q10589 | Bone marrow stromal antigen 2 OS=Homo sapiens OX=9606 GN=BST2 PE=1 SV=1 | 2 | 0.57264581 | 0.10498 | 0.00387 | -0.804285009 |
| 19 | Q02809 | Procollagen-lysine,2-oxoglutarate 5-dioxygenase 1 OS=Homo sapiens OX=9606 GN=PLOD1 PE=1 SV=2 | 23 | 0.573005487 | 0.04798 | 0.00039 | -0.80337914 |
| 20 | P98066 | Tumor necrosis factor-inducible gene 6 protein OS=Homo sapiens OX=9606 GN=TNFAIP6 PE=1 SV=2 | 7 | 0.57342002 | 0.09161 | 0.00262 | -0.802335821 |
| 21 | P01308 | Insulin OS=Homo sapiens OX=9606 GN=INS PE=1 SV=1 | 1 | 0.579047392 | 0.07908 | 0.00177 | -0.788246664 |
| 22 | P32004 | Neural cell adhesion molecule L1 OS=Homo sapiens OX=9606 GN=L1CAM PE=1 SV=2 | 6 | 0.582268702 | 0.07465 | 0.00153 | -0.780243021 |
| 23 | O43854 | EGF-like repeat and discoidin I-like domain-containing protein 3 OS=Homo sapiens OX=9606 GN=EDIL3 PE=1 SV=1 | 13 | 0.584275459 | 0.09076 | 0.00275 | -0.775279402 |
| 24 | P18827 | Syndecan-1 OS=Homo sapiens OX=9606 GN=SDC1 PE=1 SV=3 | 1 | 0.586156807 | 0.10114 | 0.00382 | -0.770641433 |
| 25 | Q92743 | Serine protease HTRA1 OS=Homo sapiens OX=9606 GN=HTRA1 PE=1 SV=1 | 16 | 0.586669735 | 0.08657 | 0.00244 | -0.769379526 |
| 26 | H3BTH8 | Hyaluronan and proteoglycan link protein 3 OS=Homo sapiens OX=9606 GN=HAPLN3 PE=1 SV=1 | 1 | 0.587541725 | 0.10512 | 0.00431 | -0.767236786 |
| 27 | P35052 | Glypican-1 OS=Homo sapiens OX=9606 GN=GPC1 PE=1 SV=2 | 20 | 0.595782768 | 0.11709 | 0.00623 | -0.747141698 |
| 28 | Q8IZ52 | Chondroitin sulfate synthase 2 OS=Homo sapiens OX=9606 GN=CHPF PE=1 SV=2 | 1 | 0.596580001 | 0.13002 | 0.00843 | -0.74521248 |
| 29 | P17813 | Endoglin OS=Homo sapiens OX=9606 GN=ENG PE=1 SV=2 | 2 | 0.611450467 | 0.02216 | 5.1E-05 | -0.709692463 |
| 30 | Q9GZM7 | Tubulointerstitial nephritis antigen-like OS=Homo sapiens OX=9606 GN=TINAGL1 PE=1 SV=1 | 5 | 0.61260306 | 0.13569 | 0.01066 | -0.706975522 |
| 31 | P06756 | Integrin alpha-V OS=Homo sapiens OX=9606 GN=ITGAV PE=1 SV=2 | 5 | 0.613223697 | 0.07972 | 0.00232 | -0.705514647 |
| 32 | P14209 | CD99 antigen OS=Homo sapiens OX=9606 GN=CD99 PE=1 SV=1 | 2 | 0.620551838 | 0.15668 | 0.01679 | -0.688376364 |
| 33 | Q15582 | Transforming growth factor-beta-induced protein ig-h3 OS=Homo sapiens OX=9606 GN=TGFBI PE=1 SV=1 | 28 | 0.623145849 | 0.06614 | 0.00145 | -0.682358224 |
| 34 | Q6ZRP7 | Sulfhydryl oxidase 2 OS=Homo sapiens OX=9606 GN=QSOX2 PE=1 SV=3 | 1 | 0.623900951 | 0.17804 | 0.02425 | -0.680611087 |
| 35 | P98160 | Basement membrane-specific heparan sulfate proteoglycan core protein OS=Homo sapiens OX=9606 GN=HSPG2 PE=1 SV=4 | 125 | 0.6265483 | 0.21214 | 0.03889 | -0.674502364 |
| 36 | O00468-6 | Isoform 6 of Agrin OS=Homo sapiens OX=9606 GN=AGRN | 73 | 0.627875148 | 0.19031 | 0.02971 | -0.671450385 |
| 37 | P34741 | Syndecan-2 OS=Homo sapiens OX=9606 GN=SDC2 PE=1 SV=2 | 1 | 0.630147955 | 0.15549 | 0.01763 | -0.66623749 |
| 38 | P05556 | Integrin beta-1 OS=Homo sapiens OX=9606 GN=ITGB1 PE=1 SV=2 | 11 | 0.637112713 | 0.09443 | 0.00458 | -0.65037947 |
| 39 | P55268 | Laminin subunit beta-2 OS=Homo sapiens OX=9606 GN=LAMB2 PE=1 SV=2 | 33 | 0.637278137 | 0.08916 | 0.00388 | -0.650004927 |
| 40 | Q99985 | Semaphorin-3C OS=Homo sapiens OX=9606 GN=SEMA3C PE=2 SV=2 | 5 | 0.640573567 | 0.11688 | 0.00865 | -0.642563828 |
| 41 | P11233 | Ras-related protein Ral-A OS=Homo sapiens OX=9606 GN=RALA PE=1 SV=1 | 3 | 0.645044465 | 0.18972 | 0.0333 | -0.63252948 |
| 42 | Q9BSQ5-2 | Isoform 2 of Cerebral cavernous malformations 2 protein OS=Homo sapiens OX=9606 GN=CCM2 | 1 | 0.649208425 | 0.07892 | 0.003 | -0.623246372 |
| 43 | P19021-5 | Isoform 5 of Peptidyl-glycine alpha-amidating monooxygenase OS=Homo sapiens OX=9606 GN=PAM | 2 | 0.649507827 | 0.1168 | 0.00927 | -0.622581183 |
| 44 | Q06481 | Amyloid-like protein 2 OS=Homo sapiens OX=9606 GN=APLP2 PE=1 SV=2 | 6 | 0.650406848 | 0.04636 | 0.00063 | -0.620585648 |
| 45 | P29966 | Myristoylated alanine-rich C-kinase substrate OS=Homo sapiens OX=9606 GN=MARCKS PE=1 SV=4 | 3 | 0.650905941 | 0.08792 | 0.00416 | -0.619479013 |
| 46 | Q8WUA8 | Tsukushin OS=Homo sapiens OX=9606 GN=TSKU PE=2 SV=3 | 2 | 0.655306105 | 0.16532 | 0.0251 | -0.609759123 |
| 47 | P20645 | Cation-dependent mannose-6-phosphate receptor OS=Homo sapiens OX=9606 GN=M6PR PE=1 SV=1 | 2 | 0.659334027 | 0.13881 | 0.01619 | -0.600918557 |
| 48 | P20020-1 | Isoform D of Plasma membrane calcium-transporting ATPase 1 OS=Homo sapiens OX=9606 GN=ATP2B1 | 1 | 0.66080781 | 0.1283 | 0.0132 | -0.597697357 |
| 49 | Q99816 | Tumor susceptibility gene 101 protein OS=Homo sapiens OX=9606 GN=TSG101 PE=1 SV=2 | 1 | 0.660957306 | 0.06815 | 0.00216 | -0.597371011 |
| 50 | P98088 | Mucin-5AC OS=Homo sapiens OX=9606 GN=MUC5AC PE=1 SV=4 | 3 | 0.661810456 | 0.18634 | 0.036 | -0.595510009 |
| 51 | Q9H4F8-2 | Isoform 2 of SPARC-related modular calcium-binding protein 1 OS=Homo sapiens OX=9606 GN=SMOC1 | 6 | 0.664050814 | 0.13482 | 0.01553 | -0.590634451 |
| 52 | O43570 | Carbonic anhydrase 12 OS=Homo sapiens OX=9606 GN=CA12 PE=1 SV=1 | 1 | 0.664401429 | 0.11086 | 0.00904 | -0.589872918 |
| 53 | P02452 | Collagen alpha-1(I) chain OS=Homo sapiens OX=9606 GN=COL1A1 PE=1 SV=5 | 25 | 0.665979716 | 0.14577 | 0.01951 | -0.586449858 |
| 54 | P62847-4 | Isoform 4 of 40S ribosomal protein S24 OS=Homo sapiens OX=9606 GN=RPS24 | 2 | 0.666542862 | 0.20847 | 0.04937 | -0.585230443 |
| 55 | O95297 | Myelin protein zero-like protein 1 OS=Homo sapiens OX=9606 GN=MPZL1 PE=1 SV=1 | 2 | 0.670648819 | 0.11053 | 0.00945 | -0.57637059 |
| 56 | Q13421-2 | Isoform 3 of Mesothelin OS=Homo sapiens OX=9606 GN=MSLN | 5 | 0.671334272 | 0.12162 | 0.01242 | -0.5748968 |
| 57 | P43121 | Cell surface glycoprotein MUC18 OS=Homo sapiens OX=9606 GN=MCAM PE=1 SV=2 | 3 | 0.673136662 | 0.10806 | 0.00906 | -0.57102866 |
| 58 | Q02388 | Collagen alpha-1(VII) chain OS=Homo sapiens OX=9606 GN=COL7A1 PE=1 SV=2 | 43 | 0.677737477 | 0.07843 | 0.00377 | -0.561201544 |
| 59 | P78333 | Glypican-5 OS=Homo sapiens OX=9606 GN=GPC5 PE=2 SV=1 | 5 | 0.684160007 | 0.0917 | 0.00627 | -0.547594321 |
| 60 | P11047 | Laminin subunit gamma-1 OS=Homo sapiens OX=9606 GN=LAMC1 PE=1 SV=3 | 42 | 0.687512296 | 0.14323 | 0.02225 | -0.540542578 |
| 61 | Q9Y653 | Adhesion G-protein coupled receptor G1 OS=Homo sapiens OX=9606 GN=ADGRG1 PE=1 SV=2 | 1 | 0.688049102 | 0.14092 | 0.02141 | -0.539416569 |
| 62 | B1AHL2 | Fibulin-1 OS=Homo sapiens OX=9606 GN=FBLN1 PE=1 SV=1 | 14 | 0.698136684 | 0.07714 | 0.00434 | -0.518418573 |
| 63 | P48960 | CD97 antigen OS=Homo sapiens OX=9606 GN=CD97 PE=1 SV=4 | 2 | 0.699496756 | 0.15424 | 0.02999 | -0.515610727 |
| 64 | Q32P28-3 | Isoform 3 of Prolyl 3-hydroxylase 1 OS=Homo sapiens OX=9606 GN=P3H1 | 2 | 0.704774859 | 0.15887 | 0.03389 | -0.504765633 |
| 65 | Q5JWF2 | Guanine nucleotide-binding protein G(s) subunit alpha isoforms XLas OS=Homo sapiens OX=9606 GN=GNAS PE=1 SV=2 | 4 | 0.704831833 | 0.15512 | 0.03188 | -0.504649012 |
| 66 | P20908-2 | Isoform 2 of Collagen alpha-1(V) chain OS=Homo sapiens OX=9606 GN=COL5A1 | 35 | 0.706094475 | 0.08396 | 0.00598 | -0.502066866 |
| 67 | P16070 | CD44 antigen OS=Homo sapiens OX=9606 GN=CD44 PE=1 SV=3 | 5 | 0.707174038 | 0.09201 | 0.00785 | -0.499862783 |
| 68 | P30479 | HLA class I histocompatibility antigen, B-41 alpha chain OS=Homo sapiens OX=9606 GN=HLA-B PE=1 SV=1 | 6 | 0.707990998 | 0.05183 | 0.0015 | -0.498197078 |
| 69 | Q8NG11 | Tetraspanin-14 OS=Homo sapiens OX=9606 GN=TSPAN14 PE=1 SV=1 | 2 | 0.712050662 | 0.07355 | 0.00434 | -0.489948203 |
| 70 | P63218 | Guanine nucleotide-binding protein G(I)/G(S)/G(O) subunit gamma-5 OS=Homo sapiens OX=9606 GN=GNG5 PE=1 SV=3 | 1 | 0.712153436 | 0.09377 | 0.00869 | -0.489739986 |
| 71 | Q8WXI7 | Mucin-16 OS=Homo sapiens OX=9606 GN=MUC16 PE=1 SV=3 | 34 | 0.718562916 | 0.11611 | 0.01675 | -0.476813613 |
| 72 | G3XAI2 | Laminin subunit beta-1 OS=Homo sapiens OX=9606 GN=LAMB1 PE=1 SV=1 | 45 | 0.719476373 | 0.16131 | 0.04011 | -0.474980784 |
| 73 | O60701 | UDP-glucose 6-dehydrogenase OS=Homo sapiens OX=9606 GN=UGDH PE=1 SV=1 | 2 | 0.721878388 | 0.07748 | 0.00557 | -0.470172283 |
| 74 | P01891 | HLA class I histocompatibility antigen, A-68 alpha chain OS=Homo sapiens OX=9606 GN=HLA-A PE=1 SV=4 | 4 | 0.723546132 | 0.13829 | 0.02804 | -0.466843091 |
| 75 | A0A0A0MQS9 | Laminin subunit alpha-4 OS=Homo sapiens OX=9606 GN=LAMA4 PE=1 SV=1 | 56 | 0.72401404 | 0.16839 | 0.0465 | -0.46591042 |
| 76 | P39019 | 40S ribosomal protein S19 OS=Homo sapiens OX=9606 GN=RPS19 PE=1 SV=2 | 4 | 0.725313358 | 0.16061 | 0.04183 | -0.463323676 |
| 77 | P25391 | Laminin subunit alpha-1 OS=Homo sapiens OX=9606 GN=LAMA1 PE=1 SV=2 | 22 | 0.725576304 | 0.09006 | 0.00888 | -0.462800755 |
| 78 | Q6WRI0 | Immunoglobulin superfamily member 10 OS=Homo sapiens OX=9606 GN=IGSF10 PE=1 SV=1 | 22 | 0.727209617 | 0.11009 | 0.01577 | -0.459556817 |
| 79 | Q9P2B2 | Prostaglandin F2 receptor negative regulator OS=Homo sapiens OX=9606 GN=PTGFRN PE=1 SV=2 | 13 | 0.728211915 | 0.13111 | 0.02549 | -0.457569749 |
| 80 | P08962 | CD63 antigen OS=Homo sapiens OX=9606 GN=CD63 PE=1 SV=2 | 1 | 0.733118165 | 0.11807 | 0.02024 | -0.447882342 |
| 81 | K7ELC2 | 40S ribosomal protein S15 OS=Homo sapiens OX=9606 GN=RPS15 PE=1 SV=1 | 1 | 0.742581393 | 0.11317 | 0.0199 | -0.429378929 |
| 82 | P08174-7 | Isoform 7 of Complement decay-accelerating factor OS=Homo sapiens OX=9606 GN=CD55 | 2 | 0.743032095 | 0.14528 | 0.03843 | -0.428503565 |
| 83 | P12931-2 | Isoform 2 of Proto-oncogene tyrosine-protein kinase Src OS=Homo sapiens OX=9606 GN=SRC | 2 | 0.743974323 | 0.05893 | 0.00321 | -0.426675265 |
| 84 | P36405 | ADP-ribosylation factor-like protein 3 OS=Homo sapiens OX=9606 GN=ARL3 PE=1 SV=2 | 1 | 0.75028617 | 0.15672 | 0.04984 | -0.41448713 |
| 85 | P46934-4 | Isoform 4 of E3 ubiquitin-protein ligase NEDD4 OS=Homo sapiens OX=9606 GN=NEDD4 | 2 | 0.752046337 | 0.14892 | 0.04472 | -0.411106538 |
| 86 | Q6BBL7 | Olfactomedin-like protein 2A OS=Homo sapiens OX=9606 GN=OLFML2A PE=2 SV=1 | 2 | 0.75231942 | 0.12366 | 0.0279 | -0.410582763 |
| 87 | P19827 | Inter-alpha-trypsin inhibitor heavy chain H1 OS=Homo sapiens OX=9606 GN=ITIH1 PE=1 SV=3 | 3 | 0.752926803 | 0.04895 | 0.00207 | -0.409418478 |
| 88 | P16144 | Integrin beta-4 OS=Homo sapiens OX=9606 GN=ITGB4 PE=1 SV=5 | 2 | 0.753491309 | 0.1236 | 0.02821 | -0.408337223 |
| 89 | O75787 | Renin receptor OS=Homo sapiens OX=9606 GN=ATP6AP2 PE=1 SV=2 | 4 | 0.760666 | 0.11986 | 0.02813 | -0.394664973 |

| | | | | | | | |
|---|---|---|---|---|---|---|---|
| 90 | P46777 | 60S ribosomal protein L5 OS=Homo sapiens OX=9606 GN=RPL5 PE=1 SV=3 | 2 | 0.76281197 | 0.13616 | 0.03994 | -0.390600613 |
| 91 | P43034 | Platelet-activating factor acetylhydrolase IB subunit alpha OS=Homo sapiens OX=9606 GN=PAFAH1B1 PE=1 SV=2 | 1 | 0.76627447 | 0.08561 | 0.01207 | -0.384066856 |
| 92 | O94985 | Calsyntenin-1 OS=Homo sapiens OX=9606 GN=CLSTN1 PE=1 SV=1 | 6 | 0.770017393 | 0.13546 | 0.0426 | -0.377037062 |
| 93 | P04899-4 | Isoform sGi2 of Guanine nucleotide-binding protein G(i) subunit alpha-2 OS=Homo sapiens OX=9606 GN=GNAI2 | 8 | 0.771613071 | 0.09986 | 0.01961 | -0.374050512 |
| 94 | P62879 | Guanine nucleotide-binding protein G(I)/G(S)/G(T) subunit beta-2 OS=Homo sapiens OX=9606 GN=GNB2 PE=1 SV=3 | 5 | 0.771696624 | 0.05831 | 0.00434 | -0.373894301 |
| 95 | Q92954-5 | Isoform E of Proteoglycan 4 OS=Homo sapiens OX=9606 GN=PRG4 | 6 | 0.773149783 | 0.11192 | 0.02705 | -0.371180159 |
| 96 | P35241-5 | Isoform 5 of Radixin OS=Homo sapiens OX=9606 GN=RDX | 10 | 0.777483563 | 0.04328 | 0.00196 | -0.36311592 |
| 97 | Q02818 | Nucleobindin-1 OS=Homo sapiens OX=9606 GN=NUCB1 PE=1 SV=4 | 1 | 0.778584058 | 0.10919 | 0.02701 | -0.361075289 |
| 98 | Q9Y3L5 | Ras-related protein Rap-2c OS=Homo sapiens OX=9606 GN=RAP2C PE=1 SV=1 | 2 | 0.778872372 | 0.10293 | 0.02319 | -0.360541151 |
| 99 | Q99733-2 | Isoform 2 of Nucleosome assembly protein 1-like 4 OS=Homo sapiens OX=9606 GN=NAP1L4 | 7 | 0.784429376 | 0.01337 | 6.5E-05 | -0.350284531 |
| 100 | Q6UVK1 | Chondroitin sulfate proteoglycan 4 OS=Homo sapiens OX=9606 GN=CSPG4 PE=1 SV=2 | 6 | 0.785139117 | 0.12732 | 0.04325 | -0.34897979 |
| 101 | Q9BXJ4-3 | Isoform 3 of Complement C1q tumor necrosis factor-related protein 3 OS=Homo sapiens OX=9606 GN=C1QTNF3 | 1 | 0.789962225 | 0.04181 | 0.0021 | -0.340144429 |
| 102 | O95372 | Acyl-protein thioesterase 2 OS=Homo sapiens OX=9606 GN=LYPLA2 PE=1 SV=1 | 1 | 0.791413257 | 0.10344 | 0.02741 | -0.337496862 |
| 103 | P62316 | Small nuclear ribonucleoprotein Sm D2 OS=Homo sapiens OX=9606 GN=SNRPD2 PE=1 SV=1 | 2 | 0.79769375 | 0.04614 | 0.00312 | -0.326093121 |
| 104 | P08123 | Collagen alpha-2(I) chain OS=Homo sapiens OX=9606 GN=COL1A2 PE=1 SV=7 | 1 | 0.808332221 | 0.10325 | 0.03397 | -0.306979738 |
| 105 | P05023 | Sodium/potassium-transporting ATPase subunit alpha-1 OS=Homo sapiens OX=9606 GN=ATP1A1 PE=1 SV=1 | 13 | 0.812509647 | 0.08551 | 0.02196 | -0.299543152 |
| 106 | P06280 | Alpha-galactosidase A OS=Homo sapiens OX=9606 GN=GLA PE=1 SV=1 | 1 | 0.81720074 | 0.10174 | 0.03693 | -0.291237584 |
| 107 | P05121 | Plasminogen activator inhibitor 1 OS=Homo sapiens OX=9606 GN=SERPINE1 PE=1 SV=1 | 7 | 0.817580094 | 0.08039 | 0.02003 | -0.290568024 |
| 108 | P63000-2 | Isoform B of Ras-related C3 botulinum toxin substrate 1 OS=Homo sapiens OX=9606 GN=RAC1 | 6 | 0.817852276 | 0.02697 | 0.00088 | -0.290087813 |
| 109 | Q15113 | Procollagen C-endopeptidase enhancer 1 OS=Homo sapiens OX=9606 GN=PCOLCE PE=1 SV=2 | 1 | 0.819369052 | 0.08464 | 0.0236 | -0.287414693 |
| 110 | P61586 | Transforming protein RhoA OS=Homo sapiens OX=9606 GN=RHOA PE=1 SV=1 | 6 | 0.819800213 | 0.07842 | 0.01936 | -0.286655731 |
| 111 | Q3ZCW2 | Galectin-related protein OS=Homo sapiens OX=9606 GN=LGALSL PE=1 SV=2 | 1 | 0.820513224 | 0.02983 | 0.00123 | -0.285401509 |
| 112 | P06858 | Lipoprotein lipase OS=Homo sapiens OX=9606 GN=LPL PE=1 SV=1 | 4 | 0.827198501 | 0.05682 | 0.00893 | -0.273694524 |
| 113 | P54289 | Voltage-dependent calcium channel subunit alpha-2/delta-1 OS=Homo sapiens OX=9606 GN=CACNA2D1 PE=1 SV=3 | 3 | 0.827833978 | 0.10169 | 0.0429 | -0.27258663 |
| 114 | A0A0A0MS09 | Immunoglobulin heavy constant delta (Fragment) OS=Homo sapiens OX=9606 GN=IGHD PE=1 SV=1 | 4 | 0.831879983 | 0.06821 | 0.01601 | -0.265552692 |
| 115 | P41221 | Protein Wnt-5a OS=Homo sapiens OX=9606 GN=WNT5A PE=1 SV=2 | 8 | 0.831992083 | 0.05298 | 0.00793 | -0.265358295 |
| 116 | P50995 | Annexin A11 OS=Homo sapiens OX=9606 GN=ANXA11 PE=1 SV=1 | 6 | 0.833369415 | 0.06574 | 0.01482 | -0.262971941 |
| 117 | P01023 | Alpha-2-macroglobulin OS=Homo sapiens OX=9606 GN=A2M PE=1 SV=3 | 11 | 0.836418957 | 0.04971 | 0.00714 | -0.257702336 |
| 118 | P48509 | CD151 antigen OS=Homo sapiens OX=9606 GN=CD151 PE=1 SV=3 | 1 | 0.838845013 | 0.05819 | 0.0116 | -0.253523815 |
| 119 | Q15435 | Protein phosphatase 1 regulatory subunit 7 OS=Homo sapiens OX=9606 GN=PPP1R7 PE=1 SV=1 | 1 | 0.840477604 | 0.07351 | 0.02258 | -0.250718718 |
| 120 | P61247 | 40S ribosomal protein S3a OS=Homo sapiens OX=9606 GN=RPS3A PE=1 SV=2 | 6 | 0.84535667 | 0.06624 | 0.01855 | -0.242367928 |
| 121 | P62263 | 40S ribosomal protein S14 OS=Homo sapiens OX=9606 GN=RPS14 PE=1 SV=3 | 3 | 0.848900644 | 0.07216 | 0.02482 | -0.236332385 |
| 122 | P55209 | Nucleosome assembly protein 1-like 1 OS=Homo sapiens OX=9606 GN=NAP1L1 PE=1 SV=1 | 8 | 0.854808257 | 0.04223 | 0.0063 | -0.22632725 |
| 123 | P02790 | Hemopexin OS=Homo sapiens OX=9606 GN=HPX PE=1 SV=2 | 21 | 0.858651959 | 0.07391 | 0.03147 | -0.219854618 |
| 124 | Q07954 | Prolow-density lipoprotein receptor-related protein 1 OS=Homo sapiens OX=9606 GN=LRP1 PE=1 SV=2 | 26 | 0.863676922 | 0.03693 | 0.00514 | -0.211436354 |
| 125 | Q6V0I7 | Protocadherin Fat 4 OS=Homo sapiens OX=9606 GN=FAT4 PE=1 SV=2 | 6 | 0.868539102 | 0.02876 | 0.00277 | -0.203337293 |
| 126 | Q12906-7 | Isoform 7 of Interleukin enhancer-binding factor 3 OS=Homo sapiens OX=9606 GN=ILF3 | 10 | 0.876239125 | 0.05983 | 0.02563 | -0.190603461 |
| 127 | P19075 | Tetraspanin-8 OS=Homo sapiens OX=9606 GN=TSPAN8 PE=1 SV=1 | 1 | 0.881550838 | 0.06043 | 0.02952 | -0.181884324 |
| 128 | P24821 | Tenascin OS=Homo sapiens OX=9606 GN=TNC PE=1 SV=3 | 5 | 0.881790084 | 0.04638 | 0.0146 | -0.181492841 |
| 129 | Q14697-2 | Isoform 2 of Neutral alpha-glucosidase AB OS=Homo sapiens OX=9606 GN=GANAB | 8 | 0.889887468 | 0.06473 | 0.0424 | -0.168305186 |
| 130 | X6R8A1 | Carboxypeptidase OS=Homo sapiens OX=9606 GN=CTSA PE=1 SV=1 | 1 | 0.917376574 | 0.03541 | 0.01858 | -0.124414028 |
| 131 | P09382 | Galectin-1 OS=Homo sapiens OX=9606 GN=LGALS1 PE=1 SV=2 | 6 | 0.918720874 | 0.04597 | 0.03847 | -0.122301488 |
| 132 | P62820 | Ras-related protein Rab-1A OS=Homo sapiens OX=9606 GN=RAB1A PE=1 SV=3 | 7 | 0.92221748 | 0.03257 | 0.01745 | -0.116821084 |
| 133 | E9PGN7 | Plasma protease C1 inhibitor OS=Homo sapiens OX=9606 GN=SERPING1 PE=1 SV=1 | 1 | 0.923893954 | 0.03741 | 0.02678 | -0.114200829 |
| 134 | P63104 | 14-3-3 protein zeta/delta OS=Homo sapiens OX=9606 GN=YWHAZ PE=1 SV=1 | 10 | 0.936113582 | 0.03936 | 0.04762 | -0.095244507 |
| 135 | P61224 | Ras-related protein Rap-1b OS=Homo sapiens OX=9606 GN=RAP1B PE=1 SV=1 | 9 | 0.941186551 | 0.03462 | 0.04255 | -0.08744739 |
| 136 | P24043 | Laminin subunit alpha-2 OS=Homo sapiens OX=9606 GN=LAMA2 PE=1 SV=4 | 3 | 0.944338322 | 0.03307 | 0.04352 | -0.082624278 |

## Appendix Table S2 List of proteins increased or decreased in HeLa cell Rab11a-exosome preparations, related to Figure 2

Tables show lists of HeLa cell proteins with statistically significant increased or decreased levels in Rab11a-exosome-enriched sEV preparations versus sEV preparations collected under normal glutamine-replete (2 mM) culture conditions and both concentrated by size-exclusion chromatography. Secretion of Rab11a-exosomes was induced by culturing in 0.02 mM glutamine. Data were generated from four pair-wise comparisons using the TMT labelling approach, so that all samples were analysed in the same MS run. Orange highlighted accession numbers denote those proteins that were similarly increased or decreased in a previous comparative TMT proteomics analysis of HCT116 sEVs collected under glutamine-depleted versus -replete conditions and concentrated by ultracentrifugation (Marie et al, 2023). Green highlighting denotes the two glycolytic enzymes, ALDOA and PKM. GAPDH levels were increased in all four Rab11a-exosome-enriched sEV preparations, but the

change compared to control sEVs narrowly failed to reach significance. Note APP and APLP2 are both reduced in Rab11a-exosome-enriched preparations; their ICDs would be expected to be present in late endosomal exosomes as well as Rab11a-exosomes, and in SCs, we observe accumulation of the APPL ICD at the surface of and inside late endosomal and lysosomal compartments, as well as in Rab11-compartments.

## Appendix Figures

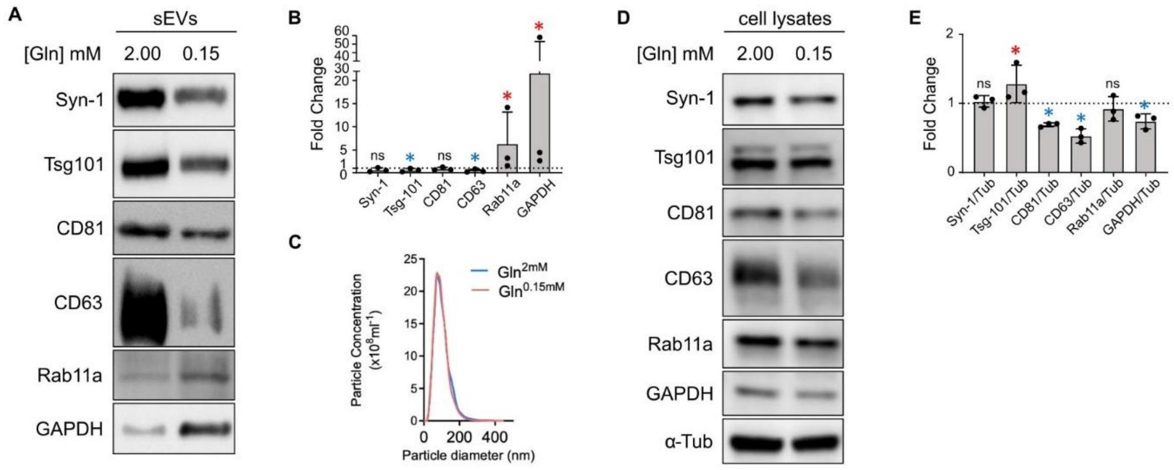


**Appendix Figure S1 GAPDH is enriched in human HCT116 Rab11a-exosome preparations, related to Figure 2**

(A and B) Western blot analysis of putative exosome markers in sEV preparations concentrated by ultracentrifugation and collected from HCT116 cells cultured under glutamine-replete (2.00 mM) and glutamine-depleted (0.15 mM) conditions for 24 h (A). Loading is based on cell lysate protein levels, so that secretion is compared on a per cell basis. Syn-1 = Syntenin-1. Bar charts show relative change in exosome protein levels in sEVs collected under glutamine-depleted versus -replete conditions, ie. Rab11a-exosome-enriched versus -depleted sEV preparations respectively (B).

(C) Nanosight Tracking Analysis of EV size and number for diluted sEV samples (normalised to cell lysate protein levels) from cells cultured in glutamine-replete and glutamine-depleted conditions for 24 h, as in Figure S1A.

(D and E) Western blot analysis of putative exosome proteins in lysates from HCT116 cells cultured under glutamine-replete (2.00 mM) and glutamine-depleted (0.15 mM) conditions for 24 h (D). Equal amounts of protein were loaded. Bar chart shows relative abundance of putative exosome proteins in these lysates, normalised to relative

abundance of tubulin (E). The elevated levels of Rab11a and GAPDH in Rab11a-exosome-enriched sEV preparations cannot be explained by increased cellular expression.

For bar charts, data are mean ± SEM, analysed using the Kruskal-Wallis test; *$P<0.05$, ns = not significant.

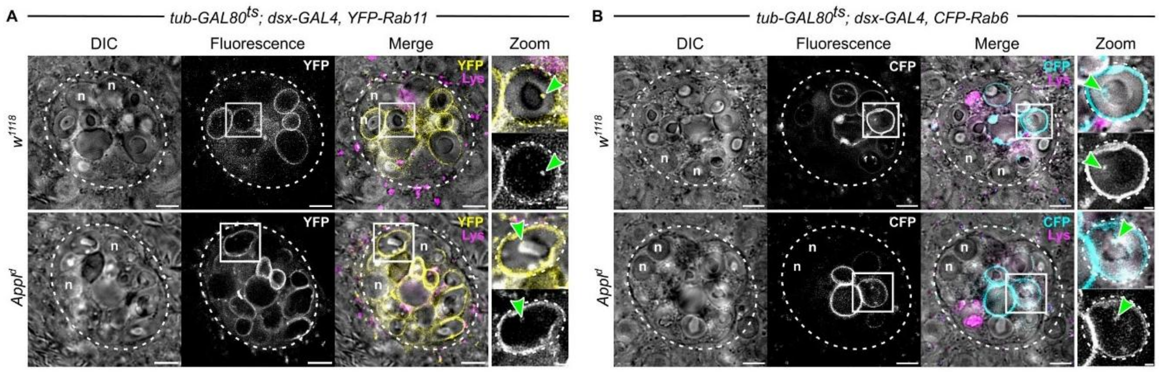


**Appendix Figure S2 DCG compartments in *Appl*$^{d}$ mutant SCs carry Rab11 and Rab6 markers**

(A and B) *Ex vivo*, wide-field fluorescence micrographs and DIC images of SCs from 6-day-old wild type and *Appl*$^{d}$ mutant males expressing *YFP-Rab11* (A) or *CFP-Rab6* (B) from the endogenous *Rab* gene loci. All non-acidic compartments containing DCGs are Rab11-positive and about half are Rab6-positive. Rab-positive ILVs (green arrowheads) in compartments are labelled in Zoom panels. In all images, n = nuclei; LysoTracker Red (magenta) marks acidic compartments. Scale bars = 5 µm and 1 µm in Zoom.

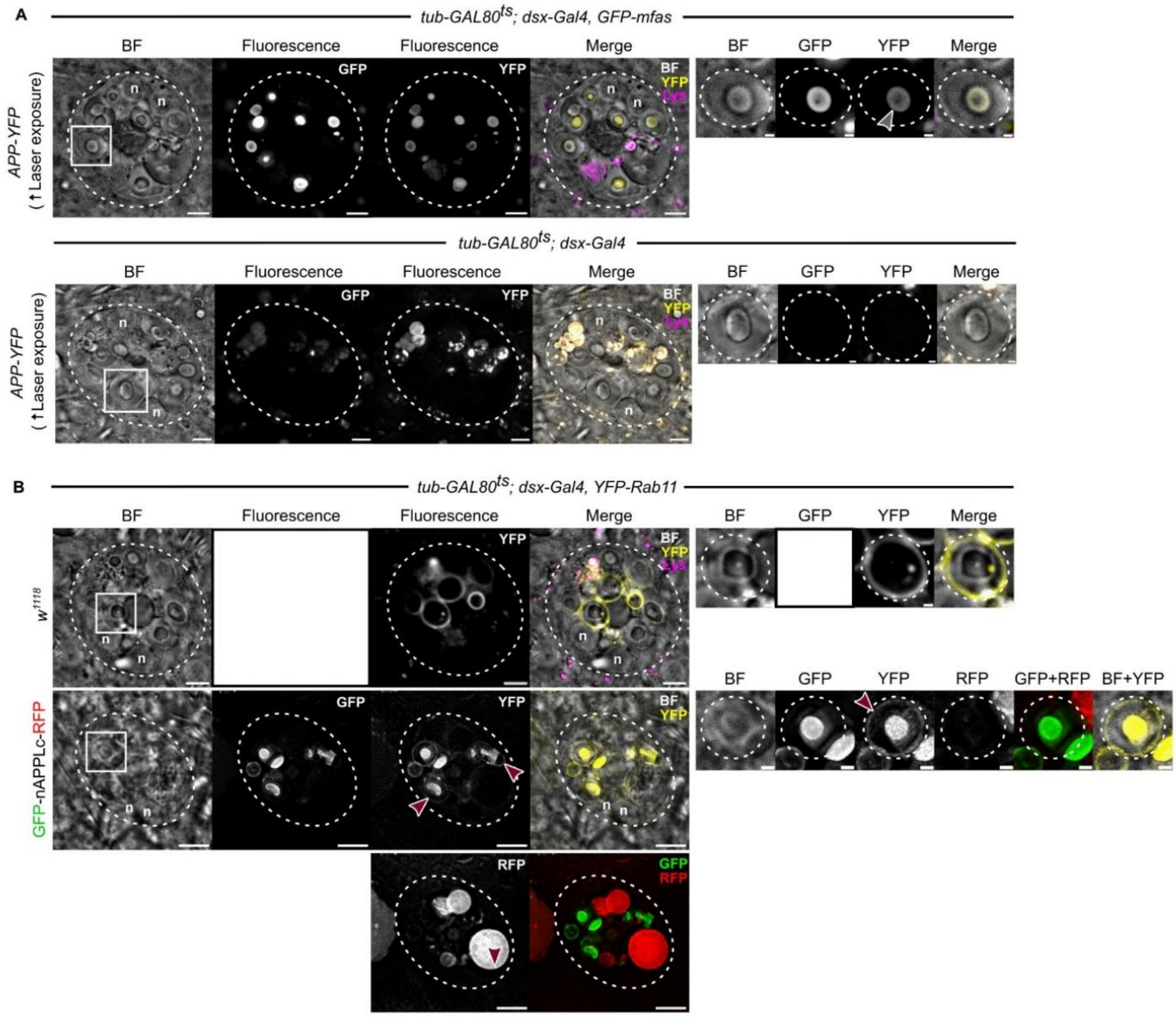


**Appendix Figure S3 – Overexpression of fluorescently tagged APP and APPL does not severely disrupt DCG compartment identity and morphology in SCs**

(A) SCs expressing APP-YFP either in the presence of *GFP-mfas* gene trap or in its absence. At normal laser gain and exposure settings, no signal is detected in the YFP channel in either genotype. When the exposure time is increased, the DCGs are detected in GFP-MFAS-expressing SCs in the YFP channel, as well as the strongest signals from LysoTracker Red staining. However, using the same more sensitive settings to image SCs expressing APP-YFP alone, only the LysoTracker Red signal is observed in the YFP channel, and no signal in DCG compartments (see Zoom

images). Therefore, APP-YFP fluorescence does not contribute to the fluorescence signals observed in the rescue experiments in Figure 3B.

(B) SCs expressing YFP-Rab11 from the endogenous *Rab11* locus either in the absence or presence of dt-APPL and imaged using settings optimised to detect GFP, YFP and mRFP. Note that, as expected, mature DCG compartment membranes are detected in the YFP channel in the absence of dt-APPL. In the presence of dt-APPL, the GFP-tagged extracellular domain of APPL is detected within DCGs in the GFP and YFP channels (see Zoom images), but the limiting membranes of mature DCG compartments is most clearly detected in the YFP channel. Therefore, these mature DCG compartments have Rab11 identity.

Images collected on the Leica Thunder, also using brightfield (BF) setting. GFP bleed-through into YFP channel in (A) marked with grey arrowhead. YFP-Rab11 marked limiting membrane of DCG compartments in (B) marked with brown arrowheads. In all images, n = nuclei; LysoTracker Red (magenta) marks acidic compartments, escept in dt-APPL-expressing SC. Scale bars = 5 µm and 1 µm in Zoom.

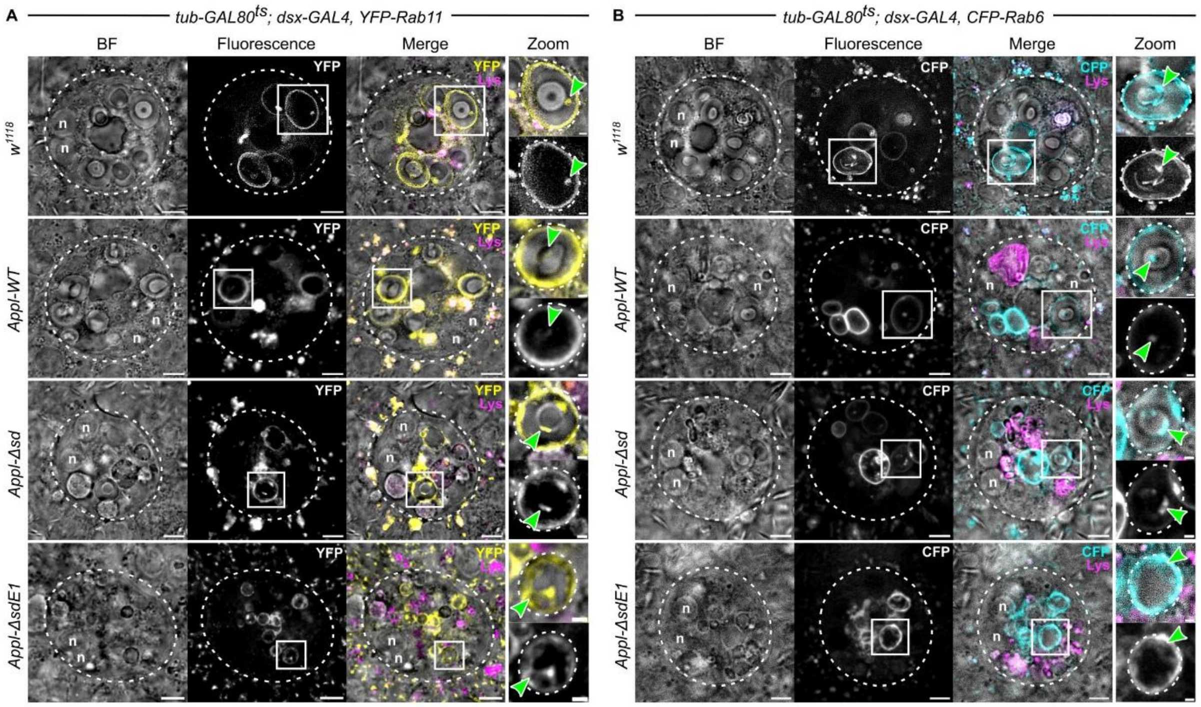


**Appendix Figure S4 – DCG compartments in SCs overexpressing wild type and mutant APPL constructs have expected Rab11/Rab6 identity**

(A) SCs expressing YFP-Rab11 alone or with APPL-WT, APPL-∆sd and APPL-∆sdE1 overexpression. Note large non-acidic compartments containing DCGs or other protein aggregates as determined by DIC are Rab11-positive. Also note the extensive network of peripheral YFP-Rab11 fluorescence when APPL-∆sdE1 is overexpressed and that the number of acidic compartments is increased in SCs overexpressing non-cleavable APPL (see Figure 5E).

(B) SCs expressing CFP-Rab6 alone or with APPL-WT, APPL-∆sd and APPL-∆sdE1 overexpression. Note about half of the compartments containing DCGs or other aggregates of GFP-MFAS are Rab6-positive, and the network of peripheral CFP-Rab6 fluorescence when APPL-∆sdE1 is overexpressed.

Rab-positive ILVs (green arrowheads) inside compartments are marked in Zoom panels. In all images, n = nuclei; LysoTracker Red (magenta) marks acidic compartments. Scale bars = 5 µm and 1 µm in Zoom.

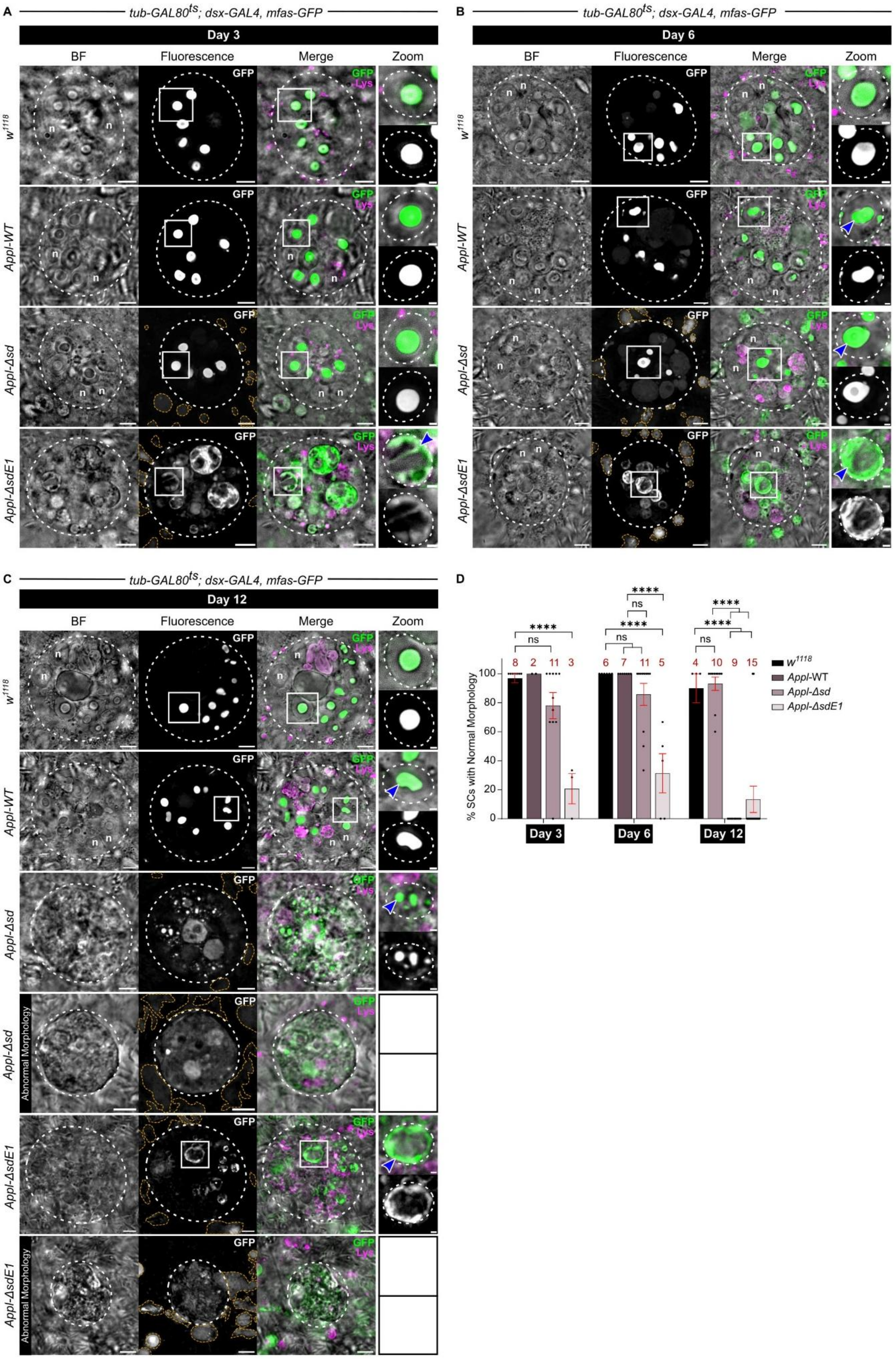
A
tub-GAL80ts; dsx-GAL4, mfas-GFP
Day 3
BF
Fluorescence
Merge
Zoom
GFP
Lys
w1118
Appl-WT
Appl-Δsd
Appl-ΔsdE1
B
tub-GAL80ts; dsx-GAL4, mfas-GFP
Day 6
C
tub-GAL80ts; dsx-GAL4, mfas-GFP
Day 12
Abnormal Morphology
D
% SCs with Normal Morphology
Day 3
Day 6
Day 12
ns
****
w1118
Appl-WT
Appl-Δsd
Appl-ΔsdE1

**Appendix Figure S5 SCs overexpressing mutant APPL constructs develop morphological abnormalities as adult males age**

(A-C) SCs expressing *GFP-mfas* gene trap alone or with overexpressed APPL-WT, APPL-∆sd and APPL-∆sdE1, dissected from 3- (A), 6- (B) and 12-day-old (C) males.

(D-F) Bar charts showing proportions of SCs with normal overall morphology in AGs at different time points for each genotype (abnormal SCs identified by characteristic GFP-MFAS accumulation in cells lacking obvious secretory compartments).

In all images, n = nuclei; LysoTracker Red (magenta) marks acidic compartments. Scale bars = 5 µm and 1 µm in Zoom. For bar charts, data are mean ± SEM, analysed using the Kruskal-Wallis test; n = animal number above bar, ****P<0.0001, ns = not significant.

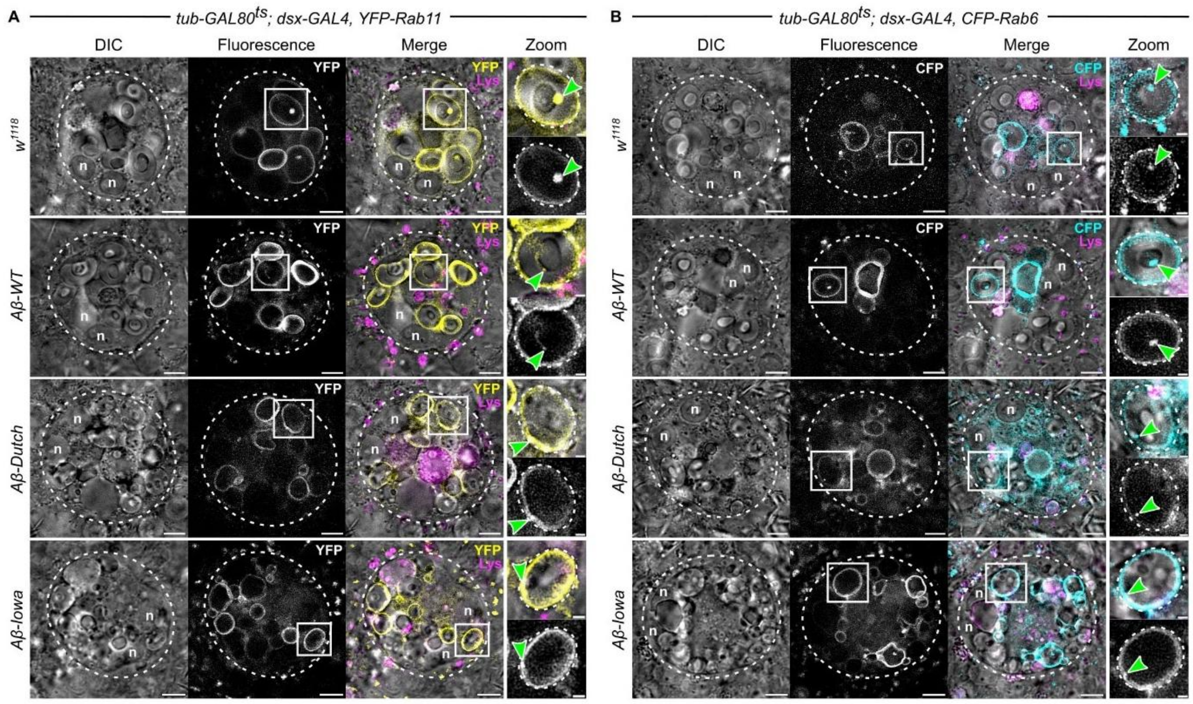


**Appendix Figure S6 DCG compartments in SCs overexpressing wild type and mutant Aβ-42 constructs have expected Rab11/Rab6 identity**

(A) SCs expressing YFP-Rab11 alone or with overexpressed wild type Aβ-42 peptide, or either the Iowa or Dutch mutant Aβ-42 peptides. Note all compartments containing DCGs or other protein aggregates as determined by DIC, including mini-cores, are Rab11-positive. Also note the increased number of acidic compartments when Aβ-peptides are overexpressed.

(B) SCs expressing CFP-Rab6 and overexpressing wild type Aβ-42 peptide, or either the Iowa or Dutch mutant Aβ-42 peptides. Note about half of the compartments containing aggregates of GFP-MFAS, including mini-cores, are Rab6-positive.

Rab-positive ILVs (green arrowheads) inside compartments, which are more peripherally located when mini-cores are generated, are marked in Zoom panels. In all images, n = nuclei; LysoTracker Red (magenta) marks acidic compartments. Scale bars = 5 µm and 1 µm in Zoom.

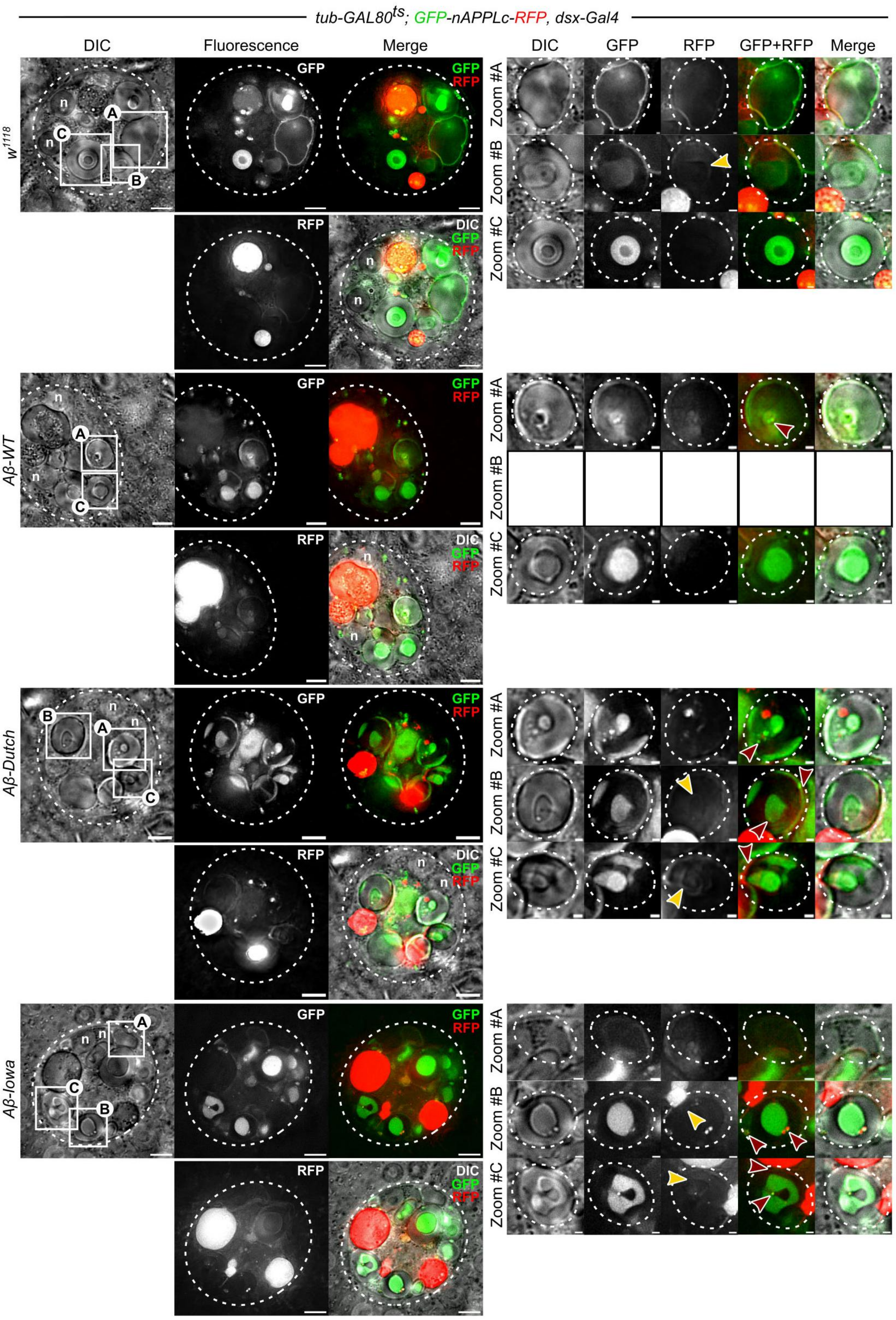
tub-GAL80ts; GFP-nAPPLc-RFP, dsx-Gal4
DIC
Fluorescence
Merge
DIC
GFP
RFP
GFP+RFP
Merge
w1118
Aβ-WT
Aβ-Dutch
Aβ-Iowa
Zoom #A
Zoom #B
Zoom #C
GFP
RFP
DIC
n
A
B
C

**Appendix Figure S7 Cleavage of APPL in DCG compartments is affected by overexpression of mutant Aβ-peptides**

SCs from 6-day-old males expressing either dt-APPL alone or in combination with wild type Aβ-42 peptide, or either the Iowa or Dutch mutant Aβ-42 peptides. For the different genotypes, magnified images in Zoom highlight a DCG precursor compartment with GFP and RFP fluorescence at the compartment limiting membrane and limited DCG condensation (Zoom A); a more mature DCG compartment with some DCG condensation (Zoom B; not seen in the Aβ-WT cell selected here) and a more mature DCG compartment, which has little or no peripheral APPL ICD and ECD at the compartment's limiting membrane in control cells (Zoom C). The ICD and ECD are observed along parts of the compartmental limiting membrane in Zoom C for Aβ-expressing SCs. Chains of ILVs at the surface of DCGs (yellow arrowheads) and individual punctate clusters marked by both the APPL ICD and ECD (brown arrowheads) are frequently observed in SCs expressing Aβ-mutant peptides. These findings suggest defects in APPL processing. Aβ Dutch also frequently induces crescent-shaped aggregates at the periphery of DCG compartments.

In all images, n = nuclei. Scale bars = 5 µm and 1 µm in Zoom.

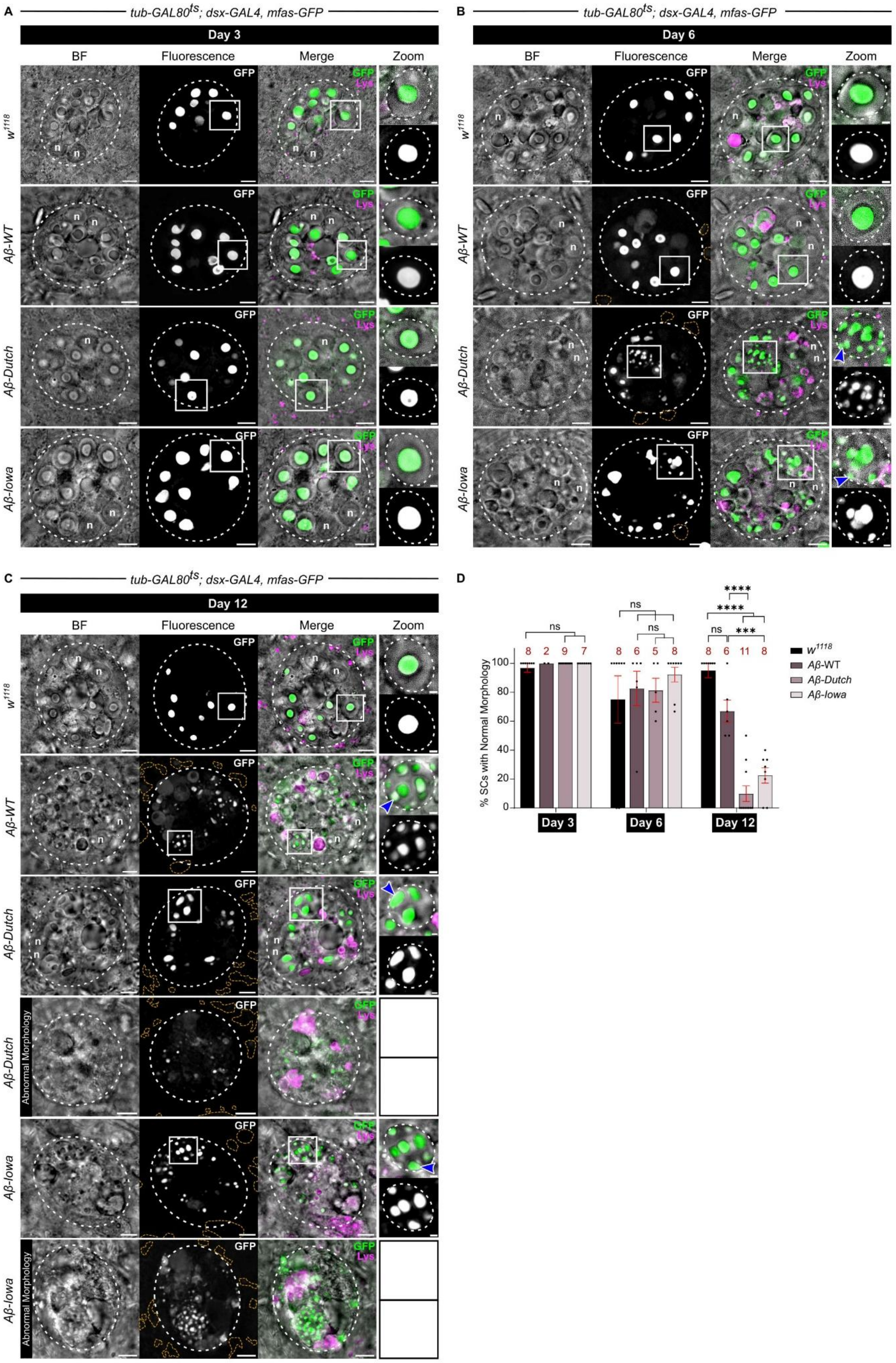
A
tub-GAL80ts; dsx-GAL4, mfas-GFP
Day 3
BF
Fluorescence
Merge
Zoom
GFP
Lys
w1118
Aβ-WT
Aβ-Dutch
Aβ-Iowa
B
tub-GAL80ts; dsx-GAL4, mfas-GFP
Day 6
C
tub-GAL80ts; dsx-GAL4, mfas-GFP
Day 12
Abnormal Morphology
D
% SCs with Normal Morphology
Day 3
Day 6
Day 12
ns
****
***
w1118
Aβ-WT
Aβ-Dutch
Aβ-Iowa

**Appendix Figure S8 SCs overexpressing mutant Aβ-42 constructs develop morphological abnormalities in 12-day-old adult males**

(A-C) SCs expressing *GFP-mfas* gene trap alone or with wild type Aβ-42 peptide, or either the Iowa or Dutch mutant Aβ-42 peptides, dissected from 3- (A), 6- (B) and 12-day-old (C) males. Note that SCs expressing wild type Aβ-42 begin to display the mini-core phenotype after 12 days.

(D-F) Bar charts showing proportions of SCs with normal overall morphology in AGs at different time points for each genotype (abnormal SCs identified by characteristic GFP-MFAS accumulation in cells lacking obvious secretory compartments).

In all images, n = nuclei; LysoTracker Red (magenta) marks acidic compartments. Scale bars = 5 µm and 1 µm in Zoom. For bar charts, data are mean ± SEM, analysed using the Kruskal-Wallis test; n = animal number above bar, ***$P<0.001$, ****$P<0.0001$, ns = not significant.

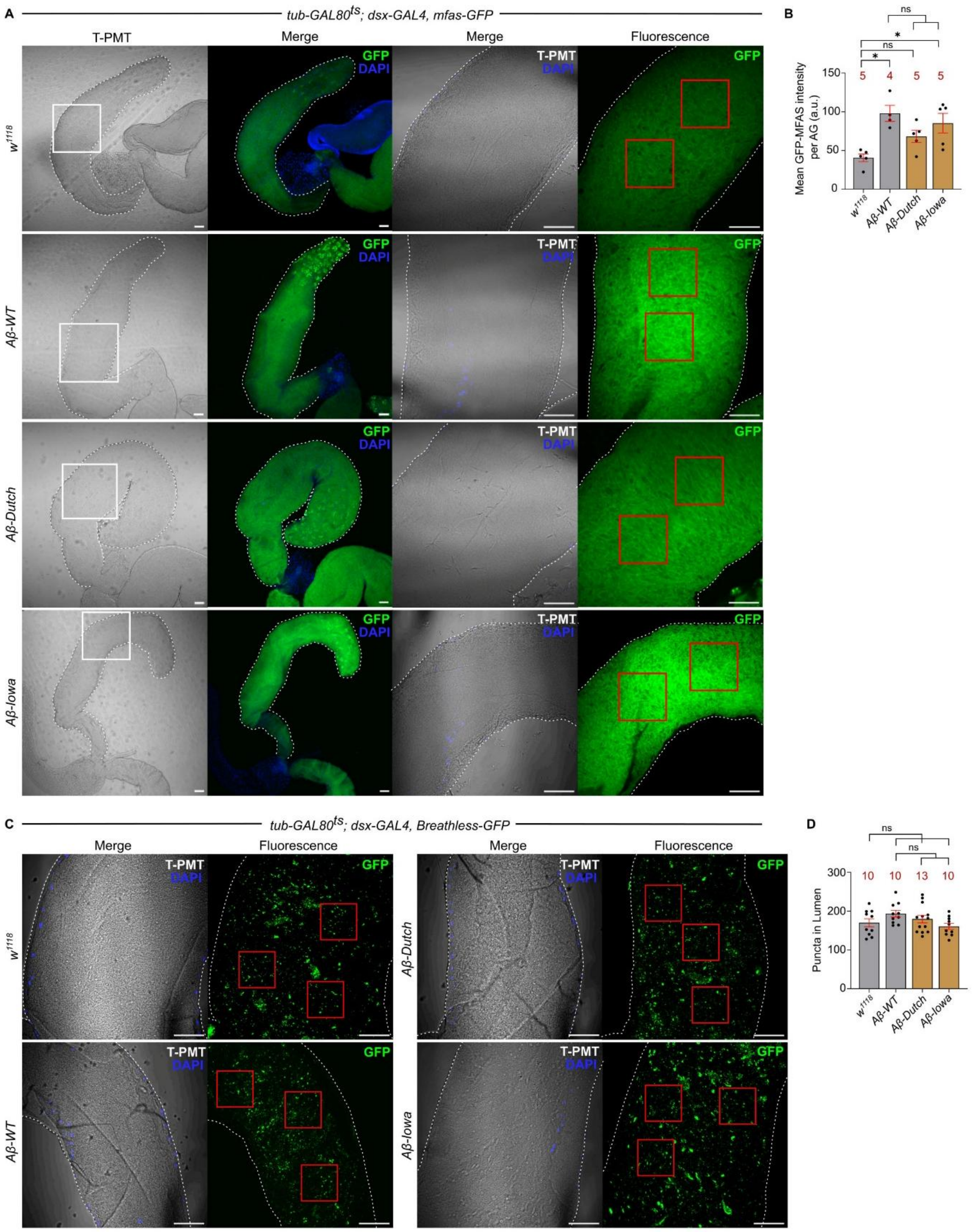


**Appendix Figure S9 Effects of pathological Aβ-peptide expression in SCs on GFP-MFAS and exosome-associated Btl-GFP secretion, related to Figure 6**

(A) Confocal micrographs (T-PMT [transmission detection module for confocal] and GFP/DAPI fluorescence) of fixed accessory glands of 6-day-old males expressing

*GFP-mfas* gene trap and either no other transgene, or wild type Aβ-42 peptide, or either the Iowa or Dutch mutant Aβ-42 peptides in SCs. Two panels on right-hand side are magnified images of region in white box in left-hand panel. Two red boxes on right-hand panels mark two lumenal regions from which GFP signal was measured in these specific images.

(B) Bar chart showing mean GFP intensity in accessory gland lumen for the four genotypes shown in (A).

(C) Confocal micrographs (DIC with DAPI and GFP fluorescence) of fixed accessory glands of 6-day-old males expressing *Breathless -GFP* (*Btl-GFP*) and either no other transgene, or wild type Aβ-42 peptide, or either the Iowa or Dutch mutant Aβ-42 peptides in SCs. Three red boxes on right-hand panels mark two lumenal regions from which GFP signal was measured in these specific images.

(D) Bar chart showing mean number of GFP puncta per box in accessory gland lumen for the four genotypes shown in (C).

All glands were stained with DAPI to mark nuclei. Scale bars = 50 µm. For bar charts, data are mean ± SEM, analysed using the Kruskal-Wallis test; n = animal number above bar, *P<0.05, ns = not significant.
