# Supplementary material for: Amyloid-β disrupts APP-regulated protein aggregation and dissociation from recycling endosomal membranes
Source: EMBO J. 2025 Jul 17;44(16):4443–72. doi: 10.1038/s44318-025-00497-y (PMC12361456; doi:10.1038/s44318-025-00497-y)
Supplement: Supplementary file 6 — Movie EV4 [file 44318_2025_497_MOESM6_ESM.zip › Movie EV4.docx]

**Movie EV4** – **Time-lapse movie of SC DCG biogenesis in *Appl* knockdown cell with *GFP-mfas* marker**, related to Figure 3G. White arrow marks immature DCG-forming compartment. Blue arrow marks mature DCG compartment.
