# Supplementary material for: Amyloid-β disrupts APP-regulated protein aggregation and dissociation from recycling endosomal membranes
Source: EMBO J. 2025 Jul 17;44(16):4443–72. doi: 10.1038/s44318-025-00497-y (PMC12361456; doi:10.1038/s44318-025-00497-y)
Supplement: Supplementary file 7 — Movie EV5 [file 44318_2025_497_MOESM7_ESM.zip › Movie EV5.docx]

**Movie EV5** – **Time-lapse movie of DCG biogenesis in SC expressing Aβ-42 Dutch mutant in *GFP-mfas* genetic background**, related to Figure 6F. White arrow marks immature DCG-forming compartment. Blue arrow marks mature DCG compartment.
