# Supplementary figures and images for: Amyloid-β disrupts APP-regulated protein aggregation and dissociation from recycling endosomal membranes (part 1 of 3)
Source: EMBO J. 2025 Jul 17;44(16):4443–72. doi: 10.1038/s44318-025-00497-y (PMC12361456; doi:10.1038/s44318-025-00497-y)

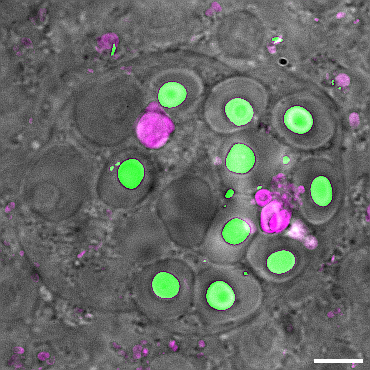

Supplement: Supplementary file 9 — Source data Fig. 1 [file 44318_2025_497_MOESM9_ESM.zip › EMBO_Figure1-Final/1B/GFPmfas_gene_trap_x_w1118_Composite.gif]

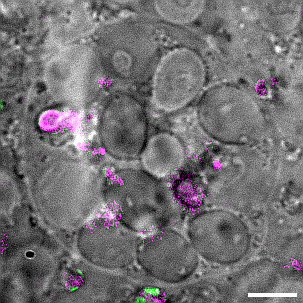

Supplement: Supplementary file 9 — Source data Fig. 1 [file 44318_2025_497_MOESM9_ESM.zip › EMBO_Figure1-Final/1C/mfas#1_16073_x_tdGFPmfas/mfas#1_16073_x_tdGFPmfas_Composite.gif]

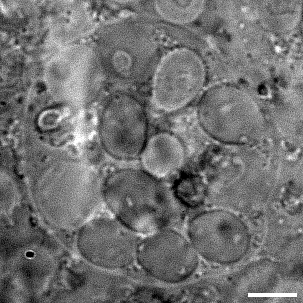

Supplement: Supplementary file 9 — Source data Fig. 1 [file 44318_2025_497_MOESM9_ESM.zip › EMBO_Figure1-Final/1C/mfas#1_16073_x_tdGFPmfas/mfas#1_16073_x_tdGFPmfas_DIC.gif]

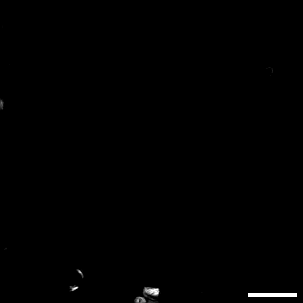

Supplement: Supplementary file 9 — Source data Fig. 1 [file 44318_2025_497_MOESM9_ESM.zip › EMBO_Figure1-Final/1C/mfas#1_16073_x_tdGFPmfas/mfas#1_16073_x_tdGFPmfas_GFP.gif]

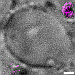

Supplement: Supplementary file 9 — Source data Fig. 1 [file 44318_2025_497_MOESM9_ESM.zip › EMBO_Figure1-Final/1C/mfas#1_16073_x_tdGFPmfas/mfas#1_16073_x_tdGFPmfas_Zoom1.gif]

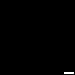

Supplement: Supplementary file 9 — Source data Fig. 1 [file 44318_2025_497_MOESM9_ESM.zip › EMBO_Figure1-Final/1C/mfas#1_16073_x_tdGFPmfas/mfas#1_16073_x_tdGFPmfas_Zoom2.gif]

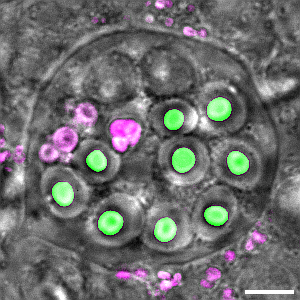

Supplement: Supplementary file 9 — Source data Fig. 1 [file 44318_2025_497_MOESM9_ESM.zip › EMBO_Figure1-Final/1C/rosyRNAi_x_tdGFPmfas/rosyRNAi_x_tdGFPmfas_Composite.gif]

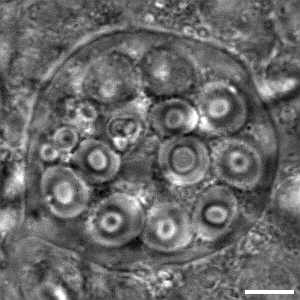

Supplement: Supplementary file 9 — Source data Fig. 1 [file 44318_2025_497_MOESM9_ESM.zip › EMBO_Figure1-Final/1C/rosyRNAi_x_tdGFPmfas/rosyRNAi_x_tdGFPmfas_DIC.gif]

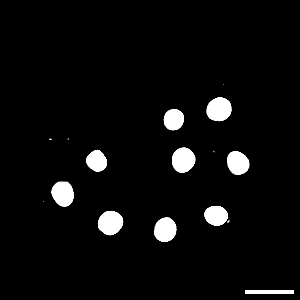

Supplement: Supplementary file 9 — Source data Fig. 1 [file 44318_2025_497_MOESM9_ESM.zip › EMBO_Figure1-Final/1C/rosyRNAi_x_tdGFPmfas/rosyRNAi_x_tdGFPmfas_GFP.gif]

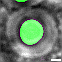

Supplement: Supplementary file 9 — Source data Fig. 1 [file 44318_2025_497_MOESM9_ESM.zip › EMBO_Figure1-Final/1C/rosyRNAi_x_tdGFPmfas/rosyRNAi_x_tdGFPmfas_Zoom1.gif]

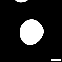

Supplement: Supplementary file 9 — Source data Fig. 1 [file 44318_2025_497_MOESM9_ESM.zip › EMBO_Figure1-Final/1C/rosyRNAi_x_tdGFPmfas/rosyRNAi_x_tdGFPmfas_Zoom2.gif]

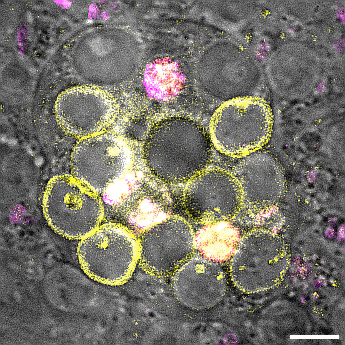

Supplement: Supplementary file 9 — Source data Fig. 1 [file 44318_2025_497_MOESM9_ESM.zip › EMBO_Figure1-Final/1D/mfas#1_16073_x_tdYFPRab11/mfas#1_16073_x_tdYFPRab11_Composite.gif]

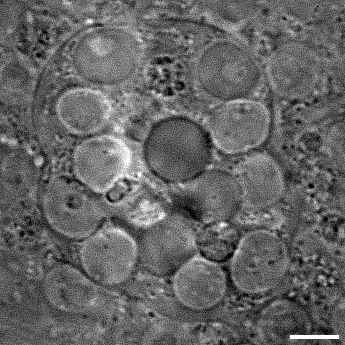

Supplement: Supplementary file 9 — Source data Fig. 1 [file 44318_2025_497_MOESM9_ESM.zip › EMBO_Figure1-Final/1D/mfas#1_16073_x_tdYFPRab11/mfas#1_16073_x_tdYFPRab11_DIC.gif]

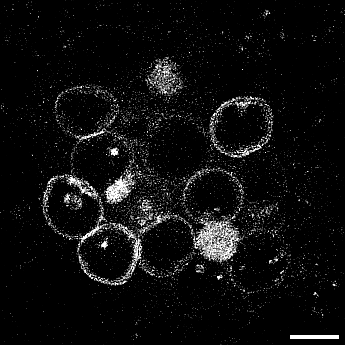

Supplement: Supplementary file 9 — Source data Fig. 1 [file 44318_2025_497_MOESM9_ESM.zip › EMBO_Figure1-Final/1D/mfas#1_16073_x_tdYFPRab11/mfas#1_16073_x_tdYFPRab11_YFP.gif]

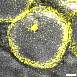

Supplement: Supplementary file 9 — Source data Fig. 1 [file 44318_2025_497_MOESM9_ESM.zip › EMBO_Figure1-Final/1D/mfas#1_16073_x_tdYFPRab11/mfas#1_16073_x_tdYFPRab11_Zoom1.gif]

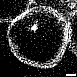

Supplement: Supplementary file 9 — Source data Fig. 1 [file 44318_2025_497_MOESM9_ESM.zip › EMBO_Figure1-Final/1D/mfas#1_16073_x_tdYFPRab11/mfas#1_16073_x_tdYFPRab11_Zoom2.gif]

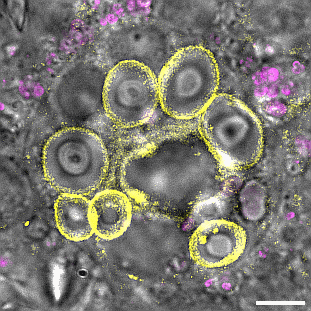

Supplement: Supplementary file 9 — Source data Fig. 1 [file 44318_2025_497_MOESM9_ESM.zip › EMBO_Figure1-Final/1D/rosyRNAi_x_tdYFPRab11/rosyRNAi_x_tdYFPRab11_Composite.gif]

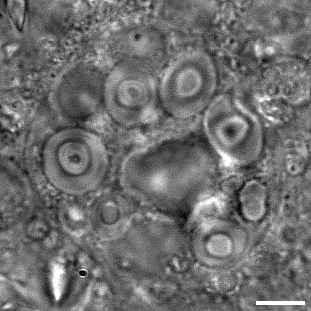

Supplement: Supplementary file 9 — Source data Fig. 1 [file 44318_2025_497_MOESM9_ESM.zip › EMBO_Figure1-Final/1D/rosyRNAi_x_tdYFPRab11/rosyRNAi_x_tdYFPRab11_DIC.gif]

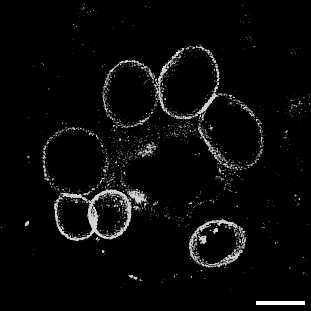

Supplement: Supplementary file 9 — Source data Fig. 1 [file 44318_2025_497_MOESM9_ESM.zip › EMBO_Figure1-Final/1D/rosyRNAi_x_tdYFPRab11/rosyRNAi_x_tdYFPRab11_YFP.gif]

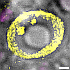

Supplement: Supplementary file 9 — Source data Fig. 1 [file 44318_2025_497_MOESM9_ESM.zip › EMBO_Figure1-Final/1D/rosyRNAi_x_tdYFPRab11/rosyRNAi_x_tdYFPRab11_Zoom1.gif]

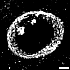

Supplement: Supplementary file 9 — Source data Fig. 1 [file 44318_2025_497_MOESM9_ESM.zip › EMBO_Figure1-Final/1D/rosyRNAi_x_tdYFPRab11/rosyRNAi_x_tdYFPRab11_Zoom2.gif]

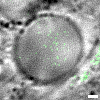

Supplement: Supplementary file 9 — Source data Fig. 1 [file 44318_2025_497_MOESM9_ESM.zip › EMBO_Figure1-Final/1E/w1118_x_tdGFPmfas_Biogenesis_movie_stills/w1118_x_tdGFPmfas_Biogenesis_movie_Composite_Zoom1_t=0.gif]

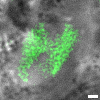

Supplement: Supplementary file 9 — Source data Fig. 1 [file 44318_2025_497_MOESM9_ESM.zip › EMBO_Figure1-Final/1E/w1118_x_tdGFPmfas_Biogenesis_movie_stills/w1118_x_tdGFPmfas_Biogenesis_movie_Composite_Zoom2_t=61.gif]

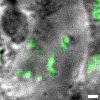

Supplement: Supplementary file 9 — Source data Fig. 1 [file 44318_2025_497_MOESM9_ESM.zip › EMBO_Figure1-Final/1E/w1118_x_tdGFPmfas_Biogenesis_movie_stills/w1118_x_tdGFPmfas_Biogenesis_movie_Composite_Zoom3_t=4.gif]

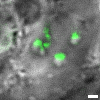

Supplement: Supplementary file 9 — Source data Fig. 1 [file 44318_2025_497_MOESM9_ESM.zip › EMBO_Figure1-Final/1E/w1118_x_tdGFPmfas_Biogenesis_movie_stills/w1118_x_tdGFPmfas_Biogenesis_movie_Composite_Zoom4_t=67.gif]

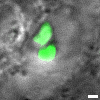

Supplement: Supplementary file 9 — Source data Fig. 1 [file 44318_2025_497_MOESM9_ESM.zip › EMBO_Figure1-Final/1E/w1118_x_tdGFPmfas_Biogenesis_movie_stills/w1118_x_tdGFPmfas_Biogenesis_movie_Composite_Zoom5_t=85.gif]

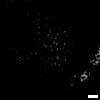

Supplement: Supplementary file 9 — Source data Fig. 1 [file 44318_2025_497_MOESM9_ESM.zip › EMBO_Figure1-Final/1E/w1118_x_tdGFPmfas_Biogenesis_movie_stills/w1118_x_tdGFPmfas_Biogenesis_movie_GFP_Zoom1_t=0.gif]

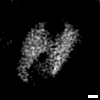

Supplement: Supplementary file 9 — Source data Fig. 1 [file 44318_2025_497_MOESM9_ESM.zip › EMBO_Figure1-Final/1E/w1118_x_tdGFPmfas_Biogenesis_movie_stills/w1118_x_tdGFPmfas_Biogenesis_movie_GFP_Zoom2_t=61.gif]

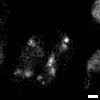

Supplement: Supplementary file 9 — Source data Fig. 1 [file 44318_2025_497_MOESM9_ESM.zip › EMBO_Figure1-Final/1E/w1118_x_tdGFPmfas_Biogenesis_movie_stills/w1118_x_tdGFPmfas_Biogenesis_movie_GFP_Zoom3_t=64.gif]

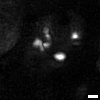

Supplement: Supplementary file 9 — Source data Fig. 1 [file 44318_2025_497_MOESM9_ESM.zip › EMBO_Figure1-Final/1E/w1118_x_tdGFPmfas_Biogenesis_movie_stills/w1118_x_tdGFPmfas_Biogenesis_movie_GFP_Zoom4_t=67.gif]

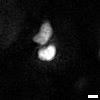

Supplement: Supplementary file 9 — Source data Fig. 1 [file 44318_2025_497_MOESM9_ESM.zip › EMBO_Figure1-Final/1E/w1118_x_tdGFPmfas_Biogenesis_movie_stills/w1118_x_tdGFPmfas_Biogenesis_movie_GFP_Zoom5_t=85.gif]

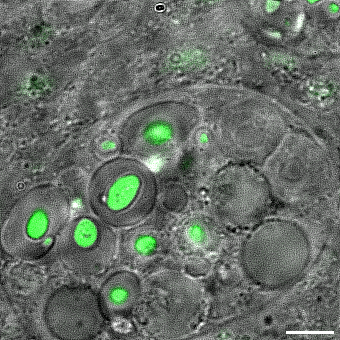

Supplement: Supplementary file 9 — Source data Fig. 1 [file 44318_2025_497_MOESM9_ESM.zip › EMBO_Figure1-Final/1E/w1118_x_tdGFPmfas_Biogenesis_movie_whole-cell_Composite.gif]

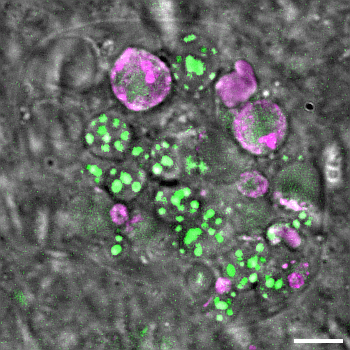

Supplement: Supplementary file 10 — Source data Fig. 2 [file 44318_2025_497_MOESM10_ESM.zip › EMBO_Figure2-Final/2A/GAPDH2RNAi_x_tdGFPmfas/GAPDH2RNAi_x_tdGFPmfas_Composite.gif]

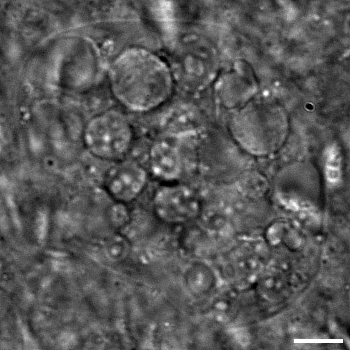

Supplement: Supplementary file 10 — Source data Fig. 2 [file 44318_2025_497_MOESM10_ESM.zip › EMBO_Figure2-Final/2A/GAPDH2RNAi_x_tdGFPmfas/GAPDH2RNAi_x_tdGFPmfas_DIC.gif]

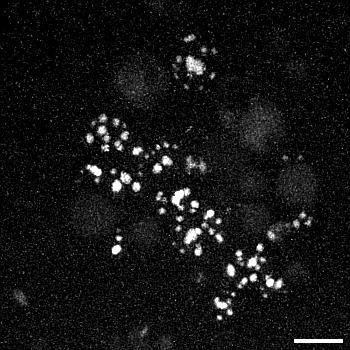

Supplement: Supplementary file 10 — Source data Fig. 2 [file 44318_2025_497_MOESM10_ESM.zip › EMBO_Figure2-Final/2A/GAPDH2RNAi_x_tdGFPmfas/GAPDH2RNAi_x_tdGFPmfas_GFP.gif]

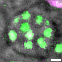

Supplement: Supplementary file 10 — Source data Fig. 2 [file 44318_2025_497_MOESM10_ESM.zip › EMBO_Figure2-Final/2A/GAPDH2RNAi_x_tdGFPmfas/GAPDH2RNAi_x_tdGFPmfas_Zoom1.gif]

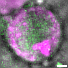

Supplement: Supplementary file 10 — Source data Fig. 2 [file 44318_2025_497_MOESM10_ESM.zip › EMBO_Figure2-Final/2A/GAPDH2RNAi_x_tdGFPmfas/GAPDH2RNAi_x_tdGFPmfas_Zoom2.gif]

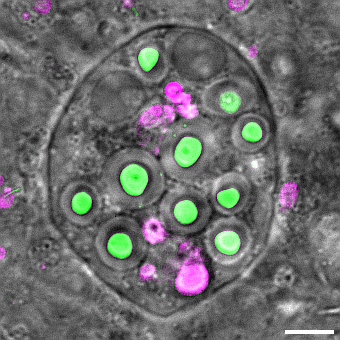

Supplement: Supplementary file 10 — Source data Fig. 2 [file 44318_2025_497_MOESM10_ESM.zip › EMBO_Figure2-Final/2A/rosyRNAi_x_tdGFPmfas/rosyRNAi_x_tdGFPmfas_Composite.gif]

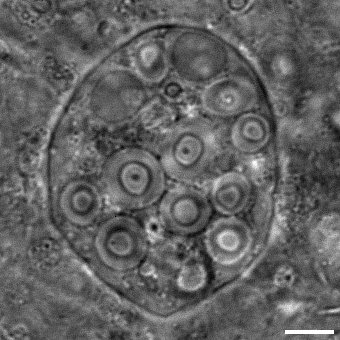

Supplement: Supplementary file 10 — Source data Fig. 2 [file 44318_2025_497_MOESM10_ESM.zip › EMBO_Figure2-Final/2A/rosyRNAi_x_tdGFPmfas/rosyRNAi_x_tdGFPmfas_DIC.gif]

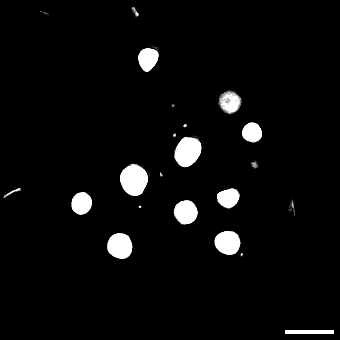

Supplement: Supplementary file 10 — Source data Fig. 2 [file 44318_2025_497_MOESM10_ESM.zip › EMBO_Figure2-Final/2A/rosyRNAi_x_tdGFPmfas/rosyRNAi_x_tdGFPmfas_GFP.gif]

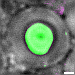

Supplement: Supplementary file 10 — Source data Fig. 2 [file 44318_2025_497_MOESM10_ESM.zip › EMBO_Figure2-Final/2A/rosyRNAi_x_tdGFPmfas/rosyRNAi_x_tdGFPmfas_Zoom1.gif]

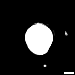

Supplement: Supplementary file 10 — Source data Fig. 2 [file 44318_2025_497_MOESM10_ESM.zip › EMBO_Figure2-Final/2A/rosyRNAi_x_tdGFPmfas/rosyRNAi_x_tdGFPmfas_Zoom2.gif]

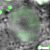

Supplement: Supplementary file 10 — Source data Fig. 2 [file 44318_2025_497_MOESM10_ESM.zip › EMBO_Figure2-Final/2D/GAPDH2RNAi_x_tdGFPmfas_Biogenesis_movie_stills/GAPDH2RNAi_x_tdGFPmfas_Biogenesis_movie_Composite_Zoom1_t=0.gif]

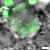

Supplement: Supplementary file 10 — Source data Fig. 2 [file 44318_2025_497_MOESM10_ESM.zip › EMBO_Figure2-Final/2D/GAPDH2RNAi_x_tdGFPmfas_Biogenesis_movie_stills/GAPDH2RNAi_x_tdGFPmfas_Biogenesis_movie_Composite_Zoom2_t=24.gif]

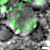

Supplement: Supplementary file 10 — Source data Fig. 2 [file 44318_2025_497_MOESM10_ESM.zip › EMBO_Figure2-Final/2D/GAPDH2RNAi_x_tdGFPmfas_Biogenesis_movie_stills/GAPDH2RNAi_x_tdGFPmfas_Biogenesis_movie_Composite_Zoom3_t=29.gif]

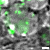

Supplement: Supplementary file 10 — Source data Fig. 2 [file 44318_2025_497_MOESM10_ESM.zip › EMBO_Figure2-Final/2D/GAPDH2RNAi_x_tdGFPmfas_Biogenesis_movie_stills/GAPDH2RNAi_x_tdGFPmfas_Biogenesis_movie_Composite_Zoom4_t=42.gif]

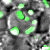

Supplement: Supplementary file 10 — Source data Fig. 2 [file 44318_2025_497_MOESM10_ESM.zip › EMBO_Figure2-Final/2D/GAPDH2RNAi_x_tdGFPmfas_Biogenesis_movie_stills/GAPDH2RNAi_x_tdGFPmfas_Biogenesis_movie_Composite_Zoom5_t=87.gif]

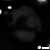

Supplement: Supplementary file 10 — Source data Fig. 2 [file 44318_2025_497_MOESM10_ESM.zip › EMBO_Figure2-Final/2D/GAPDH2RNAi_x_tdGFPmfas_Biogenesis_movie_stills/GAPDH2RNAi_x_tdGFPmfas_Biogenesis_movie_GFP_Zoom1_t=0.gif]

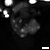

Supplement: Supplementary file 10 — Source data Fig. 2 [file 44318_2025_497_MOESM10_ESM.zip › EMBO_Figure2-Final/2D/GAPDH2RNAi_x_tdGFPmfas_Biogenesis_movie_stills/GAPDH2RNAi_x_tdGFPmfas_Biogenesis_movie_GFP_Zoom2_t=24.gif]

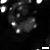

Supplement: Supplementary file 10 — Source data Fig. 2 [file 44318_2025_497_MOESM10_ESM.zip › EMBO_Figure2-Final/2D/GAPDH2RNAi_x_tdGFPmfas_Biogenesis_movie_stills/GAPDH2RNAi_x_tdGFPmfas_Biogenesis_movie_GFP_Zoom3_t=29.gif]

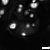

Supplement: Supplementary file 10 — Source data Fig. 2 [file 44318_2025_497_MOESM10_ESM.zip › EMBO_Figure2-Final/2D/GAPDH2RNAi_x_tdGFPmfas_Biogenesis_movie_stills/GAPDH2RNAi_x_tdGFPmfas_Biogenesis_movie_GFP_Zoom4_t=42.gif]

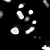

Supplement: Supplementary file 10 — Source data Fig. 2 [file 44318_2025_497_MOESM10_ESM.zip › EMBO_Figure2-Final/2D/GAPDH2RNAi_x_tdGFPmfas_Biogenesis_movie_stills/GAPDH2RNAi_x_tdGFPmfas_Biogenesis_movie_GFP_Zoom5_t=87.gif]

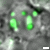

Supplement: Supplementary file 10 — Source data Fig. 2 [file 44318_2025_497_MOESM10_ESM.zip › EMBO_Figure2-Final/2D/GAPDH2RNAi_x_tdGFPmfas_MatureCompartment_movie_stills/GAPDH2RNAi_x_tdGFPmfas_MatureCompartment_movie_Composite_Zoom1_t=0.gif]

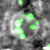

Supplement: Supplementary file 10 — Source data Fig. 2 [file 44318_2025_497_MOESM10_ESM.zip › EMBO_Figure2-Final/2D/GAPDH2RNAi_x_tdGFPmfas_MatureCompartment_movie_stills/GAPDH2RNAi_x_tdGFPmfas_MatureCompartment_movie_Composite_Zoom2_t=23.gif]

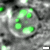

Supplement: Supplementary file 10 — Source data Fig. 2 [file 44318_2025_497_MOESM10_ESM.zip › EMBO_Figure2-Final/2D/GAPDH2RNAi_x_tdGFPmfas_MatureCompartment_movie_stills/GAPDH2RNAi_x_tdGFPmfas_MatureCompartment_movie_Composite_Zoom3_t=46.gif]

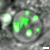

Supplement: Supplementary file 10 — Source data Fig. 2 [file 44318_2025_497_MOESM10_ESM.zip › EMBO_Figure2-Final/2D/GAPDH2RNAi_x_tdGFPmfas_MatureCompartment_movie_stills/GAPDH2RNAi_x_tdGFPmfas_MatureCompartment_movie_Composite_Zoom4_t=69.gif]

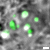

Supplement: Supplementary file 10 — Source data Fig. 2 [file 44318_2025_497_MOESM10_ESM.zip › EMBO_Figure2-Final/2D/GAPDH2RNAi_x_tdGFPmfas_MatureCompartment_movie_stills/GAPDH2RNAi_x_tdGFPmfas_MatureCompartment_movie_Composite_Zoom5_t=91.gif]

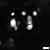

Supplement: Supplementary file 10 — Source data Fig. 2 [file 44318_2025_497_MOESM10_ESM.zip › EMBO_Figure2-Final/2D/GAPDH2RNAi_x_tdGFPmfas_MatureCompartment_movie_stills/GAPDH2RNAi_x_tdGFPmfas_MatureCompartment_movie_GFP_Zoom1_t=0.gif]

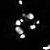

Supplement: Supplementary file 10 — Source data Fig. 2 [file 44318_2025_497_MOESM10_ESM.zip › EMBO_Figure2-Final/2D/GAPDH2RNAi_x_tdGFPmfas_MatureCompartment_movie_stills/GAPDH2RNAi_x_tdGFPmfas_MatureCompartment_movie_GFP_Zoom2_t=23.gif]

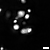

Supplement: Supplementary file 10 — Source data Fig. 2 [file 44318_2025_497_MOESM10_ESM.zip › EMBO_Figure2-Final/2D/GAPDH2RNAi_x_tdGFPmfas_MatureCompartment_movie_stills/GAPDH2RNAi_x_tdGFPmfas_MatureCompartment_movie_GFP_Zoom3_t=46.gif]

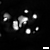

Supplement: Supplementary file 10 — Source data Fig. 2 [file 44318_2025_497_MOESM10_ESM.zip › EMBO_Figure2-Final/2D/GAPDH2RNAi_x_tdGFPmfas_MatureCompartment_movie_stills/GAPDH2RNAi_x_tdGFPmfas_MatureCompartment_movie_GFP_Zoom4_t=69.gif]

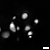

Supplement: Supplementary file 10 — Source data Fig. 2 [file 44318_2025_497_MOESM10_ESM.zip › EMBO_Figure2-Final/2D/GAPDH2RNAi_x_tdGFPmfas_MatureCompartment_movie_stills/GAPDH2RNAi_x_tdGFPmfas_MatureCompartment_movie_GFP_Zoom5_t=91.gif]

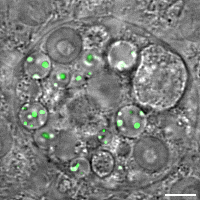

Supplement: Supplementary file 10 — Source data Fig. 2 [file 44318_2025_497_MOESM10_ESM.zip › EMBO_Figure2-Final/2D/GAPDH2RNAi_x_tdGFPmfas_movie_whole-cell_Composite.gif]

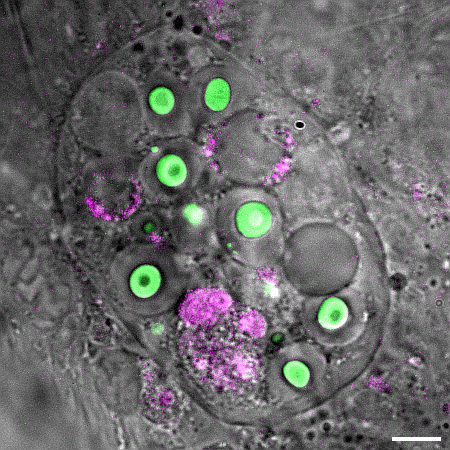

Supplement: Supplementary file 11 — Source data Fig. 3 [file 44318_2025_497_MOESM11_ESM.zip › EMBO_Figure3-Final/3B/APP-YFP;Appl-RNAi (17052)_x_tdGFPmfas/APP-YFP;Appl-RNAi (17052)_x_tdGFPmfas_Composite.gif]

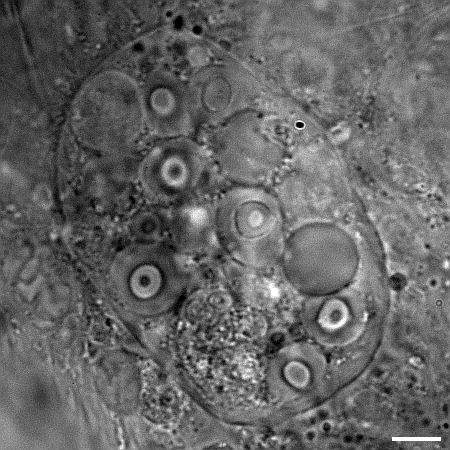

Supplement: Supplementary file 11 — Source data Fig. 3 [file 44318_2025_497_MOESM11_ESM.zip › EMBO_Figure3-Final/3B/APP-YFP;Appl-RNAi (17052)_x_tdGFPmfas/APP-YFP;Appl-RNAi (17052)_x_tdGFPmfas_DIC.gif]

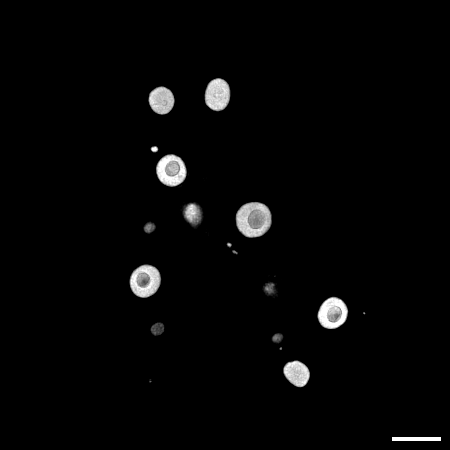

Supplement: Supplementary file 11 — Source data Fig. 3 [file 44318_2025_497_MOESM11_ESM.zip › EMBO_Figure3-Final/3B/APP-YFP;Appl-RNAi (17052)_x_tdGFPmfas/APP-YFP;Appl-RNAi (17052)_x_tdGFPmfas_GFP.gif]

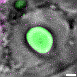

Supplement: Supplementary file 11 — Source data Fig. 3 [file 44318_2025_497_MOESM11_ESM.zip › EMBO_Figure3-Final/3B/APP-YFP;Appl-RNAi (17052)_x_tdGFPmfas/APP-YFP;Appl-RNAi (17052)_x_tdGFPmfas_Zoom1.gif]

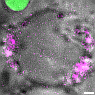

Supplement: Supplementary file 11 — Source data Fig. 3 [file 44318_2025_497_MOESM11_ESM.zip › EMBO_Figure3-Final/3B/APP-YFP;Appl-RNAi (17052)_x_tdGFPmfas/APP-YFP;Appl-RNAi (17052)_x_tdGFPmfas_Zoom2.gif]

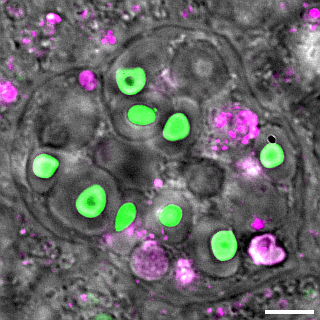

Supplement: Supplementary file 11 — Source data Fig. 3 [file 44318_2025_497_MOESM11_ESM.zip › EMBO_Figure3-Final/3B/APP-YFP_x_tdGFPmfas/APP-YFP_x_tdGFPmfas_Composite.gif]

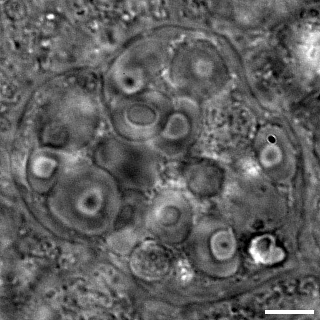

Supplement: Supplementary file 11 — Source data Fig. 3 [file 44318_2025_497_MOESM11_ESM.zip › EMBO_Figure3-Final/3B/APP-YFP_x_tdGFPmfas/APP-YFP_x_tdGFPmfas_DIC.gif]

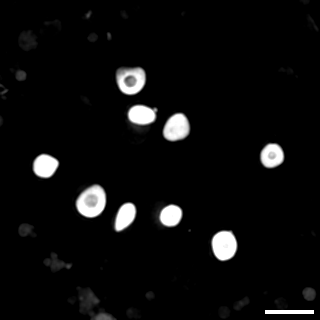

Supplement: Supplementary file 11 — Source data Fig. 3 [file 44318_2025_497_MOESM11_ESM.zip › EMBO_Figure3-Final/3B/APP-YFP_x_tdGFPmfas/APP-YFP_x_tdGFPmfas_GFP.gif]

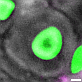

Supplement: Supplementary file 11 — Source data Fig. 3 [file 44318_2025_497_MOESM11_ESM.zip › EMBO_Figure3-Final/3B/APP-YFP_x_tdGFPmfas/APP-YFP_x_tdGFPmfas_Zoom1.gif]

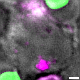

Supplement: Supplementary file 11 — Source data Fig. 3 [file 44318_2025_497_MOESM11_ESM.zip › EMBO_Figure3-Final/3B/APP-YFP_x_tdGFPmfas/APP-YFP_x_tdGFPmfas_Zoom2.gif]

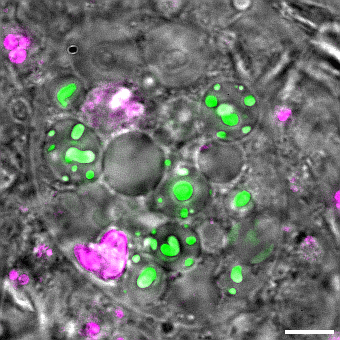

Supplement: Supplementary file 11 — Source data Fig. 3 [file 44318_2025_497_MOESM11_ESM.zip › EMBO_Figure3-Final/3B/Appl-RNAi (17052)_x_tdGFPmfas/Appl-RNAi (17052)_x_tdGFPmfas_Composite.gif]

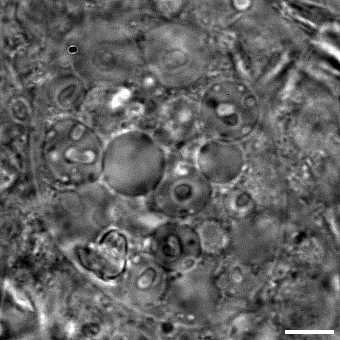

Supplement: Supplementary file 11 — Source data Fig. 3 [file 44318_2025_497_MOESM11_ESM.zip › EMBO_Figure3-Final/3B/Appl-RNAi (17052)_x_tdGFPmfas/Appl-RNAi (17052)_x_tdGFPmfas_DIC.gif]

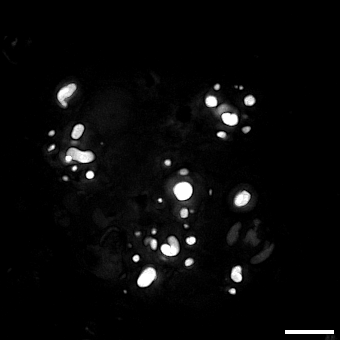

Supplement: Supplementary file 11 — Source data Fig. 3 [file 44318_2025_497_MOESM11_ESM.zip › EMBO_Figure3-Final/3B/Appl-RNAi (17052)_x_tdGFPmfas/Appl-RNAi (17052)_x_tdGFPmfas_GFP.gif]

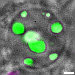

Supplement: Supplementary file 11 — Source data Fig. 3 [file 44318_2025_497_MOESM11_ESM.zip › EMBO_Figure3-Final/3B/Appl-RNAi (17052)_x_tdGFPmfas/Appl-RNAi (17052)_x_tdGFPmfas_Zoom1.gif]

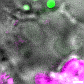

Supplement: Supplementary file 11 — Source data Fig. 3 [file 44318_2025_497_MOESM11_ESM.zip › EMBO_Figure3-Final/3B/Appl-RNAi (17052)_x_tdGFPmfas/Appl-RNAi (17052)_x_tdGFPmfas_Zoom2.gif]

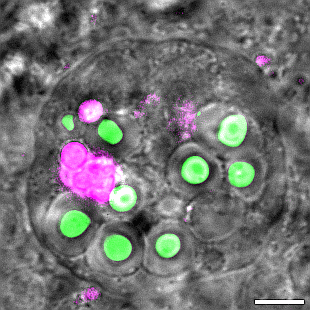

Supplement: Supplementary file 11 — Source data Fig. 3 [file 44318_2025_497_MOESM11_ESM.zip › EMBO_Figure3-Final/3B/rosyRNAi_x_tdGFPmfas/rosyRNAi_x_tdGFPmfas_Composite.gif]

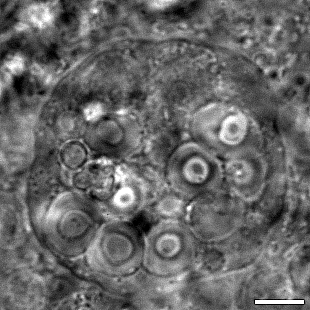

Supplement: Supplementary file 11 — Source data Fig. 3 [file 44318_2025_497_MOESM11_ESM.zip › EMBO_Figure3-Final/3B/rosyRNAi_x_tdGFPmfas/rosyRNAi_x_tdGFPmfas_DIC.gif]

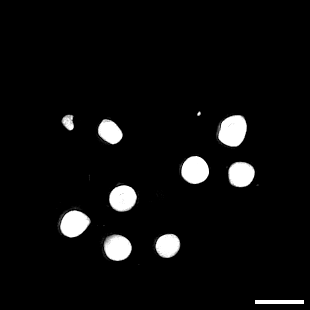

Supplement: Supplementary file 11 — Source data Fig. 3 [file 44318_2025_497_MOESM11_ESM.zip › EMBO_Figure3-Final/3B/rosyRNAi_x_tdGFPmfas/rosyRNAi_x_tdGFPmfas_GFP.gif]

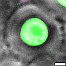

Supplement: Supplementary file 11 — Source data Fig. 3 [file 44318_2025_497_MOESM11_ESM.zip › EMBO_Figure3-Final/3B/rosyRNAi_x_tdGFPmfas/rosyRNAi_x_tdGFPmfas_Zoom1.gif]

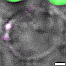

Supplement: Supplementary file 11 — Source data Fig. 3 [file 44318_2025_497_MOESM11_ESM.zip › EMBO_Figure3-Final/3B/rosyRNAi_x_tdGFPmfas/rosyRNAi_x_tdGFPmfas_Zoom2.gif]

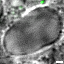

Supplement: Supplementary file 11 — Source data Fig. 3 [file 44318_2025_497_MOESM11_ESM.zip › EMBO_Figure3-Final/3G/Appl-RNAi (17052)_x_tdGFPmfas_Biogenesis_movie_stills/Appl-RNAi (17052)_x_tdGFPmfas_Biogenesis_movie_Composite_Zoom1_t=0.gif]

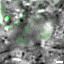

Supplement: Supplementary file 11 — Source data Fig. 3 [file 44318_2025_497_MOESM11_ESM.zip › EMBO_Figure3-Final/3G/Appl-RNAi (17052)_x_tdGFPmfas_Biogenesis_movie_stills/Appl-RNAi (17052)_x_tdGFPmfas_Biogenesis_movie_Composite_Zoom2_t=85.gif]

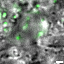

Supplement: Supplementary file 11 — Source data Fig. 3 [file 44318_2025_497_MOESM11_ESM.zip › EMBO_Figure3-Final/3G/Appl-RNAi (17052)_x_tdGFPmfas_Biogenesis_movie_stills/Appl-RNAi (17052)_x_tdGFPmfas_Biogenesis_movie_Composite_Zoom3_t=87.gif]

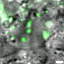

Supplement: Supplementary file 11 — Source data Fig. 3 [file 44318_2025_497_MOESM11_ESM.zip › EMBO_Figure3-Final/3G/Appl-RNAi (17052)_x_tdGFPmfas_Biogenesis_movie_stills/Appl-RNAi (17052)_x_tdGFPmfas_Biogenesis_movie_Composite_Zoom4_t=91.gif]

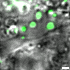

Supplement: Supplementary file 11 — Source data Fig. 3 [file 44318_2025_497_MOESM11_ESM.zip › EMBO_Figure3-Final/3G/Appl-RNAi (17052)_x_tdGFPmfas_Biogenesis_movie_stills/Appl-RNAi (17052)_x_tdGFPmfas_Biogenesis_movie_Composite_Zoom5_t=180.gif]

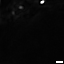

Supplement: Supplementary file 11 — Source data Fig. 3 [file 44318_2025_497_MOESM11_ESM.zip › EMBO_Figure3-Final/3G/Appl-RNAi (17052)_x_tdGFPmfas_Biogenesis_movie_stills/Appl-RNAi (17052)_x_tdGFPmfas_Biogenesis_movie_GFP_Zoom1_t=0.gif]

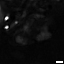

Supplement: Supplementary file 11 — Source data Fig. 3 [file 44318_2025_497_MOESM11_ESM.zip › EMBO_Figure3-Final/3G/Appl-RNAi (17052)_x_tdGFPmfas_Biogenesis_movie_stills/Appl-RNAi (17052)_x_tdGFPmfas_Biogenesis_movie_GFP_Zoom2_t=85.gif]

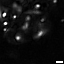

Supplement: Supplementary file 11 — Source data Fig. 3 [file 44318_2025_497_MOESM11_ESM.zip › EMBO_Figure3-Final/3G/Appl-RNAi (17052)_x_tdGFPmfas_Biogenesis_movie_stills/Appl-RNAi (17052)_x_tdGFPmfas_Biogenesis_movie_GFP_Zoom3_t=87.gif]

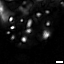

Supplement: Supplementary file 11 — Source data Fig. 3 [file 44318_2025_497_MOESM11_ESM.zip › EMBO_Figure3-Final/3G/Appl-RNAi (17052)_x_tdGFPmfas_Biogenesis_movie_stills/Appl-RNAi (17052)_x_tdGFPmfas_Biogenesis_movie_GFP_Zoom4_t=91.gif]

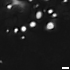

Supplement: Supplementary file 11 — Source data Fig. 3 [file 44318_2025_497_MOESM11_ESM.zip › EMBO_Figure3-Final/3G/Appl-RNAi (17052)_x_tdGFPmfas_Biogenesis_movie_stills/Appl-RNAi (17052)_x_tdGFPmfas_Biogenesis_movie_GFP_Zoom5_t=180.gif]

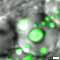

Supplement: Supplementary file 11 — Source data Fig. 3 [file 44318_2025_497_MOESM11_ESM.zip › EMBO_Figure3-Final/3G/Appl-RNAi (17052)_x_tdGFPmfas_MatureCompartment_movie_stills/Appl-RNAi (17052)_x_tdGFPmfas_MatureCompartment_movie_Composite_Zoom1_t=0.gif]

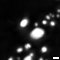

Supplement: Supplementary file 11 — Source data Fig. 3 [file 44318_2025_497_MOESM11_ESM.zip › EMBO_Figure3-Final/3G/Appl-RNAi (17052)_x_tdGFPmfas_MatureCompartment_movie_stills/Appl-RNAi (17052)_x_tdGFPmfas_MatureCompartment_movie_GFP_Zoom1_t=0.gif]

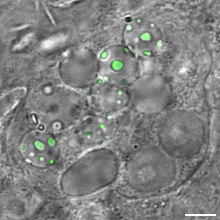

Supplement: Supplementary file 11 — Source data Fig. 3 [file 44318_2025_497_MOESM11_ESM.zip › EMBO_Figure3-Final/3G/Appl-RNAi (17052)_x_tdGFPmfas_movie_whole-cell_Composite.gif]

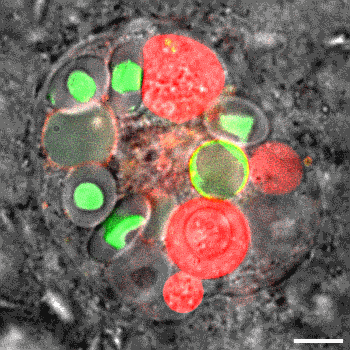

Supplement: Supplementary file 12 — Source data Fig. 4 [file 44318_2025_497_MOESM12_ESM.zip › EMBO_Figure4-Final/4B/dtAppl (GFP-nAPPLc-RFP)_x_td_Whole-cell/dtAppl (GFP-nAPPLc-RFP)_x_td_Composite.gif]

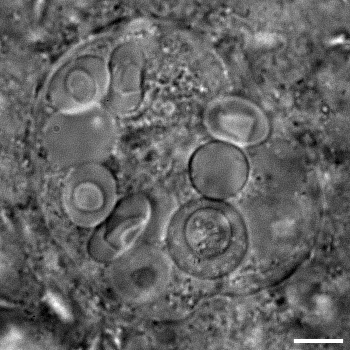

Supplement: Supplementary file 12 — Source data Fig. 4 [file 44318_2025_497_MOESM12_ESM.zip › EMBO_Figure4-Final/4B/dtAppl (GFP-nAPPLc-RFP)_x_td_Whole-cell/dtAppl (GFP-nAPPLc-RFP)_x_td_DIC.gif]

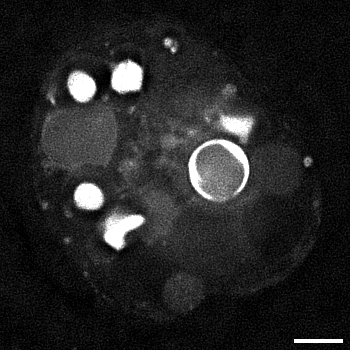

Supplement: Supplementary file 12 — Source data Fig. 4 [file 44318_2025_497_MOESM12_ESM.zip › EMBO_Figure4-Final/4B/dtAppl (GFP-nAPPLc-RFP)_x_td_Whole-cell/dtAppl (GFP-nAPPLc-RFP)_x_td_GFP.gif]

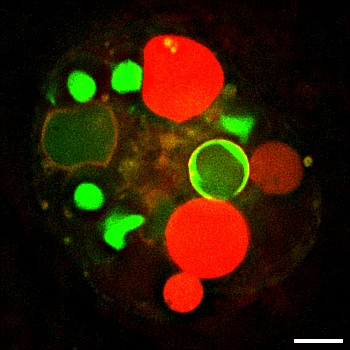

Supplement: Supplementary file 12 — Source data Fig. 4 [file 44318_2025_497_MOESM12_ESM.zip › EMBO_Figure4-Final/4B/dtAppl (GFP-nAPPLc-RFP)_x_td_Whole-cell/dtAppl (GFP-nAPPLc-RFP)_x_td_Merge.gif]
