# Supplementary figures and images for: Amyloid-β disrupts APP-regulated protein aggregation and dissociation from recycling endosomal membranes (part 2 of 3)
Source: EMBO J. 2025 Jul 17;44(16):4443–72. doi: 10.1038/s44318-025-00497-y (PMC12361456; doi:10.1038/s44318-025-00497-y)

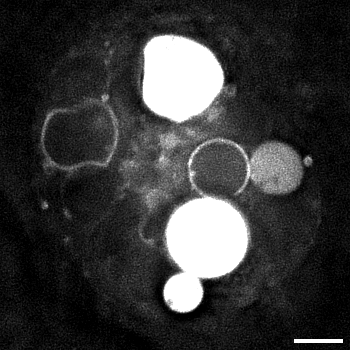

Supplement: Supplementary file 12 — Source data Fig. 4 [file 44318_2025_497_MOESM12_ESM.zip › EMBO_Figure4-Final/4B/dtAppl (GFP-nAPPLc-RFP)_x_td_Whole-cell/dtAppl (GFP-nAPPLc-RFP)_x_td_RFP.gif]

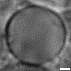

Supplement: Supplementary file 12 — Source data Fig. 4 [file 44318_2025_497_MOESM12_ESM.zip › EMBO_Figure4-Final/4B/dtAppl (GFP-nAPPLc-RFP)_x_td_Zoom1/dtAppl (GFP-nAPPLc-RFP)_x_td_Zoom1_DIC.gif]

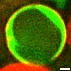

Supplement: Supplementary file 12 — Source data Fig. 4 [file 44318_2025_497_MOESM12_ESM.zip › EMBO_Figure4-Final/4B/dtAppl (GFP-nAPPLc-RFP)_x_td_Zoom1/dtAppl (GFP-nAPPLc-RFP)_x_td_Zoom1_GFP+RFP.gif]

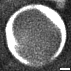

Supplement: Supplementary file 12 — Source data Fig. 4 [file 44318_2025_497_MOESM12_ESM.zip › EMBO_Figure4-Final/4B/dtAppl (GFP-nAPPLc-RFP)_x_td_Zoom1/dtAppl (GFP-nAPPLc-RFP)_x_td_Zoom1_GFP.gif]

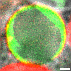

Supplement: Supplementary file 12 — Source data Fig. 4 [file 44318_2025_497_MOESM12_ESM.zip › EMBO_Figure4-Final/4B/dtAppl (GFP-nAPPLc-RFP)_x_td_Zoom1/dtAppl (GFP-nAPPLc-RFP)_x_td_Zoom1_Merge.gif]

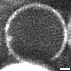

Supplement: Supplementary file 12 — Source data Fig. 4 [file 44318_2025_497_MOESM12_ESM.zip › EMBO_Figure4-Final/4B/dtAppl (GFP-nAPPLc-RFP)_x_td_Zoom1/dtAppl (GFP-nAPPLc-RFP)_x_td_Zoom1_RFP.gif]

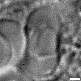

Supplement: Supplementary file 12 — Source data Fig. 4 [file 44318_2025_497_MOESM12_ESM.zip › EMBO_Figure4-Final/4B/dtAppl (GFP-nAPPLc-RFP)_x_td_Zoom2/dtAppl (GFP-nAPPLc-RFP)_x_td_Zoom2_DIC.gif]

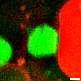

Supplement: Supplementary file 12 — Source data Fig. 4 [file 44318_2025_497_MOESM12_ESM.zip › EMBO_Figure4-Final/4B/dtAppl (GFP-nAPPLc-RFP)_x_td_Zoom2/dtAppl (GFP-nAPPLc-RFP)_x_td_Zoom2_GFP+RFP.gif]

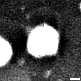

Supplement: Supplementary file 12 — Source data Fig. 4 [file 44318_2025_497_MOESM12_ESM.zip › EMBO_Figure4-Final/4B/dtAppl (GFP-nAPPLc-RFP)_x_td_Zoom2/dtAppl (GFP-nAPPLc-RFP)_x_td_Zoom2_GFP.gif]

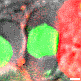

Supplement: Supplementary file 12 — Source data Fig. 4 [file 44318_2025_497_MOESM12_ESM.zip › EMBO_Figure4-Final/4B/dtAppl (GFP-nAPPLc-RFP)_x_td_Zoom2/dtAppl (GFP-nAPPLc-RFP)_x_td_Zoom2_Merge.gif]

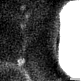

Supplement: Supplementary file 12 — Source data Fig. 4 [file 44318_2025_497_MOESM12_ESM.zip › EMBO_Figure4-Final/4B/dtAppl (GFP-nAPPLc-RFP)_x_td_Zoom2/dtAppl (GFP-nAPPLc-RFP)_x_td_Zoom2_RFP.gif]

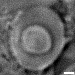

Supplement: Supplementary file 12 — Source data Fig. 4 [file 44318_2025_497_MOESM12_ESM.zip › EMBO_Figure4-Final/4B/dtAppl (GFP-nAPPLc-RFP)_x_td_Zoom3/dtAppl (GFP-nAPPLc-RFP)_x_td_Zoom3_DIC.gif]

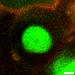

Supplement: Supplementary file 12 — Source data Fig. 4 [file 44318_2025_497_MOESM12_ESM.zip › EMBO_Figure4-Final/4B/dtAppl (GFP-nAPPLc-RFP)_x_td_Zoom3/dtAppl (GFP-nAPPLc-RFP)_x_td_Zoom3_GFP+RFP.gif]

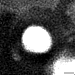

Supplement: Supplementary file 12 — Source data Fig. 4 [file 44318_2025_497_MOESM12_ESM.zip › EMBO_Figure4-Final/4B/dtAppl (GFP-nAPPLc-RFP)_x_td_Zoom3/dtAppl (GFP-nAPPLc-RFP)_x_td_Zoom3_GFP.gif]

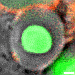

Supplement: Supplementary file 12 — Source data Fig. 4 [file 44318_2025_497_MOESM12_ESM.zip › EMBO_Figure4-Final/4B/dtAppl (GFP-nAPPLc-RFP)_x_td_Zoom3/dtAppl (GFP-nAPPLc-RFP)_x_td_Zoom3_Merge.gif]

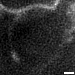

Supplement: Supplementary file 12 — Source data Fig. 4 [file 44318_2025_497_MOESM12_ESM.zip › EMBO_Figure4-Final/4B/dtAppl (GFP-nAPPLc-RFP)_x_td_Zoom3/dtAppl (GFP-nAPPLc-RFP)_x_td_Zoom3_RFP.gif]

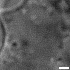

Supplement: Supplementary file 12 — Source data Fig. 4 [file 44318_2025_497_MOESM12_ESM.zip › EMBO_Figure4-Final/4B/dtAppl (GFP-nAPPLc-RFP)_x_td_Zoom4/dtAppl (GFP-nAPPLc-RFP)_x_td_Zoom4_DIC.gif]

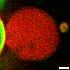

Supplement: Supplementary file 12 — Source data Fig. 4 [file 44318_2025_497_MOESM12_ESM.zip › EMBO_Figure4-Final/4B/dtAppl (GFP-nAPPLc-RFP)_x_td_Zoom4/dtAppl (GFP-nAPPLc-RFP)_x_td_Zoom4_GFP+RFP.gif]

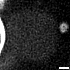

Supplement: Supplementary file 12 — Source data Fig. 4 [file 44318_2025_497_MOESM12_ESM.zip › EMBO_Figure4-Final/4B/dtAppl (GFP-nAPPLc-RFP)_x_td_Zoom4/dtAppl (GFP-nAPPLc-RFP)_x_td_Zoom4_GFP.gif]

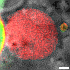

Supplement: Supplementary file 12 — Source data Fig. 4 [file 44318_2025_497_MOESM12_ESM.zip › EMBO_Figure4-Final/4B/dtAppl (GFP-nAPPLc-RFP)_x_td_Zoom4/dtAppl (GFP-nAPPLc-RFP)_x_td_Zoom4_Merge.gif]

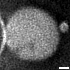

Supplement: Supplementary file 12 — Source data Fig. 4 [file 44318_2025_497_MOESM12_ESM.zip › EMBO_Figure4-Final/4B/dtAppl (GFP-nAPPLc-RFP)_x_td_Zoom4/dtAppl (GFP-nAPPLc-RFP)_x_td_Zoom4_RFP.gif]

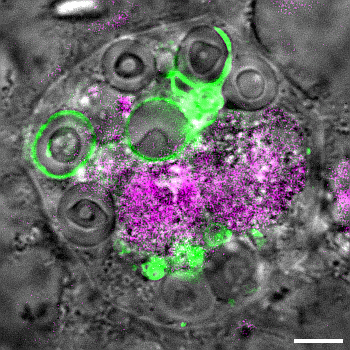

Supplement: Supplementary file 12 — Source data Fig. 4 [file 44318_2025_497_MOESM12_ESM.zip › EMBO_Figure4-Final/4C/nAPPLc-GFP_x_td_Whole-cell/nAPPLc-GFP_x_td_Composite.gif]

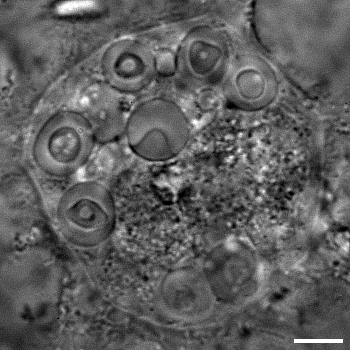

Supplement: Supplementary file 12 — Source data Fig. 4 [file 44318_2025_497_MOESM12_ESM.zip › EMBO_Figure4-Final/4C/nAPPLc-GFP_x_td_Whole-cell/nAPPLc-GFP_x_td_DIC.gif]

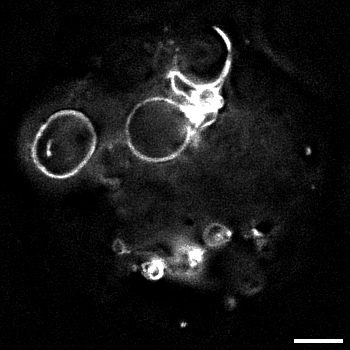

Supplement: Supplementary file 12 — Source data Fig. 4 [file 44318_2025_497_MOESM12_ESM.zip › EMBO_Figure4-Final/4C/nAPPLc-GFP_x_td_Whole-cell/nAPPLc-GFP_x_td_GFP.gif]

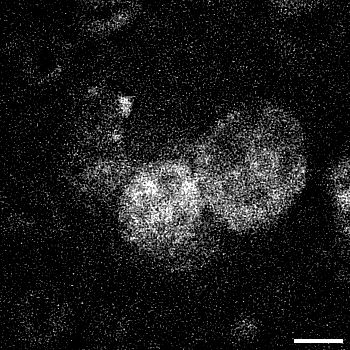

Supplement: Supplementary file 12 — Source data Fig. 4 [file 44318_2025_497_MOESM12_ESM.zip › EMBO_Figure4-Final/4C/nAPPLc-GFP_x_td_Whole-cell/nAPPLc-GFP_x_td_Lys.gif]

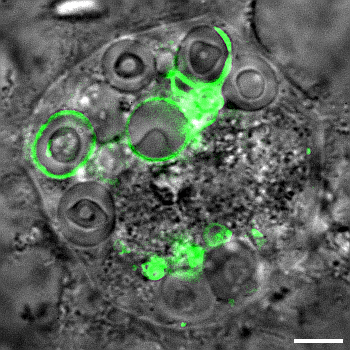

Supplement: Supplementary file 12 — Source data Fig. 4 [file 44318_2025_497_MOESM12_ESM.zip › EMBO_Figure4-Final/4C/nAPPLc-GFP_x_td_Whole-cell/nAPPLc-GFP_x_td_Merge.gif]

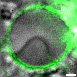

Supplement: Supplementary file 12 — Source data Fig. 4 [file 44318_2025_497_MOESM12_ESM.zip › EMBO_Figure4-Final/4C/nAPPLc-GFP_x_td_Zoom1/nAPPLc-GFP_x_td_Zoom1_DIC+GFP.gif]

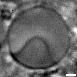

Supplement: Supplementary file 12 — Source data Fig. 4 [file 44318_2025_497_MOESM12_ESM.zip › EMBO_Figure4-Final/4C/nAPPLc-GFP_x_td_Zoom1/nAPPLc-GFP_x_td_Zoom1_DIC.gif]

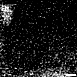

Supplement: Supplementary file 12 — Source data Fig. 4 [file 44318_2025_497_MOESM12_ESM.zip › EMBO_Figure4-Final/4C/nAPPLc-GFP_x_td_Zoom1/nAPPLc-GFP_x_td_Zoom1_Lys.gif]

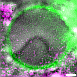

Supplement: Supplementary file 12 — Source data Fig. 4 [file 44318_2025_497_MOESM12_ESM.zip › EMBO_Figure4-Final/4C/nAPPLc-GFP_x_td_Zoom1/nAPPLc-GFP_x_td_Zoom1_Merge.gif]

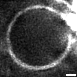

Supplement: Supplementary file 12 — Source data Fig. 4 [file 44318_2025_497_MOESM12_ESM.zip › EMBO_Figure4-Final/4C/nAPPLc-GFP_x_td_Zoom1/nAPPLc-GFP_x_td_Zoom1_RFP.gif]

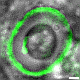

Supplement: Supplementary file 12 — Source data Fig. 4 [file 44318_2025_497_MOESM12_ESM.zip › EMBO_Figure4-Final/4C/nAPPLc-GFP_x_td_Zoom2/nAPPLc-GFP_x_td_Zoom2_DIC+GFP.gif]

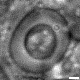

Supplement: Supplementary file 12 — Source data Fig. 4 [file 44318_2025_497_MOESM12_ESM.zip › EMBO_Figure4-Final/4C/nAPPLc-GFP_x_td_Zoom2/nAPPLc-GFP_x_td_Zoom2_DIC.gif]

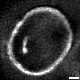

Supplement: Supplementary file 12 — Source data Fig. 4 [file 44318_2025_497_MOESM12_ESM.zip › EMBO_Figure4-Final/4C/nAPPLc-GFP_x_td_Zoom2/nAPPLc-GFP_x_td_Zoom2_GFP.gif]

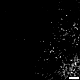

Supplement: Supplementary file 12 — Source data Fig. 4 [file 44318_2025_497_MOESM12_ESM.zip › EMBO_Figure4-Final/4C/nAPPLc-GFP_x_td_Zoom2/nAPPLc-GFP_x_td_Zoom2_Lys.gif]

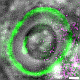

Supplement: Supplementary file 12 — Source data Fig. 4 [file 44318_2025_497_MOESM12_ESM.zip › EMBO_Figure4-Final/4C/nAPPLc-GFP_x_td_Zoom2/nAPPLc-GFP_x_td_Zoom2_Merge.gif]

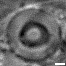

Supplement: Supplementary file 12 — Source data Fig. 4 [file 44318_2025_497_MOESM12_ESM.zip › EMBO_Figure4-Final/4C/nAPPLc-GFP_x_td_Zoom3/nAPPLc-GFP_x_td_Zoom1__Zoom3_DIC.gif]

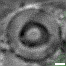

Supplement: Supplementary file 12 — Source data Fig. 4 [file 44318_2025_497_MOESM12_ESM.zip › EMBO_Figure4-Final/4C/nAPPLc-GFP_x_td_Zoom3/nAPPLc-GFP_x_td_Zoom3_DIC+GFP.gif]

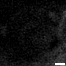

Supplement: Supplementary file 12 — Source data Fig. 4 [file 44318_2025_497_MOESM12_ESM.zip › EMBO_Figure4-Final/4C/nAPPLc-GFP_x_td_Zoom3/nAPPLc-GFP_x_td_Zoom3_GFP.gif]

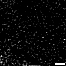

Supplement: Supplementary file 12 — Source data Fig. 4 [file 44318_2025_497_MOESM12_ESM.zip › EMBO_Figure4-Final/4C/nAPPLc-GFP_x_td_Zoom3/nAPPLc-GFP_x_td_Zoom3_Lys.gif]

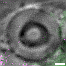

Supplement: Supplementary file 12 — Source data Fig. 4 [file 44318_2025_497_MOESM12_ESM.zip › EMBO_Figure4-Final/4C/nAPPLc-GFP_x_td_Zoom3/nAPPLc-GFP_x_td_Zoom3_Merge.gif]

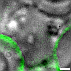

Supplement: Supplementary file 12 — Source data Fig. 4 [file 44318_2025_497_MOESM12_ESM.zip › EMBO_Figure4-Final/4C/nAPPLc-GFP_x_td_Zoom4/nAPPLc-GFP_x_td_Zoom4_DIC+GFP.gif]

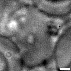

Supplement: Supplementary file 12 — Source data Fig. 4 [file 44318_2025_497_MOESM12_ESM.zip › EMBO_Figure4-Final/4C/nAPPLc-GFP_x_td_Zoom4/nAPPLc-GFP_x_td_Zoom4_DIC.gif]

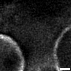

Supplement: Supplementary file 12 — Source data Fig. 4 [file 44318_2025_497_MOESM12_ESM.zip › EMBO_Figure4-Final/4C/nAPPLc-GFP_x_td_Zoom4/nAPPLc-GFP_x_td_Zoom4_GFP.gif]

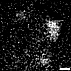

Supplement: Supplementary file 12 — Source data Fig. 4 [file 44318_2025_497_MOESM12_ESM.zip › EMBO_Figure4-Final/4C/nAPPLc-GFP_x_td_Zoom4/nAPPLc-GFP_x_td_Zoom4_Lys.gif]

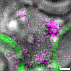

Supplement: Supplementary file 12 — Source data Fig. 4 [file 44318_2025_497_MOESM12_ESM.zip › EMBO_Figure4-Final/4C/nAPPLc-GFP_x_td_Zoom4/nAPPLc-GFP_x_td_Zoom4_Merge.gif]

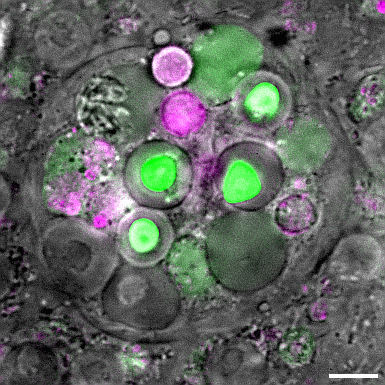

Supplement: Supplementary file 13 — Source data Fig. 5 [file 44318_2025_497_MOESM13_ESM.zip › EMBO_Figure5-Final/5B/Appl-sd_x_tdGFPmfas/Appl-sd_x_tdGFPmfas_Composite.gif]

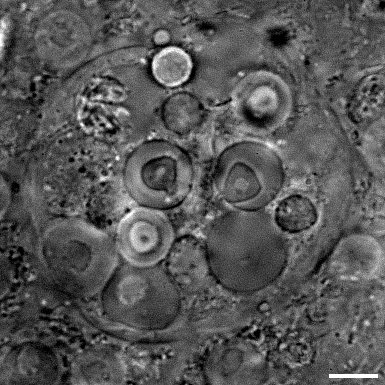

Supplement: Supplementary file 13 — Source data Fig. 5 [file 44318_2025_497_MOESM13_ESM.zip › EMBO_Figure5-Final/5B/Appl-sd_x_tdGFPmfas/Appl-sd_x_tdGFPmfas_DIC.gif]

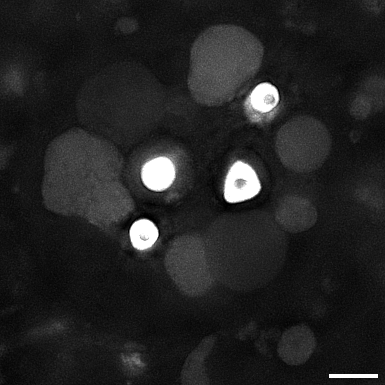

Supplement: Supplementary file 13 — Source data Fig. 5 [file 44318_2025_497_MOESM13_ESM.zip › EMBO_Figure5-Final/5B/Appl-sd_x_tdGFPmfas/Appl-sd_x_tdGFPmfas_GFP.gif]

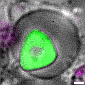

Supplement: Supplementary file 13 — Source data Fig. 5 [file 44318_2025_497_MOESM13_ESM.zip › EMBO_Figure5-Final/5B/Appl-sd_x_tdGFPmfas/Appl-sd_x_tdGFPmfas_Zoom1.gif]

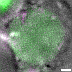

Supplement: Supplementary file 13 — Source data Fig. 5 [file 44318_2025_497_MOESM13_ESM.zip › EMBO_Figure5-Final/5B/Appl-sd_x_tdGFPmfas/Appl-sd_x_tdGFPmfas_Zoom2.gif]

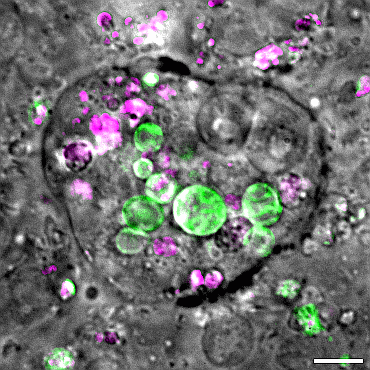

Supplement: Supplementary file 13 — Source data Fig. 5 [file 44318_2025_497_MOESM13_ESM.zip › EMBO_Figure5-Final/5B/Appl-sdE1_x_tdGFPmfas/Appl-sdE1_x_tdGFPmfas_Composite.gif]

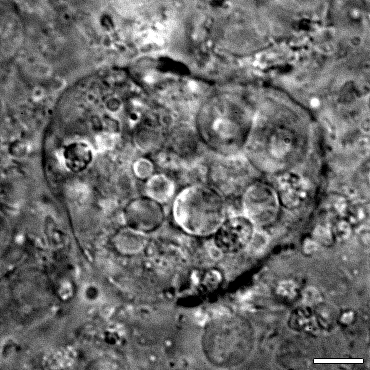

Supplement: Supplementary file 13 — Source data Fig. 5 [file 44318_2025_497_MOESM13_ESM.zip › EMBO_Figure5-Final/5B/Appl-sdE1_x_tdGFPmfas/Appl-sdE1_x_tdGFPmfas_DIC.gif]

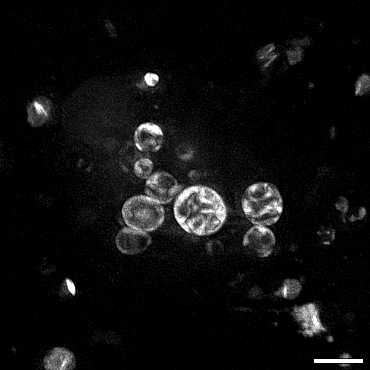

Supplement: Supplementary file 13 — Source data Fig. 5 [file 44318_2025_497_MOESM13_ESM.zip › EMBO_Figure5-Final/5B/Appl-sdE1_x_tdGFPmfas/Appl-sdE1_x_tdGFPmfas_GFP.gif]

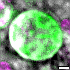

Supplement: Supplementary file 13 — Source data Fig. 5 [file 44318_2025_497_MOESM13_ESM.zip › EMBO_Figure5-Final/5B/Appl-sdE1_x_tdGFPmfas/Appl-sdE1_x_tdGFPmfas_Zoom1.gif]

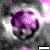

Supplement: Supplementary file 13 — Source data Fig. 5 [file 44318_2025_497_MOESM13_ESM.zip › EMBO_Figure5-Final/5B/Appl-sdE1_x_tdGFPmfas/Appl-sdE1_x_tdGFPmfas_Zoom2.gif]

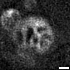

Supplement: Supplementary file 13 — Source data Fig. 5 [file 44318_2025_497_MOESM13_ESM.zip › EMBO_Figure5-Final/5B/Appl-sdE1_x_tdGFPmfas/Appl-sdE1_x_tdGFPmfas_Zoom_z=0um/Appl-sdE1_x_tdGFPmfas_z=0um_Zoom1.gif]

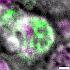

Supplement: Supplementary file 13 — Source data Fig. 5 [file 44318_2025_497_MOESM13_ESM.zip › EMBO_Figure5-Final/5B/Appl-sdE1_x_tdGFPmfas/Appl-sdE1_x_tdGFPmfas_Zoom_z=0um/Appl-sdE1_x_tdGFPmfas_z=0um_Zoom2.gif]

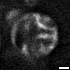

Supplement: Supplementary file 13 — Source data Fig. 5 [file 44318_2025_497_MOESM13_ESM.zip › EMBO_Figure5-Final/5B/Appl-sdE1_x_tdGFPmfas/Appl-sdE1_x_tdGFPmfas_Zoom_z=1.2um/Appl-sdE1_x_tdGFPmfas_z=1.2um_Zoom1.gif]

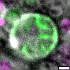

Supplement: Supplementary file 13 — Source data Fig. 5 [file 44318_2025_497_MOESM13_ESM.zip › EMBO_Figure5-Final/5B/Appl-sdE1_x_tdGFPmfas/Appl-sdE1_x_tdGFPmfas_Zoom_z=1.2um/Appl-sdE1_x_tdGFPmfas_z=1.2um_Zoom2.gif]

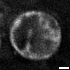

Supplement: Supplementary file 13 — Source data Fig. 5 [file 44318_2025_497_MOESM13_ESM.zip › EMBO_Figure5-Final/5B/Appl-sdE1_x_tdGFPmfas/Appl-sdE1_x_tdGFPmfas_Zoom_z=2.2um/Appl-sdE1_x_tdGFPmfas_z=2.2um_Zoom1.gif]

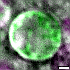

Supplement: Supplementary file 13 — Source data Fig. 5 [file 44318_2025_497_MOESM13_ESM.zip › EMBO_Figure5-Final/5B/Appl-sdE1_x_tdGFPmfas/Appl-sdE1_x_tdGFPmfas_Zoom_z=2.2um/Appl-sdE1_x_tdGFPmfas_z=2.2um_Zoom2.gif]

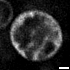

Supplement: Supplementary file 13 — Source data Fig. 5 [file 44318_2025_497_MOESM13_ESM.zip › EMBO_Figure5-Final/5B/Appl-sdE1_x_tdGFPmfas/Appl-sdE1_x_tdGFPmfas_Zoom_z=3.4um/Appl-sdE1_x_tdGFPmfas_z=3.4um_Zoom1.gif]

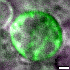

Supplement: Supplementary file 13 — Source data Fig. 5 [file 44318_2025_497_MOESM13_ESM.zip › EMBO_Figure5-Final/5B/Appl-sdE1_x_tdGFPmfas/Appl-sdE1_x_tdGFPmfas_Zoom_z=3.4um/Appl-sdE1_x_tdGFPmfas_z=3.4um_Zoom2.gif]

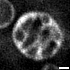

Supplement: Supplementary file 13 — Source data Fig. 5 [file 44318_2025_497_MOESM13_ESM.zip › EMBO_Figure5-Final/5B/Appl-sdE1_x_tdGFPmfas/Appl-sdE1_x_tdGFPmfas_Zoom_z=4.2um/Appl-sdE1_x_tdGFPmfas_z=4.2um_Zoom1.gif]

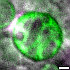

Supplement: Supplementary file 13 — Source data Fig. 5 [file 44318_2025_497_MOESM13_ESM.zip › EMBO_Figure5-Final/5B/Appl-sdE1_x_tdGFPmfas/Appl-sdE1_x_tdGFPmfas_Zoom_z=4.2um/Appl-sdE1_x_tdGFPmfas_z=4.2um_Zoom2.gif]

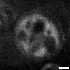

Supplement: Supplementary file 13 — Source data Fig. 5 [file 44318_2025_497_MOESM13_ESM.zip › EMBO_Figure5-Final/5B/Appl-sdE1_x_tdGFPmfas/Appl-sdE1_x_tdGFPmfas_Zoom_z=5.4um/Appl-sdE1_x_tdGFPmfas_z=5.4um_Zoom1.gif]

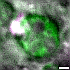

Supplement: Supplementary file 13 — Source data Fig. 5 [file 44318_2025_497_MOESM13_ESM.zip › EMBO_Figure5-Final/5B/Appl-sdE1_x_tdGFPmfas/Appl-sdE1_x_tdGFPmfas_Zoom_z=5.4um/Appl-sdE1_x_tdGFPmfas_z=5.4um_Zoom2.gif]

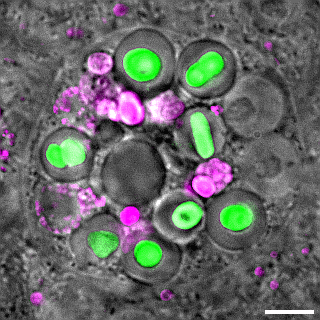

Supplement: Supplementary file 13 — Source data Fig. 5 [file 44318_2025_497_MOESM13_ESM.zip › EMBO_Figure5-Final/5B/Appl-WT_x_tdGFPmfas/Appl-WT_x_tdGFPmfas_Composite.gif]

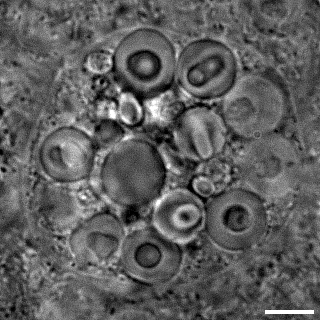

Supplement: Supplementary file 13 — Source data Fig. 5 [file 44318_2025_497_MOESM13_ESM.zip › EMBO_Figure5-Final/5B/Appl-WT_x_tdGFPmfas/Appl-WT_x_tdGFPmfas_DIC.gif]

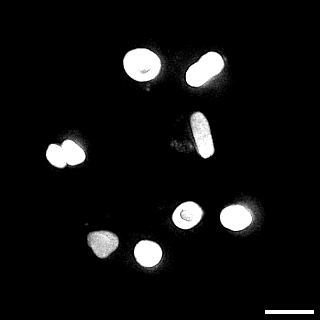

Supplement: Supplementary file 13 — Source data Fig. 5 [file 44318_2025_497_MOESM13_ESM.zip › EMBO_Figure5-Final/5B/Appl-WT_x_tdGFPmfas/Appl-WT_x_tdGFPmfas_GFP.gif]

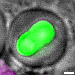

Supplement: Supplementary file 13 — Source data Fig. 5 [file 44318_2025_497_MOESM13_ESM.zip › EMBO_Figure5-Final/5B/Appl-WT_x_tdGFPmfas/Appl-WT_x_tdGFPmfas_Zoom1.gif]

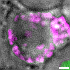

Supplement: Supplementary file 13 — Source data Fig. 5 [file 44318_2025_497_MOESM13_ESM.zip › EMBO_Figure5-Final/5B/Appl-WT_x_tdGFPmfas/Appl-WT_x_tdGFPmfas_Zoom2.gif]

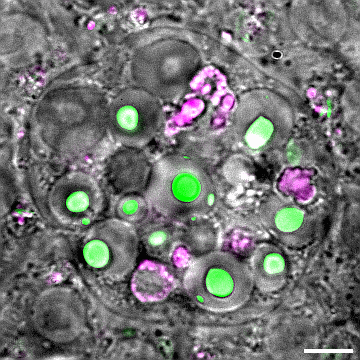

Supplement: Supplementary file 13 — Source data Fig. 5 [file 44318_2025_497_MOESM13_ESM.zip › EMBO_Figure5-Final/5B/w1118_x_tdGFPmfas/w1118_x_tdGFPmfas_Composite.gif]

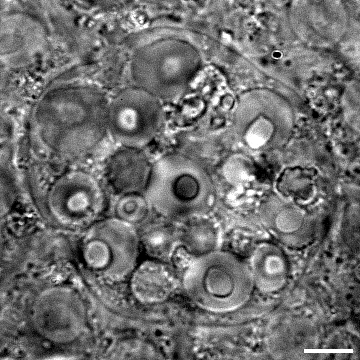

Supplement: Supplementary file 13 — Source data Fig. 5 [file 44318_2025_497_MOESM13_ESM.zip › EMBO_Figure5-Final/5B/w1118_x_tdGFPmfas/w1118_x_tdGFPmfas_DIC.gif]

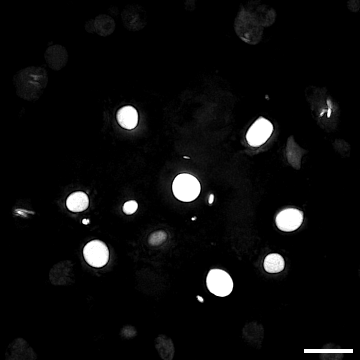

Supplement: Supplementary file 13 — Source data Fig. 5 [file 44318_2025_497_MOESM13_ESM.zip › EMBO_Figure5-Final/5B/w1118_x_tdGFPmfas/w1118_x_tdGFPmfas_GFP.gif]

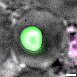

Supplement: Supplementary file 13 — Source data Fig. 5 [file 44318_2025_497_MOESM13_ESM.zip › EMBO_Figure5-Final/5B/w1118_x_tdGFPmfas/w1118_x_tdGFPmfas_Zoom1.gif]

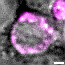

Supplement: Supplementary file 13 — Source data Fig. 5 [file 44318_2025_497_MOESM13_ESM.zip › EMBO_Figure5-Final/5B/w1118_x_tdGFPmfas/w1118_x_tdGFPmfas_Zoom2.gif]

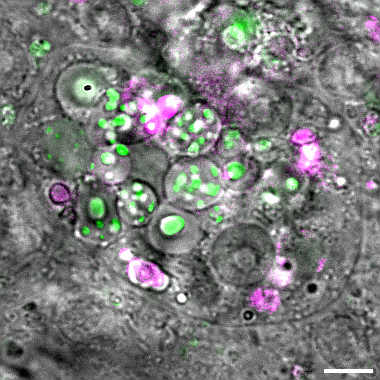

Supplement: Supplementary file 14 — Source data Fig. 6 [file 44318_2025_497_MOESM14_ESM.zip › EMBO_Figure6-Final/6A/Abeta_Dutch_x_tdGFPmfas/Abeta_Dutch_x_tdGFPmfas_Composite.gif]

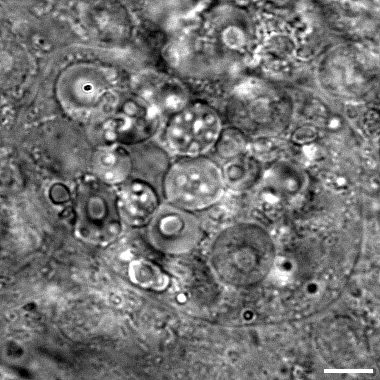

Supplement: Supplementary file 14 — Source data Fig. 6 [file 44318_2025_497_MOESM14_ESM.zip › EMBO_Figure6-Final/6A/Abeta_Dutch_x_tdGFPmfas/Abeta_Dutch_x_tdGFPmfas_DIC.gif]

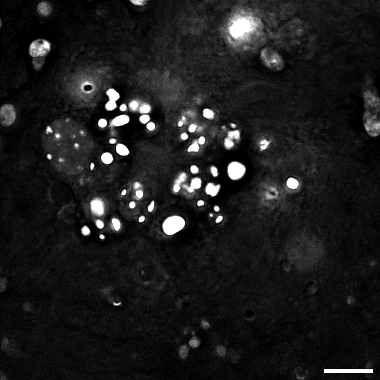

Supplement: Supplementary file 14 — Source data Fig. 6 [file 44318_2025_497_MOESM14_ESM.zip › EMBO_Figure6-Final/6A/Abeta_Dutch_x_tdGFPmfas/Abeta_Dutch_x_tdGFPmfas_GFP.gif]

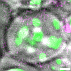

Supplement: Supplementary file 14 — Source data Fig. 6 [file 44318_2025_497_MOESM14_ESM.zip › EMBO_Figure6-Final/6A/Abeta_Dutch_x_tdGFPmfas/Abeta_Dutch_x_tdGFPmfas_Zoom1.gif]

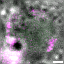

Supplement: Supplementary file 14 — Source data Fig. 6 [file 44318_2025_497_MOESM14_ESM.zip › EMBO_Figure6-Final/6A/Abeta_Dutch_x_tdGFPmfas/Abeta_Dutch_x_tdGFPmfas_Zoom2.gif]

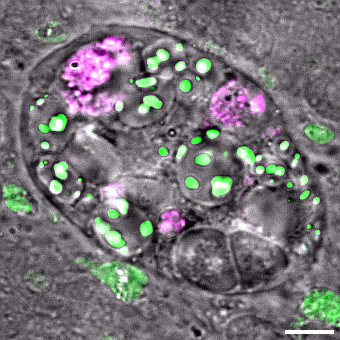

Supplement: Supplementary file 14 — Source data Fig. 6 [file 44318_2025_497_MOESM14_ESM.zip › EMBO_Figure6-Final/6A/Abeta_Iowa_x_tdGFPmfas/Abeta_Iowa_x_tdGFPmfas_Composite.gif]

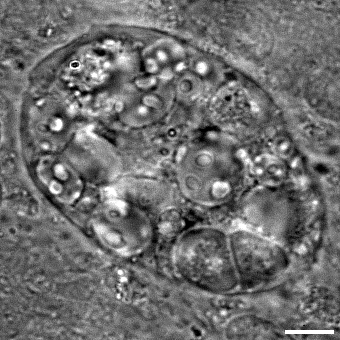

Supplement: Supplementary file 14 — Source data Fig. 6 [file 44318_2025_497_MOESM14_ESM.zip › EMBO_Figure6-Final/6A/Abeta_Iowa_x_tdGFPmfas/Abeta_Iowa_x_tdGFPmfas_DIC.gif]

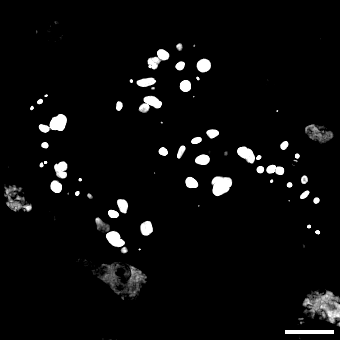

Supplement: Supplementary file 14 — Source data Fig. 6 [file 44318_2025_497_MOESM14_ESM.zip › EMBO_Figure6-Final/6A/Abeta_Iowa_x_tdGFPmfas/Abeta_Iowa_x_tdGFPmfas_GFP.gif]

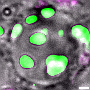

Supplement: Supplementary file 14 — Source data Fig. 6 [file 44318_2025_497_MOESM14_ESM.zip › EMBO_Figure6-Final/6A/Abeta_Iowa_x_tdGFPmfas/Abeta_Iowa_x_tdGFPmfas_Zoom1.gif]

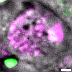

Supplement: Supplementary file 14 — Source data Fig. 6 [file 44318_2025_497_MOESM14_ESM.zip › EMBO_Figure6-Final/6A/Abeta_Iowa_x_tdGFPmfas/Abeta_Iowa_x_tdGFPmfas_Zoom2.gif]

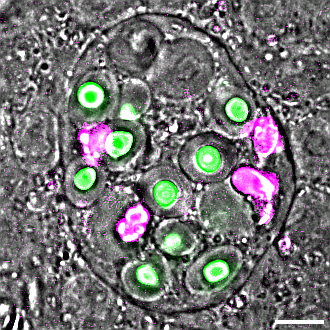

Supplement: Supplementary file 14 — Source data Fig. 6 [file 44318_2025_497_MOESM14_ESM.zip › EMBO_Figure6-Final/6A/Abeta_WT_x_tdGFPmfas/Abeta_WT_x_tdGFPmfas_Composite.gif]

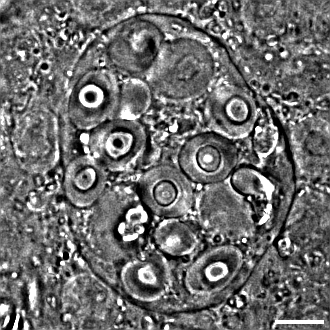

Supplement: Supplementary file 14 — Source data Fig. 6 [file 44318_2025_497_MOESM14_ESM.zip › EMBO_Figure6-Final/6A/Abeta_WT_x_tdGFPmfas/Abeta_WT_x_tdGFPmfas_DIC.gif]

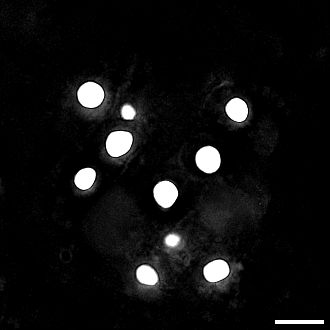

Supplement: Supplementary file 14 — Source data Fig. 6 [file 44318_2025_497_MOESM14_ESM.zip › EMBO_Figure6-Final/6A/Abeta_WT_x_tdGFPmfas/Abeta_WT_x_tdGFPmfas_GFP.gif]

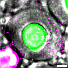

Supplement: Supplementary file 14 — Source data Fig. 6 [file 44318_2025_497_MOESM14_ESM.zip › EMBO_Figure6-Final/6A/Abeta_WT_x_tdGFPmfas/Abeta_WT_x_tdGFPmfas_Zoom1.gif]

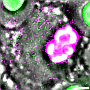

Supplement: Supplementary file 14 — Source data Fig. 6 [file 44318_2025_497_MOESM14_ESM.zip › EMBO_Figure6-Final/6A/Abeta_WT_x_tdGFPmfas/Abeta_WT_x_tdGFPmfas_Zoom2.gif]

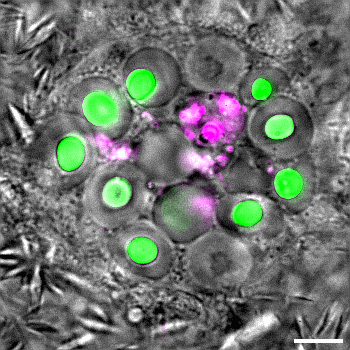

Supplement: Supplementary file 14 — Source data Fig. 6 [file 44318_2025_497_MOESM14_ESM.zip › EMBO_Figure6-Final/6A/w1118_x_tdGFPmfas/w1118_x_tdGFPmfas_Composite.gif]

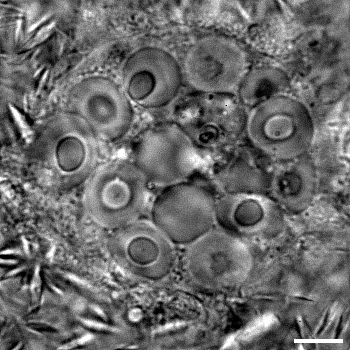

Supplement: Supplementary file 14 — Source data Fig. 6 [file 44318_2025_497_MOESM14_ESM.zip › EMBO_Figure6-Final/6A/w1118_x_tdGFPmfas/w1118_x_tdGFPmfas_DIC.gif]

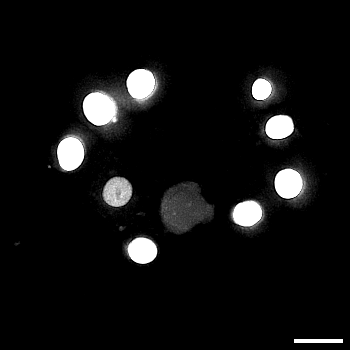

Supplement: Supplementary file 14 — Source data Fig. 6 [file 44318_2025_497_MOESM14_ESM.zip › EMBO_Figure6-Final/6A/w1118_x_tdGFPmfas/w1118_x_tdGFPmfas_GFP.gif]

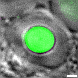

Supplement: Supplementary file 14 — Source data Fig. 6 [file 44318_2025_497_MOESM14_ESM.zip › EMBO_Figure6-Final/6A/w1118_x_tdGFPmfas/w1118_x_tdGFPmfas_Zoom1.gif]

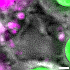

Supplement: Supplementary file 14 — Source data Fig. 6 [file 44318_2025_497_MOESM14_ESM.zip › EMBO_Figure6-Final/6A/w1118_x_tdGFPmfas/w1118_x_tdGFPmfas_Zoom2.gif]

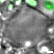

Supplement: Supplementary file 14 — Source data Fig. 6 [file 44318_2025_497_MOESM14_ESM.zip › EMBO_Figure6-Final/6F/Abeta_Dutch_x_tdGFPmfas_Biogenesis_movie_stills/Abeta_Dutch_x_tdGFPmfas_Biogenesis_movie_Composite_Zoom1_t=0.gif]

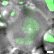

Supplement: Supplementary file 14 — Source data Fig. 6 [file 44318_2025_497_MOESM14_ESM.zip › EMBO_Figure6-Final/6F/Abeta_Dutch_x_tdGFPmfas_Biogenesis_movie_stills/Abeta_Dutch_x_tdGFPmfas_Biogenesis_movie_Composite_Zoom2_t=45.gif]
