# Supplementary figures and images for: Amyloid-β disrupts APP-regulated protein aggregation and dissociation from recycling endosomal membranes (part 3 of 3)
Source: EMBO J. 2025 Jul 17;44(16):4443–72. doi: 10.1038/s44318-025-00497-y (PMC12361456; doi:10.1038/s44318-025-00497-y)

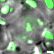

Supplement: Supplementary file 14 — Source data Fig. 6 [file 44318_2025_497_MOESM14_ESM.zip › EMBO_Figure6-Final/6F/Abeta_Dutch_x_tdGFPmfas_Biogenesis_movie_stills/Abeta_Dutch_x_tdGFPmfas_Biogenesis_movie_Composite_Zoom3_t=48.gif]

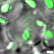

Supplement: Supplementary file 14 — Source data Fig. 6 [file 44318_2025_497_MOESM14_ESM.zip › EMBO_Figure6-Final/6F/Abeta_Dutch_x_tdGFPmfas_Biogenesis_movie_stills/Abeta_Dutch_x_tdGFPmfas_Biogenesis_movie_Composite_Zoom4_t=50.gif]

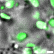

Supplement: Supplementary file 14 — Source data Fig. 6 [file 44318_2025_497_MOESM14_ESM.zip › EMBO_Figure6-Final/6F/Abeta_Dutch_x_tdGFPmfas_Biogenesis_movie_stills/Abeta_Dutch_x_tdGFPmfas_Biogenesis_movie_Composite_Zoom5_t=75.gif]

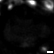

Supplement: Supplementary file 14 — Source data Fig. 6 [file 44318_2025_497_MOESM14_ESM.zip › EMBO_Figure6-Final/6F/Abeta_Dutch_x_tdGFPmfas_Biogenesis_movie_stills/Abeta_Dutch_x_tdGFPmfas_Biogenesis_movie_GFP_Zoom1_t=0.gif]

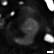

Supplement: Supplementary file 14 — Source data Fig. 6 [file 44318_2025_497_MOESM14_ESM.zip › EMBO_Figure6-Final/6F/Abeta_Dutch_x_tdGFPmfas_Biogenesis_movie_stills/Abeta_Dutch_x_tdGFPmfas_Biogenesis_movie_GFP_Zoom2_t=45.gif]

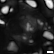

Supplement: Supplementary file 14 — Source data Fig. 6 [file 44318_2025_497_MOESM14_ESM.zip › EMBO_Figure6-Final/6F/Abeta_Dutch_x_tdGFPmfas_Biogenesis_movie_stills/Abeta_Dutch_x_tdGFPmfas_Biogenesis_movie_GFP_Zoom3_t=48.gif]

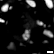

Supplement: Supplementary file 14 — Source data Fig. 6 [file 44318_2025_497_MOESM14_ESM.zip › EMBO_Figure6-Final/6F/Abeta_Dutch_x_tdGFPmfas_Biogenesis_movie_stills/Abeta_Dutch_x_tdGFPmfas_Biogenesis_movie_GFP_Zoom4_t=50.gif]

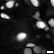

Supplement: Supplementary file 14 — Source data Fig. 6 [file 44318_2025_497_MOESM14_ESM.zip › EMBO_Figure6-Final/6F/Abeta_Dutch_x_tdGFPmfas_Biogenesis_movie_stills/Abeta_Dutch_x_tdGFPmfas_Biogenesis_movie_GFP_Zoom5_t=75.gif]

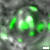

Supplement: Supplementary file 14 — Source data Fig. 6 [file 44318_2025_497_MOESM14_ESM.zip › EMBO_Figure6-Final/6F/Abeta_Dutch_x_tdGFPmfas_MatureCompartment_movie_stills/Abeta_Dutch_x_tdGFPmfas_MatureCompartment_movie_Composite_Zoom1_t=0.gif]

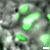

Supplement: Supplementary file 14 — Source data Fig. 6 [file 44318_2025_497_MOESM14_ESM.zip › EMBO_Figure6-Final/6F/Abeta_Dutch_x_tdGFPmfas_MatureCompartment_movie_stills/Abeta_Dutch_x_tdGFPmfas_MatureCompartment_movie_Composite_Zoom3_t=45.gif]

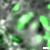

Supplement: Supplementary file 14 — Source data Fig. 6 [file 44318_2025_497_MOESM14_ESM.zip › EMBO_Figure6-Final/6F/Abeta_Dutch_x_tdGFPmfas_MatureCompartment_movie_stills/Abeta_Dutch_x_tdGFPmfas_MatureCompartment_movie_Composite_Zoom5_t=75.gif]

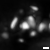

Supplement: Supplementary file 14 — Source data Fig. 6 [file 44318_2025_497_MOESM14_ESM.zip › EMBO_Figure6-Final/6F/Abeta_Dutch_x_tdGFPmfas_MatureCompartment_movie_stills/Abeta_Dutch_x_tdGFPmfas_MatureCompartment_movie_GFP_Zoom1_t=0.gif]

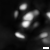

Supplement: Supplementary file 14 — Source data Fig. 6 [file 44318_2025_497_MOESM14_ESM.zip › EMBO_Figure6-Final/6F/Abeta_Dutch_x_tdGFPmfas_MatureCompartment_movie_stills/Abeta_Dutch_x_tdGFPmfas_MatureCompartment_movie_GFP_Zoom3_t=45.gif]

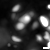

Supplement: Supplementary file 14 — Source data Fig. 6 [file 44318_2025_497_MOESM14_ESM.zip › EMBO_Figure6-Final/6F/Abeta_Dutch_x_tdGFPmfas_MatureCompartment_movie_stills/Abeta_Dutch_x_tdGFPmfas_MatureCompartment_movie_GFP_Zoom5_t=75.gif]

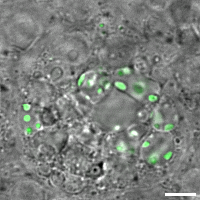

Supplement: Supplementary file 14 — Source data Fig. 6 [file 44318_2025_497_MOESM14_ESM.zip › EMBO_Figure6-Final/6F/Abeta_Dutch_x_tdGFPmfas_movie_whole-cell_Composite.gif]

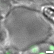

Supplement: Supplementary file 14 — Source data Fig. 6 [file 44318_2025_497_MOESM14_ESM.zip › EMBO_Figure6-Final/6G/Abeta_Iowa_x_tdGFPmfas_Biogenesis_movie_stills/Abeta_Iowa_x_tdGFPmfas_Biogenesis_movie_Composite_Zoom1_t=0.gif]

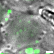

Supplement: Supplementary file 14 — Source data Fig. 6 [file 44318_2025_497_MOESM14_ESM.zip › EMBO_Figure6-Final/6G/Abeta_Iowa_x_tdGFPmfas_Biogenesis_movie_stills/Abeta_Iowa_x_tdGFPmfas_Biogenesis_movie_Composite_Zoom2_t=46.gif]

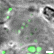

Supplement: Supplementary file 14 — Source data Fig. 6 [file 44318_2025_497_MOESM14_ESM.zip › EMBO_Figure6-Final/6G/Abeta_Iowa_x_tdGFPmfas_Biogenesis_movie_stills/Abeta_Iowa_x_tdGFPmfas_Biogenesis_movie_Composite_Zoom3_t=49.gif]

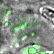

Supplement: Supplementary file 14 — Source data Fig. 6 [file 44318_2025_497_MOESM14_ESM.zip › EMBO_Figure6-Final/6G/Abeta_Iowa_x_tdGFPmfas_Biogenesis_movie_stills/Abeta_Iowa_x_tdGFPmfas_Biogenesis_movie_Composite_Zoom4_t=65.gif]

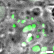

Supplement: Supplementary file 14 — Source data Fig. 6 [file 44318_2025_497_MOESM14_ESM.zip › EMBO_Figure6-Final/6G/Abeta_Iowa_x_tdGFPmfas_Biogenesis_movie_stills/Abeta_Iowa_x_tdGFPmfas_Biogenesis_movie_Composite_Zoom5_t=165.gif]

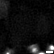

Supplement: Supplementary file 14 — Source data Fig. 6 [file 44318_2025_497_MOESM14_ESM.zip › EMBO_Figure6-Final/6G/Abeta_Iowa_x_tdGFPmfas_Biogenesis_movie_stills/Abeta_Iowa_x_tdGFPmfas_Biogenesis_movie_GFP_Zoom1_t=0.gif]

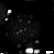

Supplement: Supplementary file 14 — Source data Fig. 6 [file 44318_2025_497_MOESM14_ESM.zip › EMBO_Figure6-Final/6G/Abeta_Iowa_x_tdGFPmfas_Biogenesis_movie_stills/Abeta_Iowa_x_tdGFPmfas_Biogenesis_movie_GFP_Zoom2_t=46.gif]

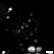

Supplement: Supplementary file 14 — Source data Fig. 6 [file 44318_2025_497_MOESM14_ESM.zip › EMBO_Figure6-Final/6G/Abeta_Iowa_x_tdGFPmfas_Biogenesis_movie_stills/Abeta_Iowa_x_tdGFPmfas_Biogenesis_movie_GFP_Zoom3_t=49.gif]

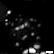

Supplement: Supplementary file 14 — Source data Fig. 6 [file 44318_2025_497_MOESM14_ESM.zip › EMBO_Figure6-Final/6G/Abeta_Iowa_x_tdGFPmfas_Biogenesis_movie_stills/Abeta_Iowa_x_tdGFPmfas_Biogenesis_movie_GFP_Zoom4_t=65.gif]

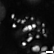

Supplement: Supplementary file 14 — Source data Fig. 6 [file 44318_2025_497_MOESM14_ESM.zip › EMBO_Figure6-Final/6G/Abeta_Iowa_x_tdGFPmfas_Biogenesis_movie_stills/Abeta_Iowa_x_tdGFPmfas_Biogenesis_movie_GFP_Zoom5_t=165.gif]

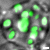

Supplement: Supplementary file 14 — Source data Fig. 6 [file 44318_2025_497_MOESM14_ESM.zip › EMBO_Figure6-Final/6G/Abeta_Iowa_x_tdGFPmfas_MatureCompartment_movie_stills/Abeta_Iowa_x_tdGFPmfas_MatureCompartment_movie_Composite_Zoom1_t=0.gif]

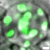

Supplement: Supplementary file 14 — Source data Fig. 6 [file 44318_2025_497_MOESM14_ESM.zip › EMBO_Figure6-Final/6G/Abeta_Iowa_x_tdGFPmfas_MatureCompartment_movie_stills/Abeta_Iowa_x_tdGFPmfas_MatureCompartment_movie_Composite_Zoom2_t=46.gif]

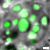

Supplement: Supplementary file 14 — Source data Fig. 6 [file 44318_2025_497_MOESM14_ESM.zip › EMBO_Figure6-Final/6G/Abeta_Iowa_x_tdGFPmfas_MatureCompartment_movie_stills/Abeta_Iowa_x_tdGFPmfas_MatureCompartment_movie_Composite_Zoom5_t=165.gif]

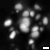

Supplement: Supplementary file 14 — Source data Fig. 6 [file 44318_2025_497_MOESM14_ESM.zip › EMBO_Figure6-Final/6G/Abeta_Iowa_x_tdGFPmfas_MatureCompartment_movie_stills/Abeta_Iowa_x_tdGFPmfas_MatureCompartment_movie_GFP_Zoom1_t=0.gif]

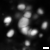

Supplement: Supplementary file 14 — Source data Fig. 6 [file 44318_2025_497_MOESM14_ESM.zip › EMBO_Figure6-Final/6G/Abeta_Iowa_x_tdGFPmfas_MatureCompartment_movie_stills/Abeta_Iowa_x_tdGFPmfas_MatureCompartment_movie_GFP_Zoom2_t=46.gif]

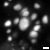

Supplement: Supplementary file 14 — Source data Fig. 6 [file 44318_2025_497_MOESM14_ESM.zip › EMBO_Figure6-Final/6G/Abeta_Iowa_x_tdGFPmfas_MatureCompartment_movie_stills/Abeta_Iowa_x_tdGFPmfas_MatureCompartment_movie_GFP_Zoom5_t=165.gif]

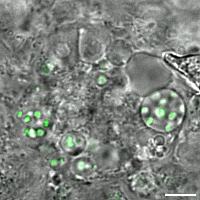

Supplement: Supplementary file 14 — Source data Fig. 6 [file 44318_2025_497_MOESM14_ESM.zip › EMBO_Figure6-Final/6G/Abeta_Iowa_x_tdGFPmfas_movie_whole-cell_Composite.gif]
