# Supplementary material for: Amyloid-β disrupts APP-regulated protein aggregation and dissociation from recycling endosomal membranes
Source: EMBO J. 2025 Jul 17;44(16):4443–72. doi: 10.1038/s44318-025-00497-y (PMC12361456; doi:10.1038/s44318-025-00497-y)
Supplement: Supplementary file 15 — Expanded View Figures [file 44318_2025_497_MOESM15_ESM.pdf]

Expanded View Figures

Figure EV1. *Drosophila* MFAS selectively drives DCG assembly in SCs, related to Fig. 1.

(**A–C**) Ex vivo, wide-field fluorescence micrographs and DIC images of SCs from 6-day-old males expressing SC-specific *rosy*-RNAi or either of two independent *mfas*-RNAis with the *GFP-mfas* gene trap (**A**), *YFP-Rab11* (**B**) or *CFP-Rab6* (**C**). Rab-positive ILVs (green arrowheads) in compartments are marked in Zoom panels. In *mfas* knockdown cells, sporadic intra-compartmental puncta detected by DIC, which are often Rab-positive, are marked with grey arrowheads. (**D**) Bar chart of DCG compartment number shows that expressing *rosy*-RNAi in adult SCs using the *tub-GAL80*^{ts}*; dsx-GAL4, GFP-mfas* SC-specific GAL4 driver line has no effect relative to controls. (**E**) Stills from a time-lapse movie of DCG biogenesis and DCG acidification in SC from a 6-day-old male expressing *GFP-mfas* gene trap. For the compartment marked by the white box (top row of zoomed images), a single DCG forms rapidly from a GFP-MFAS cloud. For compartment marked by white box and asterisk (bottom row), small LysoTracker Red-positive compartments (red arrowheads) contact and spread around the periphery of the DCG compartment, then start to acidify the lumen around the DCG (66 min) before rapid dispersion of the core (67.5 min; GFP and by DIC). Purple arrowhead marks DCG; it can persist for 20 min following the start of the acidification process. *td-GFP-mfas* = *tub-GAL80*^{ts}*/+; dsx-GAL4, GFP-mfas/+*. (**F**, **G**) Knockdown of *mfas* with two independent RNAis has no effect on the number of Rab6-positive large compartments (**F**) or the proportion of these compartments containing Rab6-positive ILVs (**G**). (**H**) Knockdown of *mfas* in SCs expressing GFP-GPI, a DCG and a membrane marker, produces compartments with GFP at the limiting membrane, like controls and with internal concentrations of GFP in puncta that are also detected by DIC (grey arrowhead). (**I**) Bar charts showing proportion of large compartments with DCG acidification phenotype (**E**) in GFP-MFAS-expressing knockdown SCs. In all images, approximate cell boundaries and compartment boundaries are marked with a dashed white line; *n* = nuclei of binucleate cells; LysoTracker Red (magenta) marks acidic compartments. Scale bars = 5 and 1 µm in Zoom. For bar charts, data are mean ± SEM, analysed using the Kruskal–Wallis test, followed by Dunn's multiple comparisons post hoc test; *n* = animal number above bar, ***$P < 0.001$, ns not significant. In (I), *rosy* vs *mfas* #1, $P = 0.0010$; *rosy* vs *mfas* #2, $P = 0.0009$.

 © The Author(s)

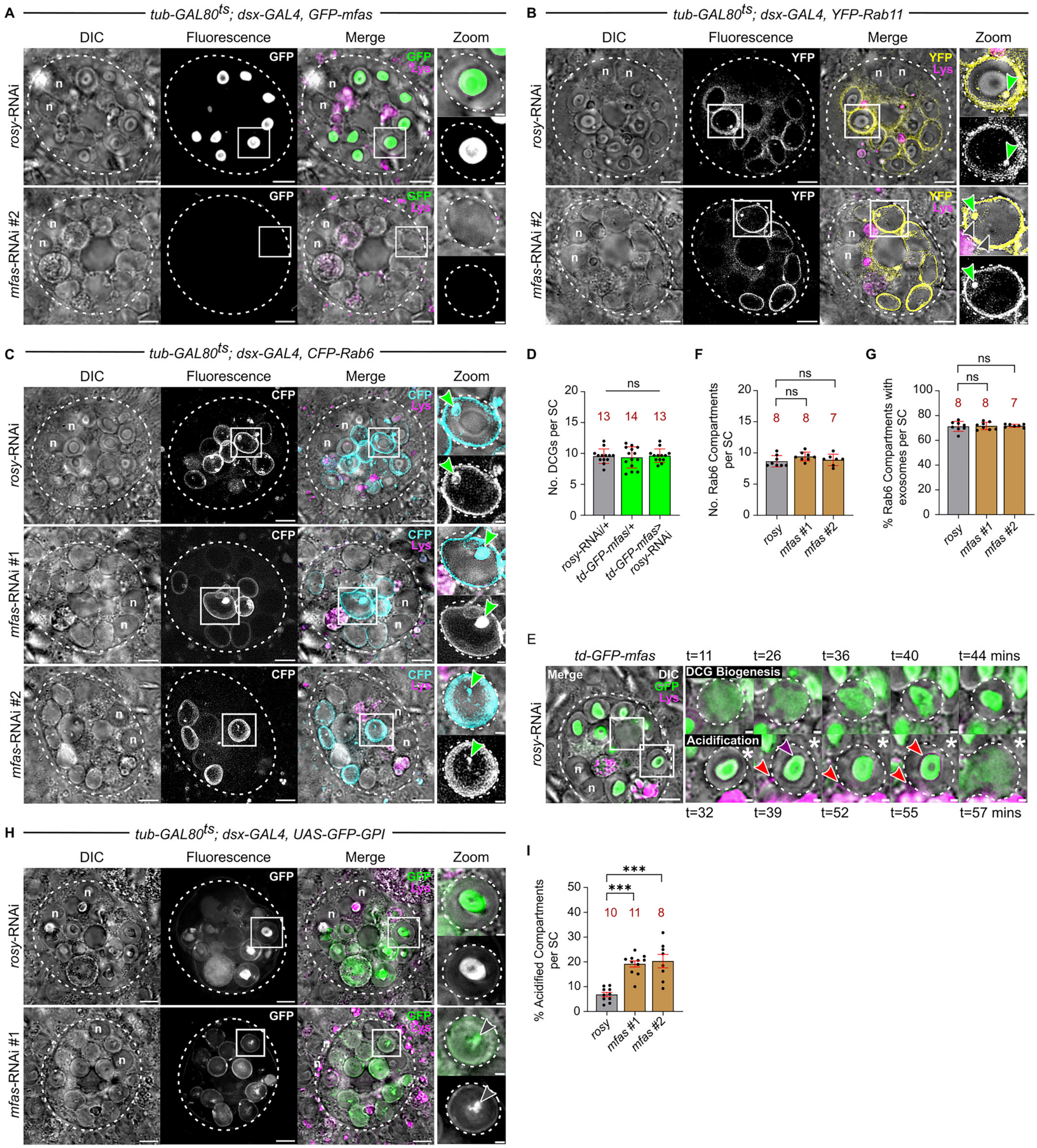
A
tub-GAL80ts; dsx-GAL4, GFP-mfas
DIC
Fluorescence
Merge
Zoom
rosy-RNAi
mfas-RNAi #2
GFP
Lys
n
B
tub-GAL80ts; dsx-GAL4, YFP-Rab11
YFP
C
tub-GAL80ts; dsx-GAL4, CFP-Rab6
CFP
mfas-RNAi #1
D
No. DCGs per SC
ns
13
14
13
rosy-RNAi/+
td-GFP-mfas/+
td-GFP-mfas> rosy-RNAi
F
No. Rab6 Compartments per SC
ns
8
8
7
rosy
mfas #1
mfas #2
G
% Rab6 Compartments with exosomes per SC
E
td-GFP-mfas
t=11
t=26
t=36
t=40
t=44 mins
Merge
DIC
DCG Biogenesis
Acidification
t=32
t=39
t=52
t=55
t=57 mins
H
tub-GAL80ts; dsx-GAL4, UAS-GFP-GPI
I
% Acidified Compartments per SC
***
10
11
8

© The Author(s)

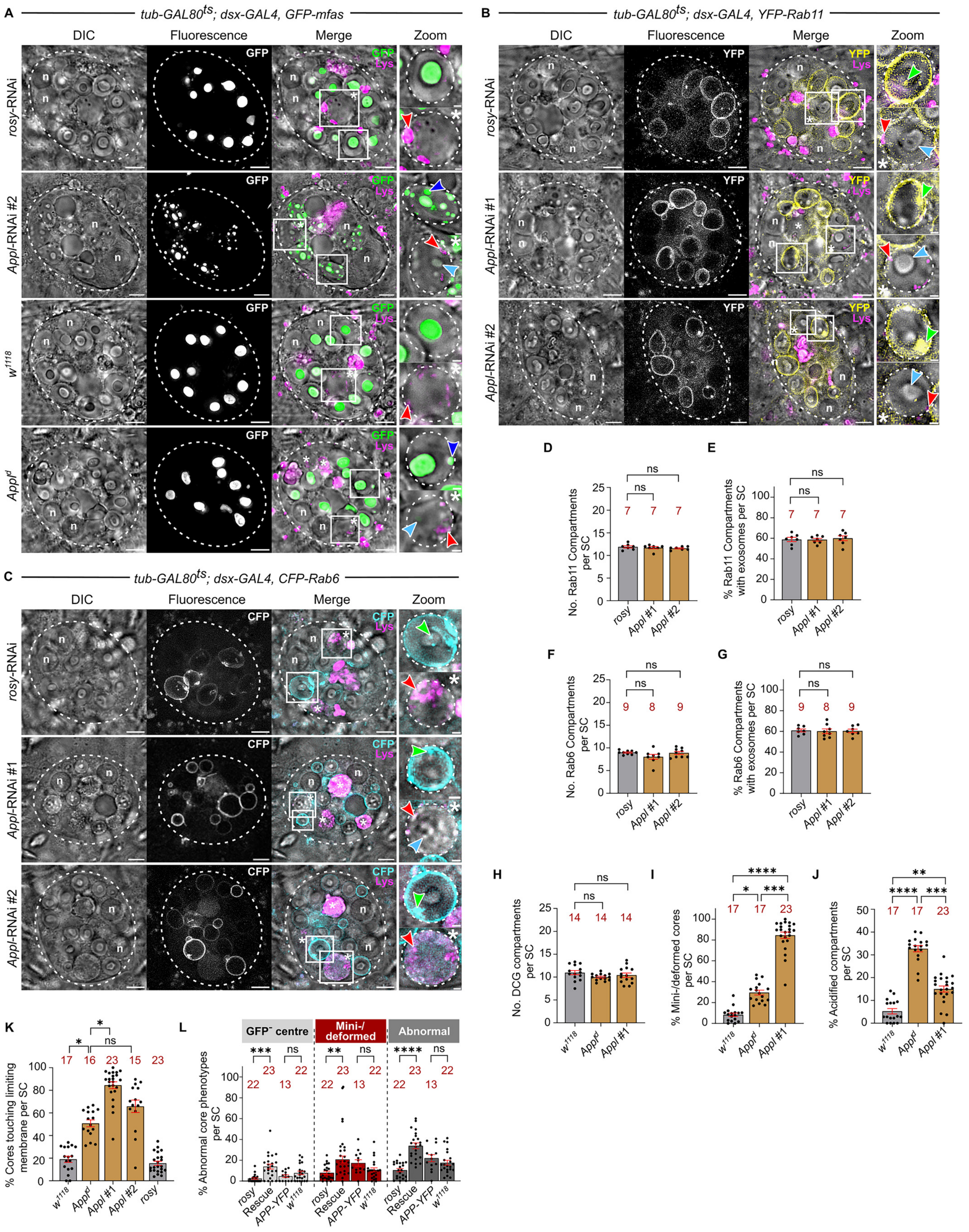
A
tub-GAL80ts; dsx-GAL4, GFP-mfas
DIC
Fluorescence
Merge
Zoom
rosy-RNAi
Appl-RNAi #2
w1118
Appld
GFP
Lys
B
tub-GAL80ts; dsx-GAL4, YFP-Rab11
rosy-RNAi
Appl-RNAi #1
Appl-RNAi #2
YFP
C
tub-GAL80ts; dsx-GAL4, CFP-Rab6
CFP
D
No. Rab11 Compartments per SC
E
% Rab11 Compartments with exosomes per SC
F
No. Rab6 Compartments per SC
G
% Rab6 Compartments with exosomes per SC
rosy
Appl #1
Appl #2
ns
H
No. DCG compartments per SC
I
% Mini-/deformed cores per SC
J
% Acidified compartments per SC
K
% Cores touching limiting membrane per SC
L
% Abnormal core phenotypes per SC
GFP- centre
Mini-/ deformed
Abnormal
Rescue
APP-YFP

 © The Author(s)

Figure EV2. *Drosophila* APPL regulates formation of large DCGs in SCs, related to Fig. 3.

(**A**) SCs expressing *GFP-mfas* gene trap and SC-specific *rosy*-RNAi or *Appl*-RNAi #2 (top two rows), or without additional transgenes, but in a control w^{1118} or $Appl^{d}$ mutant background (bottom two rows). Note that following knockdown of *Appl*, large secretory compartments contain multiple mini-cores (blue arrowheads), labelled with GFP-MFAS and visible by DIC (white box and upper Zoom panel). $Appl^{d}$ mutant SCs contain distorted DCGs that frequently contact the compartment's limiting membrane and sporadic mini-cores (white box, top Zoom panel). The DCG acidification phenotype is also more commonly observed in *Appl* knockdown and *Appl* mutant backgrounds (white asterisks and white boxes marked by asterisks, shown in lower Zoom panels; red arrowheads mark acidic microdomains). DCGs that have not yet been dissipated are marked with light blue arrowheads. (**B**, **C**) SCs expressing SC-specific *rosy*-RNAi or either of two independent *Appl*-RNAis with *YFP-Rab11* (**B**) or *CFP-Rab6* (**C**). Zoom panels show mini-core phenotype (white box, top panel) with intra-compartmental Rab puncta (green arrowheads), and DCG acidification phenotype with no Rab association (white box marked with an asterisk, bottom panel). (**D–G**) Bar charts showing numbers of Rab11-positive (**D**) or Rab6-positive (**F**) large compartments, and proportion of these compartments containing Rab11-positive (**E**) or Rab6-positive (**G**) ILVs in SCs with knockdown of *Appl* using two independent RNAis versus controls. (**H–K**) Bar charts comparing the effects of the $Appl^{d}$ null mutant with SC-specific knockdown of *Appl* and controls. $Appl^{d}$ typically does not affect DCG compartment number (**H**) or induce mini-core formation, but DCGs are often deformed (**I**) and touch the compartment's limiting membrane (**K**). The DCG acidification phenotype is particularly prominent in $Appl^{d}$ SCs (**J**). (**L**) Bar chart showing DCG phenotypes induced by APP-YFP expression with and without *Appl* knockdown versus control SCs. Note that in *Appl* knockdown cells rescued by APP-YFP, about 10% of DCGs are mis-assembled, lacking GFP-MFAS at their centre (GFP-centre), perhaps because high-efficiency APP-YFP cleavage is required to prime the aggregation of proteins at the centre of all compartments in this genetic background. In all images, n nuclei; LysoTracker Red (magenta) marks acidic compartments. Scale bars = 5 and 1 µm in Zoom. For bar charts, data are mean ± SEM, analysed using the Kruskal–Wallis test; n = animal number above bar, *$P < 0.05$, **$P < 0.01$, ***$P < 0.001$, ****$P < 0.0001$, ns not significant. In (**I**), w^{1118} vs $Appl^{d}$, P = 0.014; $Appl^{d}$ vs *Appl* #1, $P = 0.0004$. In (**J**), w^{1118} vs *Appl* #1, $P = 0.0070$; $Appl^{d}$ vs *Appl* #1, $P = 0.0002$. In (**K**), w^{1118} vs $Appl^{d}$, $P = 0.032$; $Appl^{d}$ vs *Appl* #1, $P = 0.024$. In (**L**), *rosy* vs Rescue (GFP-centre), $P = 0.0001$; *rosy* vs Rescue (Mini-/deformed), $P = 0.0058$.

© The Author(s)

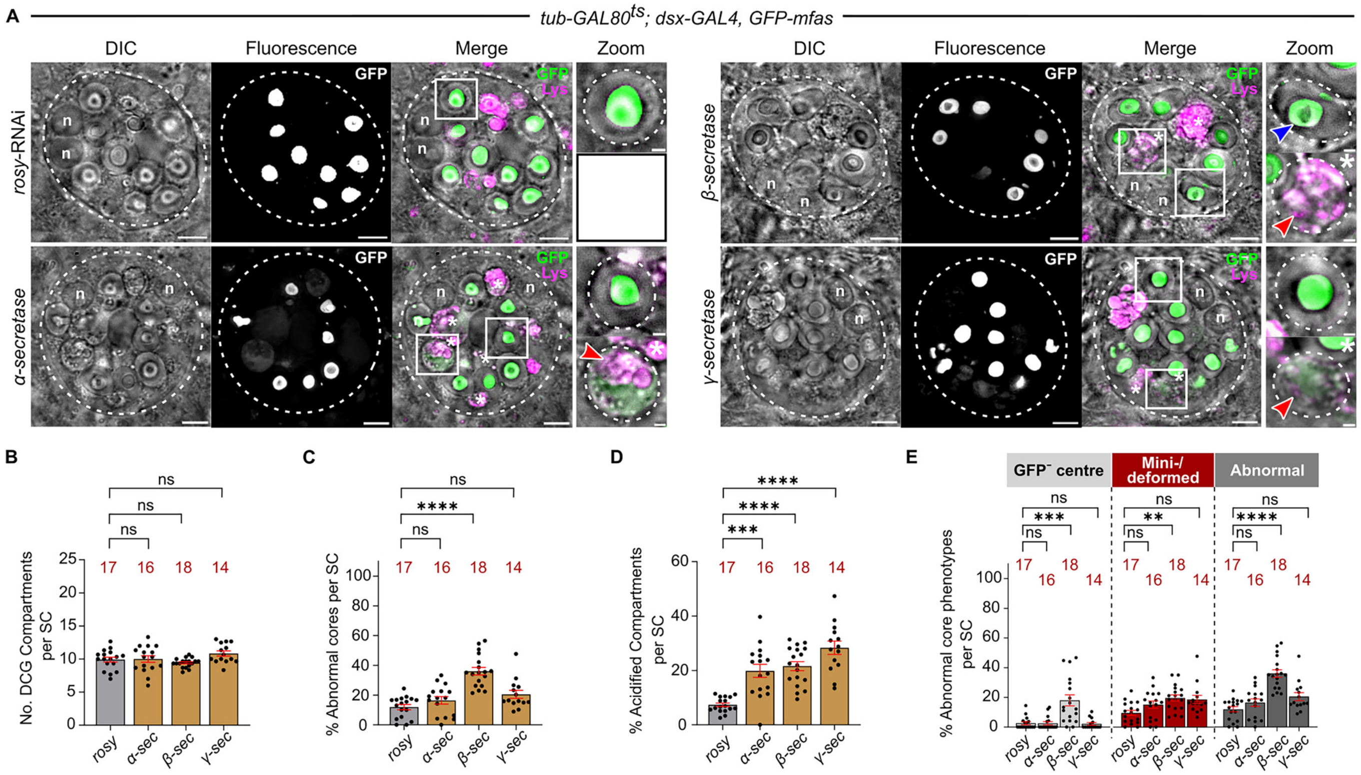

Figure EV3. Secretases involved in *Drosophila* APPL processing regulate DCG maturation, related to Fig. 5.

(**A**) SCs expressing *GFP-mfas* gene trap and SC-specific *rosy*-RNAi or RNAi targeting *α*-, *β*- and *γ-secretases*. Note β-secretase knockdown affects DCG morphology, so that GFP-MFAS is frequently absent from a large central region within the DCG, while other secretase knockdowns rarely affect DCG morphology (shown for compartments outlined with white boxes in upper Zoom). White asterisks and white boxes marked with asterisks in the Merge channel indicate DCG compartments with acidification phenotype (lower Zoom; red arrowheads mark acidic microdomains). (**B–E**) Bar charts showing effects of knockdowns on the number of DCG compartments (**B**), and proportion of abnormal DCGs (**C**). A greater proportion of large compartments display the DCG acidification phenotype following secretase knockdown than in controls (**D**). The different DCG phenotypes observed following knockdown of *α*-, *β*- and *γ-secretases* are categorised in (**E**). Note that *β-secretase* knockdown induces the formation of DCGs that lack GFP-MFAS at their centre. In all images, n nuclei; LysoTracker Red (magenta) marks acidic compartments. Scale bars = 5 and 1 μm in Zoom. For bar charts, data are mean ± SEM, analysed using the Kruskal–Wallis test; n = animal number above bar, $^{**}P < 0.01$, $^{***}P < 0.001$, $^{****}P < 0.0001$, ns not significant. In (**D**), *rosy* vs *α-sec*, $P = 0.0009$. In (**E**), *rosy* vs *β-sec* (GFP-centre), $P = 0.0008$; *rosy* vs *β-sec* (Mini-/deformed), $P = 0.0048$.

 © The Author(s)

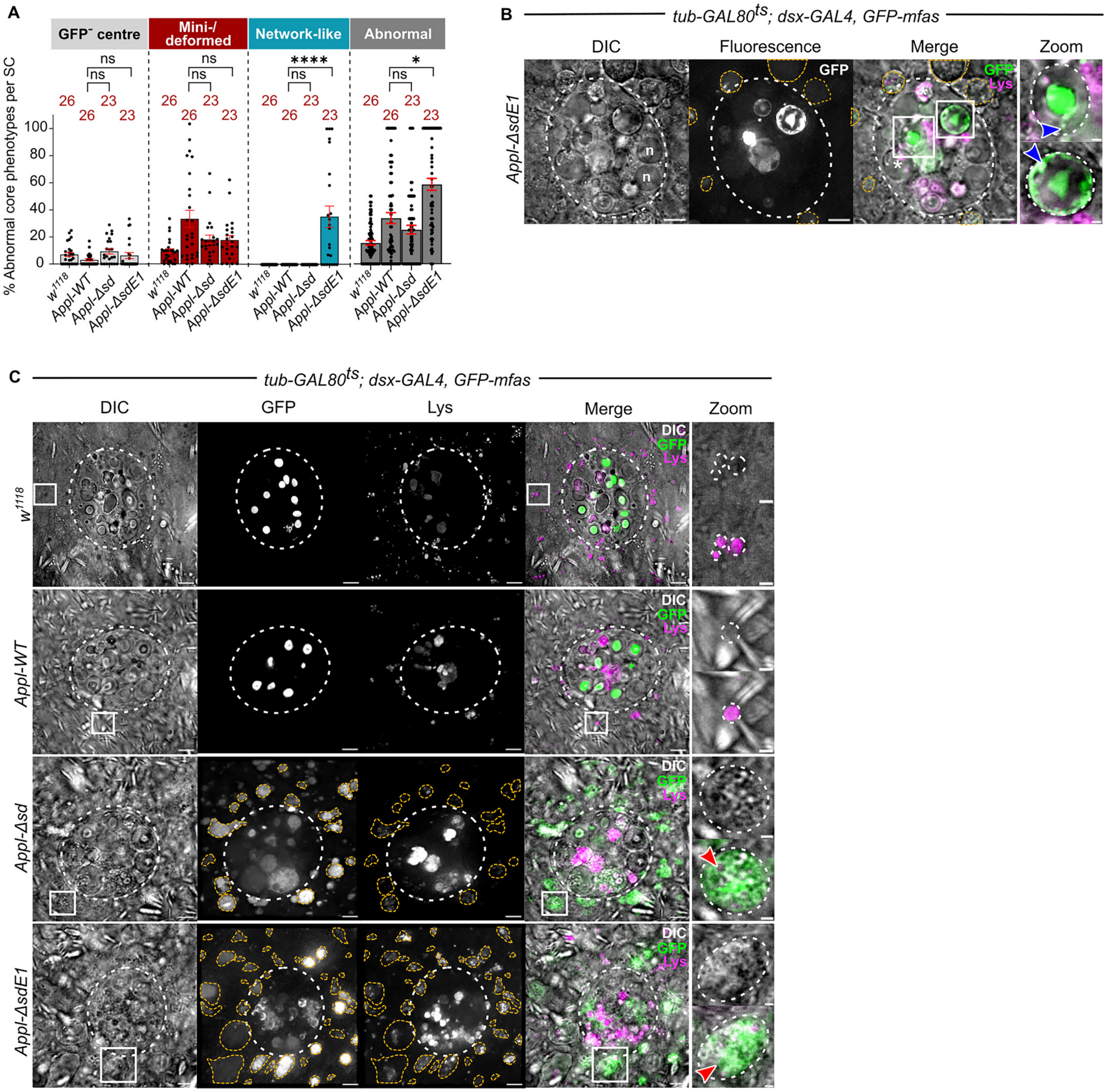

Figure EV4. *Drosophila* APPL and its cleavage regulate normal DCG formation, and the uptake of GFP-MFAS by other cells, related to Fig. 5.

(**A**) Bar chart showing that overexpression of APPL-WT, APPL-Δsd and APPL-ΔsdE1 produces abnormal DCGs, with APPL-ΔsdE1 generating a unique network phenotype. (**B**) SCs expressing *GFP-mfas* gene trap and APPL-ΔsdE1 (Fig. 4B). Note two abnormal DCG compartments that have a central abnormally shaped DCG, but also contain peripheral GFP-MFAS aggregates (blue arrowheads in compartments outlined with white boxes shown in Zoom panels). (**C**) SCs and surrounding main cells expressing *GFP-mfas* gene trap alone or with wild-type APPL (APPL-WT), APPL-Δsd or APPL-ΔsdE1 in SCs. Note for mutant APPL expression, abnormal accumulation of GFP-MFAS in main cell compartments that typically exhibit limited LysoTracker Red staining (compartments outlined by orange dashed lines); one example is outlined by a white box and shown in Zoom panels (DIC alone and DIC/Merge; red arrowheads mark acidic microdomains). In all images, n = nuclei; LysoTracker Red (magenta) marks acidic compartments in (**B**, **C**). Scale bars = 5 and 1 µm in Zoom. For bar charts, data are mean ± SEM, analysed using the Kruskal–Wallis test; n = animal number above bar, *$P < 0.05$, ****$P < 0.0001$, ns not significant. In (**A**), *Appl-WT* vs *Appl-ΔsdE1* (Abnormal), $P = 0.015$.

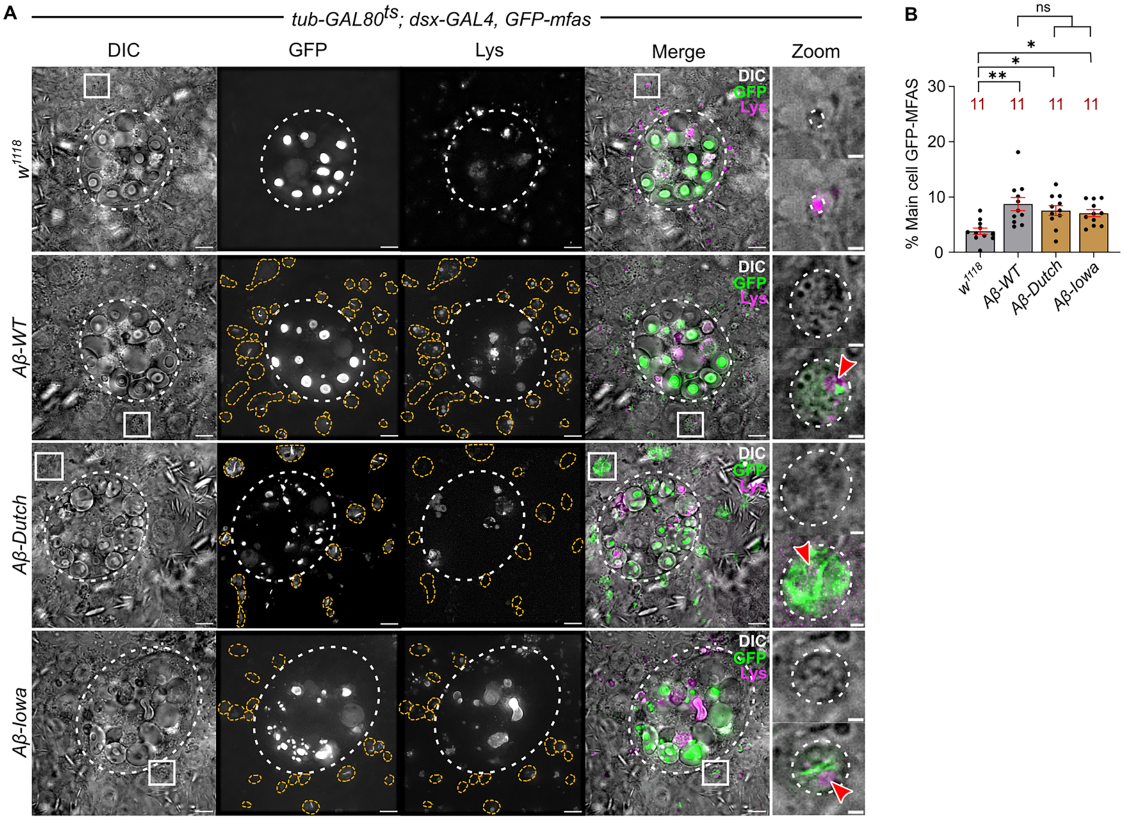

Figure EV5. Aβ-42-peptide expression in SCs promotes uptake of GFP-MFAS by other accessory gland cells, related to Fig. 6.

(**A**) SCs and surrounding main cells expressing *GFP-mfas* gene trap alone or with wild-type Aβ-42-peptide, or either the Iowa or Dutch mutant Aβ-42 peptides. Note for Aβ-42-peptide expression, abnormal accumulation of GFP-MFAS in main cell compartments that typically exhibit limited LysoTracker Red staining (compartments outlined by orange dashed lines); one example is outlined by a white box and shown in Zoom panels (DIC alone and DIC/Merge; red arrowheads mark acidic microdomains). (**B**) Bar chart showing the accumulation of GFP-MFAS in main cells of 6-day-old males overexpressing SC-specific wild type Aβ-42-peptide, or either the Iowa or Dutch mutant Aβ-42 peptides or w^{1118} controls, expressed as percentage of total main cell area that contains GFP. In all images, n nuclei; LysoTracker Red (magenta) marks acidic compartments in (**A**). Scale bars = 5 and 1 µm in Zoom. For the bar chart, data are mean ± SEM, analysed using the Kruskal–Wallis test; *n* = animal number above bar, **P* < 0.05, ***P* < 0.01, ns = not significant. In (**B**), w^{1118} vs *Aβ-WT*, *P* = 0.0029; w^{1118} vs *Aβ-Dutch*, *P* = 0.011; w^{1118} vs *Aβ-Iowa*, *P* = 0.048.

 © The Author(s)
